# Comprehensive analysis of prognostic significance of cadherin (CDH) gene family in breast cancer

**DOI:** 10.18632/aging.204357

**Published:** 2022-10-25

**Authors:** Su-Chi Ku, Hsin-Liang Liu, Che-Yu Su, I-Jeng Yeh, Meng-Chi Yen, Gangga Anuraga, Hoang Dang Khoa Ta, Chung-Chieh Chiao, Do Thi Minh Xuan, Fidelia Berenice Prayugo, Wei-Jan Wang, Chih-Yang Wang

**Affiliations:** 1 Graduate Institute of Cancer Biology and Drug Discovery, College of Medical Science and Technology, Taipei Medical University, Taipei 11031, Taiwan; 2 Ph.D. Program for Cancer Molecular Biology and Drug Discovery, College of Medical Science and Technology, Taipei Medical University and Academia Sinica, Taipei 11031, Taiwan; 3 Department of General Medicine, Taipei Medical University Hospital, Taipei 11031, Taiwan; 4 Department of Emergency Medicine, Kaohsiung Medical University Hospital, Kaohsiung Medical University, Kaohsiung 80708, Taiwan; 5 Graduate Institute of Clinical Medicine, College of Medicine, Kaohsiung Medical University, Kaohsiung 80708, Taiwan; 6 Department of Statistics, Faculty of Science and Technology, Universitas PGRI Adi Buana, Surabaya 60234, Indonesia; 7 International Master/PhD Program in Medicine, College of Medicine, Taipei Medical University, Taipei 11031, Taiwan; 8 Department of Biological Science and Technology, Research Center for Cancer Biology, China Medical University, Taichung 406040, Taiwan; 9 Research Center for Cancer Biology, China Medical University, Taichung 40676, Taiwan; 10 TMU Research Center of Cancer Translational Medicine, Taipei Medical University, Taipei 11031, Taiwan

**Keywords:** cadherin, bioinformatics, prognosis, breast cancer, therapeutic targets

## Abstract

Breast cancer is one of the leading deaths in all kinds of malignancies; therefore, it is important for early detection. At the primary tumor site, tumor cells could take on mesenchymal properties, termed the epithelial-to-mesenchymal transition (EMT). This process is partly regulated by members of the cadherin (*CDH*) family of genes, and it is an essential step in the formation of metastases. There has been a lot of study of the roles of some of the *CDH* family genes in cancer; however, a holistic approach examining the roles of distinct *CDH* family genes in the development of breast cancer remains largely unexplored. In the present study, we used a bioinformatics approach to examine expression profiles of *CDH* family genes using the Oncomine, Gene Expression Profiling Interactive Analysis 2 (GEPIA2), cBioPortal, MetaCore, and Tumor IMmune Estimation Resource (TIMER) platforms. We revealed that *CDH1/2/4/11/12/13* messenger (m)RNA levels are overexpressed in breast cancer cells compared to normal cells and were correlated with poor prognoses in breast cancer patients’ distant metastasis-free survival. An enrichment analysis showed that high expressions of *CDH1/2/4/11/12/13* were significantly correlated with cell adhesion, the extracellular matrix remodeling process, the EMT, WNT/beta-catenin, and interleukin-mediated immune responses. Collectively, *CDH1/2/4/11/12/13* are thought to be potential biomarkers for breast cancer progression and metastasis.

## INTRODUCTION

Breast cancer is one of the most common malignancies among women and the second leading cause of death after lung cancer [1, 2]. The prognosis of breast cancer is better with early detection and improved treatment. Because of the poor prognosis of advanced breast cancer, research on breast cancer has recently focused on precise detection of invasion and metastasis with accurate tumorigenic biomarkers [3–9]. Despite progress in developing diagnostic screening tools, distant metastases at the time of diagnosis indicates a worse prognosis with only 23% of patients surviving 5 years post-diagnosis [10]. Therefore, novel research on genetic alterations and signal transduction pathways is playing important roles in both early breast cancer detection and treatment in advanced stages [11–13].

The cadherins (CDHs) are a superfamily of calcium-dependent adhesion molecules which have functions in cell recognition, tissue morphogenesis, and tumor suppression [14, 15]. The *CDH* family consists of 23 members, from *CDH1* to *CDH26,* as documented in the GeneCards database [16]. Basic characteristics of the *CDH* gene family, including gene IDs and aliases, are presented in Table 1. Classic cadherins have mostly been thoroughly studied, including epithelial (E)-cadherin (*CDH1*), neural (N)-cadherin (*CDH2*), placental (P)-cadherin (*CDH3*), and retinal (R)-cadherin (*CDH4*) [17].

**Table 1:** Basic characteristics of the CDH gene family.

**Approved symbol**	**HGNC ID**	**Gene ID**	**Aliases**	**Location on chromosome**
CDH1	1748	999	Cadherin 1; Uvomorulin; CD324; UVO; Cadherin 1, Type 1, E-Cadherin (Epithelial)	16q22.1
CDH2	1759	1000	Cadherin 2; CDHN; CD325; NCAD; Cadherin 2, Type 1, N-Cadherin (Neuronal)	18q12.1
CDH3	1762	1001	Cadherin 3; CDHP; PCAD; Cadherin 3, Type 1, P-Cadherin (Placental)	16q22.1
CDH4	1763	1002	Cadherin 4; R-Cadherin; Cadherin 4, Type 1, R-Cadherin (Retinal)	20q13.33
CDH5	1764	1003	Cadherin 5; VE-Cadherin; CD144; 7B4	16q21
CDH6	1765	1004	Cadherin 6; Cadherin 6, Type 2, K-Cadherin (Fetal Kidney)	5p13.3
CDH7	1766	1005	Cadherin 7; Cadherin 7, Type 2; Cadherin-7; CDH7L1	18q22.1
CDH8	1767	1006	Cadherin 8; Cadherin 8, Type 2; Cadherin-8; Nbla04261	16q21
CDH9	1768	1007	Cadherin 9; Cadherin-9; Cadherin 9, Type 2 (T1-Cadherin)	5p14.1
CDH10	1749	1008	Cadherin 10; Cadherin-10; T2-Cadherin; Cadherin 10, Type 2, (T2-Cadherin)	5p14.2-p14.1
CDH11	1750	1009	Cadherin 11; CAD11; OB; Cadherin 11, Type 2, OB-Cadherin (Osteoblast)	16q21
CDH12	1751	1010	Cadherin 12; Br-Cadherin; CDHB; Neural Type Cadherin 2	5p14.3
CDH13	1753	1012	Cadherin 13; CDHH; T-Cadherin; H-Cadherin (Heart)	16q23.3
CDH15	1754	1013	Cadherin 15; CDH 14; CDH3; Cadherin 15, Type 1, M-Cadherin (Myotubule)	16q24.3
CDH16	1755	1014	Cadherin 16; Cadherin 16, KSP-Cadherin; Kidney-Specific Cadherin	16q22.1
CDH17	1756	1015	Cadherin 17; HPT-1; Intestinal Peptide-Associated Transporter HPT-1	8q22.1
CDH18	1757	1016	Cadherin 18; CDH14; Cadherin 18, Type 2	5p14.3
CDH19	1758	28513	Cadherin 19; CDH7; Cadherin 19, Type 2	18q22.1
CDH20	1760	28316	Cadherin 20; CDH7L3; Cdh7	18q21.33
CDH22	13251	64405	Cadherin 22; DJ998H6.1; C20orf25	20q13.12
CDH23	13733	64072	Cadherin 23; CDHR23; Cadherin-Related Family Member 23	10q22.1
CDH24	14265	64403	Cadherin 24; CDHH11L; Cadherin 24, Type 2	14q11.2
CDH26	15902	60437	Cadherin 26; VR20; Cadherin-Like Protein VR20	20q13.33

It is widely accepted that the epithelial-to-mesenchymal transition (EMT) of epithelial cells results in strong cell-cell adhesion and more invasive features [18]. The EMT is essential for this phenomenon and is considered a promoter of metastasis, and metastatic processes associated with mesenchymal features are similar among various cancers such as advanced breast cancer. The EMT has also received a lot of interest in cancer research and is thought to be an important step in metastases [19, 20]. As a result, finding new molecules that can inhibit this mechanism is an important subject of scientific study. A feature of the EMT is in part a result of downregulation of *CDH1* and parallel upregulation of other cadherins like *CDH2*, which plays an essential role during early invasion and metastasis [21]. Loss of *CDH1* alone might be insufficient to induce the EMT [22]. Instead, *CDH1* expression was observed in invasive lobular carcinomas (ILCs) and invasive ductal carcinomas (IDCs) [23]. Other cadherins and molecules such as β-catenin, which forms an important membrane complex, are often detached from the cell membrane and are translocated to the nucleus to induce EMT signaling events [24–26].

Previous studies reported the roles of cadherins in breast cancer. However, interactions and pathways among all *CDH* family members and related molecules in tumorigenesis are still unclear, and challenges remain in discovering suitable biomarkers for precision treatment and detection.

**Figure 1:**
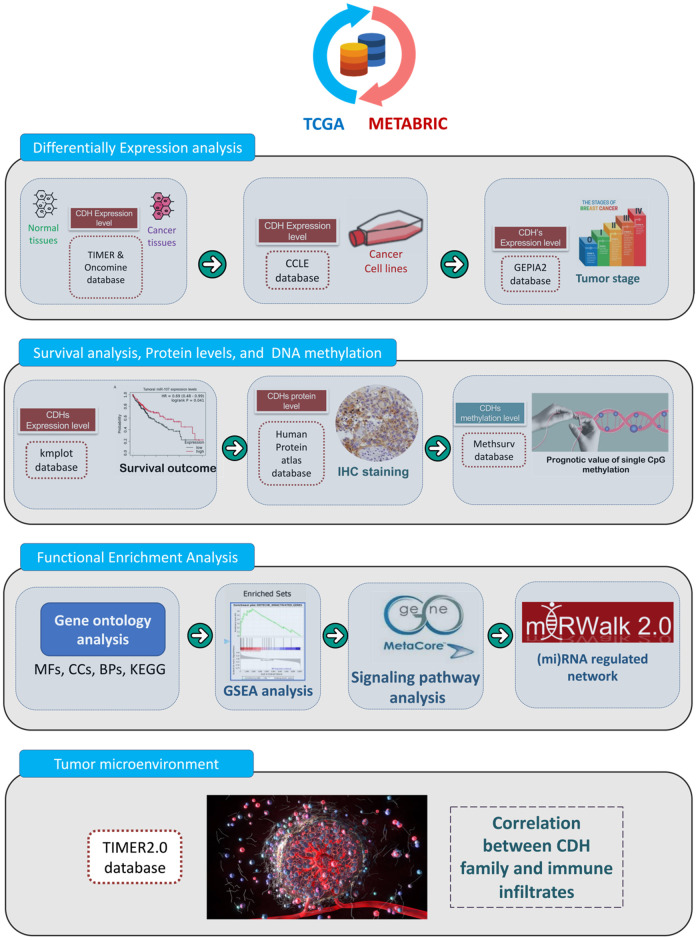
**Flowchart of the study design and analytical steps in the present study.** Gene data were retrieved from TCGA and METABRIC databases. To select targeted genes among the cadherin (CDH) family, we observed results of simultaneous higher expression levels in cancer cells than normal tissues and poorer prognoses in breast cancer patients. Afterwards, through four steps of a “differential expression analysis”, “survival analysis, protein levels, and DNA methylation”, “functional enrichment analysis”, and “tumor microenvironment”, a comprehensive analysis was conducted with the following databases and analytical methods. TCGA, The Cancer Genome Atlas; METABRIC, Molecular Taxonomy of Breast Cancer International Consortium; TIMER, Tumor IMmune Estimation Resource; CCLE, Cancer Cell Line Encyclopedia; GEPIA2, Gene Expression Profiling Interactive Analysis 2; KM, Kaplan-Meier; IHC, immunohistochemistry; BPs, biological processes; MFs, molecular functions; CCs, cellular components; KEGG, Kyoto Encyclopedia of Genes and Genomes; GSEA, Gene Set Enrichment Analysis; miRNA, micro-RNA.

The present study is the first study to perform a bioinformatics analysis of the entire *CDH* family in patients with breast cancer by analyzing several large online databases. A flowchart depicting the investigative strategies we utilized in this study, including expression levels, clinical survival, and functional enrichment analyses, of *CDH* family members in breast cancer is presented in Figure 1. First, original data were retrieved from the Molecular Taxonomy of Breast Cancer International Consortium (METABRIC) and The Cancer Genome Atlas (TCGA) databases. Second, differential expression levels were analyzed using the Oncomine and Tumor Immune Estimation Resource (TIMER) databases. Third, Kaplan-Meier (KM) plots were utilized to reveal the significance of *CDH* family in the prognosis of breast cancer patients. Incorporating these results, we selected targeted genes due to higher expression levels and lower survival for further analysis. Then, the Cancer Cell Lines Encyclopedia (CCLE) and Gene Expression Profiling Interactive Analysis 2 (GEPIA2) databases were used to discover differences in expressions between breast cancer and normal tissues [27–31]. Afterwards, we used the MethSurv database to determine single CpG methylation expression patterns. In addition, we studied the gene potential thoroughly through a functional enrichment analysis and micro (mi)RNA-regulated networks, including biological processes (BPs), cellular components (CCs), molecular functions (MFs), signaling pathways, and potentially regulated miRNAs. Ultimately, we utilized the TIMER2.0 database to uncover correlations between *CDH* genes and immune cell markers in breast cancer. The flowchart is presented to offer insights into our comprehensive approach and possibly suggest a theoretical foundation for future research.

## MATERIALS AND METHODS

### Oncomine analysis

Oncomine (https://www.oncomine.com/) is an online database established to show information of gene expressions in major cancers compared to their respective normal samples [32]. In this study, individual expression levels of *CDH* family members in various cancers were obtained from the Oncomine database with *p*<0.05 and fold change (FC) defined as 1.5 [33–37].

### TIMER and GEPIA2 analyses

The TIMER database was utilized to identify complements or regulatory factors that are upregulated or downregulated in tumor samples compared to normal tissues. To analyze differences in gene expressions of each *CDH* family member between breast cancer and normal tissues, differentially expressed genes (DEGs) in breast invasive carcinoma (BRCA) in TCGA dataset were identified via TIMER. The threshold |log2[FC]| was set to 1, and the value of *q* was 0.05. GEPIA2 (http://gepia2.cancer-pku.cn/#index) is a web platform that contains RNA sequencing (RNA-Seq) expression data from 9736 tumors and 8587 normal samples from TCGA and GTEx projects [38]. An independent t-test was used to calculate p values, and p<0.05 was considered statistically significant; Pr(>F) < 0.05 was based on Student’s t-test [39–44].

### KM plotter survival analysis

The KM plotter (http://kmplot.com/analysis/) contains 54,000 genes associated with survival in 21 types of cancer [45], including breast cancer samples (*n*=7830), which can be analyzed to examine the effects of *CDH* gene family members on survival times of patients with breast cancer. Results are presented by plotting the survival curve and hazard ratios (HRs) with 95% confidence intervals (CIs) and log-rank *p* values [46]. To assess the prognosis of breast cancer patients, distant metastasis-free survival (DMFS) was applied to evaluate the survival of advanced breast cancer patients.

### Genetic alterations and protein expression analysis

The cBioPortal (http://www.cbioportal.org/) is an open platform providing large-scale visualization, analysis, and downloading of cancer genomic datasets for various types of cancer [47, 48]. Cancer genome profiles can be obtained by a portal query interface, allowing researchers to explore and compare genetic alterations across samples. This study used the cBioPortal to explore alterations, correlations, and networks of the *CDH* gene family. CDH family protein expressions were evaluated by the Human Protein Atlas (HPA) platform. HPA contains images of pathologic tissues labeled with antibodies in conjunction with 11,250 human proteins. Microarrays include sections from forty-six normal tissues and more than twenty types of human cancers [49–51]. This study used the HPA to obtain the intensities of labeled antibodies in pathologic malignant tissues. Bar charts represent the quantification of four classifications, “negative”, “weak”, “moderate”, and “strong”, of IHC staining intensities in breast cancer samples with different antibodies.

### Expression modules in breast cancer gene-expression analysis

“Breast cancer gene-expression miner” (bc-GenExMiner), which contains published annotated breast cancer transcriptomic data (DNA microarrays [*n*=11,359] and RNA-Seq [*n*=4421]), is a breast cancer-associated web portal (http://bcgenex.ico.unicancer.fr) that conducts several differential gene expression analyses. We obtained data from Affymetrix® median probe data. To evaluate the difference in a gene’s expression among different groups, Welch’s test was used. Moreover, Dunnett-Tukey-Kramer’s test was used for two-by-two comparisons (allowing determination of the significance levels but not giving a precise *p* value) when there were more than three different groups and Welch’s *p* value was significant. Variant corresponding clinical or pathological data is contained in bc-GenExMiner version 4.5, which stresses that the Expression Module can be utilized for both exploratory and validation purposes [52].

### Cancer cell line encyclopedia (CCLE) analysis

Over 1100 cell lines among 37 cancer types are contained in the CCLE database (https://portals.broadinstitute.org/ccle). The CCLE dataset provides extensive genomic data, computational analyses, and visualization [53]. For the present study, we used the CCLE dataset to investigate messenger (m)RNA expression levels of *CDH* family members to further verify their participation in cancer cell lines [54–57].

### DNA methylation analysis

The MethSurv (https://biit.cs.ut.ee/methsurv/) database was utilized to determine single CpG methylation expression patterns and establish a heatmap of the different DNA methylated regions [58]. DNA methylation values are presented as beta values (ranging from 0 to 1). We used the formula of *M* / (*M* + *U* + 100) to calculate each single methylation of CpG, where *M* and *U* respectively represent methylated and unmethylated intensity values.

### Functional enrichment and miRNA-regulated network analyses

The METABRIC and TCGA datasets in the cBioPortal database were accessed for functional enrichment analyses [59, 60]. There were two parts of the MetaCore analysis (https://portal.genego.com). The first part was to find overlapping genes coexpressed in the two datasets with Venny version 2.1. The second part was to uncover BPs, disease biomarker networks, breast neoplasm signaling pathways, and drug target networks [61–65]. Moreover, a gene ontology (GO) analysis was implemented to discover the functional significance of genes with BPs, MFs, CCs, and the Kyoto Encyclopedia of Genes and Genomes (KEGG) with *p* values of <0.05 indicating statistical significance [66–70]. Next, we used the median expression of targeted genes and then performed a differential analysis with an algorithm in the “DESeq2” package in R/Bioconductor. After the differential analysis, results were utilized for the gene set enrichment analysis (GSEA) with the Hallmark database [71–73]. Then, we used the “fgsea” packages in R Studio software to evaluate enriched pathways in transcriptional data by the GSEA, and online platform (http://www.bioinformatics.com.cn/) and used “SRplot” for visualization (http://www.bioinformatics.com.cn/srplot) [74, 75]. The level of statistical significance was presented via *p* values, and a normalized enrichment score (NES) reflected the rank of gene classes. In addition, the gene potential of the *CDH* family was conducted using the miRWalk database (http://mirwalk.umm.uni-heidelberg.de/) to investigate the regulatory potential of miRNAs and to analyze regulated pathways and networks by an Ingenuity Pathway Analysis (IPA) [76–79].

### Cox regression analysis in TIMER

The TIMER web server was accessed for a Cox regression analysis [80, 81]. We used the “Survival” module to explore the clinical significance of covariates in a multivariable Cox proportional hazard model. Clinical factors such as age, gender, ethnicity, and tumor stage and gene expression were covariates in the analysis. TIMER presents Cox regression results including hazard ratios (HRs) and statistical significance. For outputs of the Cox model, Surv(CancerType)~variables is the formula of the user-defined Cox regression model, which is fitted by the function coxph() from the R package ‘survival’. In the results, the coefficient reads as a regression coefficient. The 95% confidence intervals (CIs) are shown.

### Data availability

The present study is based on open-source data. Users could download relevant data in public databases for research.

## RESULTS

### Differential expressions of *CDH* family members in breast cancer

**Figure 2:**
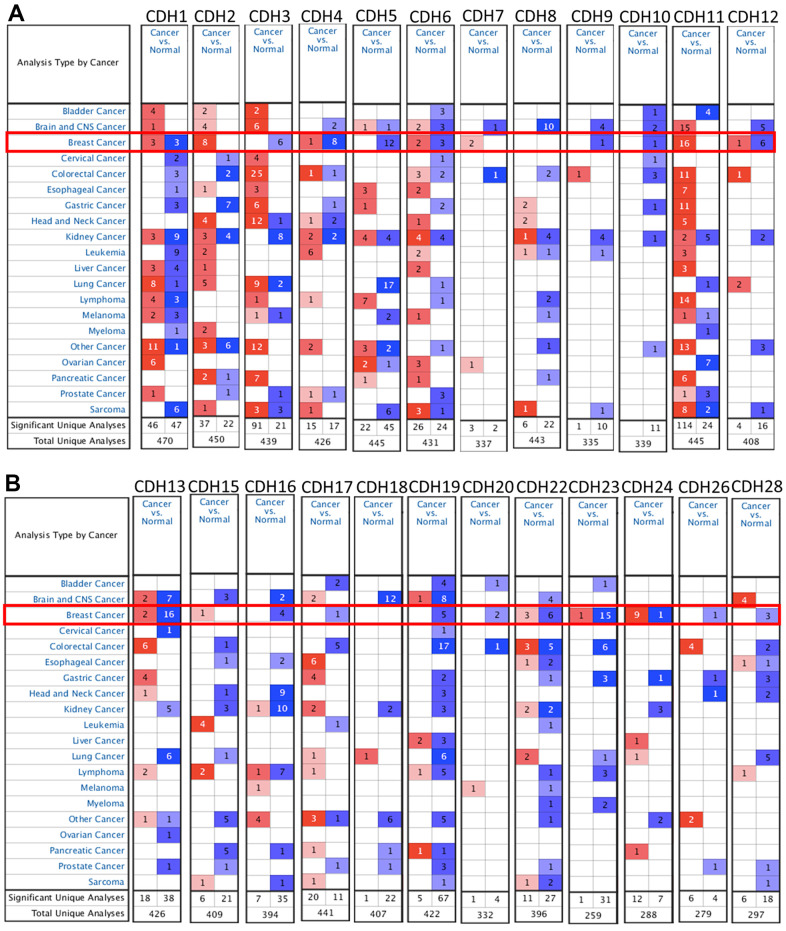
**mRNA transcription levels of cadherin (*CDH*) gene family members (ONCOMINE).** A red background with numbers indicates studies including expression levels of *CDH* family members corresponding to our selection standards (with *p* values <0.05, fold changes of >1.5, and the expressed gene rank in the top 10% as selection thresholds) in cancer tissues; blue (the same selection threshold) in normal tissues. The number for the significant unique analyses means that the queried genes significantly differed in these studies. The number for the total unique analyses means the total number of queried genes in these studies. (**A**) mRNA transcription levels of *CDH1/2/4/6/7/11/12* were overexpressed in breast cancer samples compared to normal tissues, while transcriptional levels of *CDH1/3/4/5/6/9/10/12* were downregulated compared to normal tissues. (**B**) mRNA transcription levels of *CDH13/15/22/23/24* were overexpressed in breast cancer samples compared to normal tissues, while transcriptional levels of *CDH13/16/17/19/20/22/23/24/26/28* were downregulated compared to normal tissues. Red indicates upregulation, and blue indicates downregulation compared to normal tissues.

**Figure 3:**
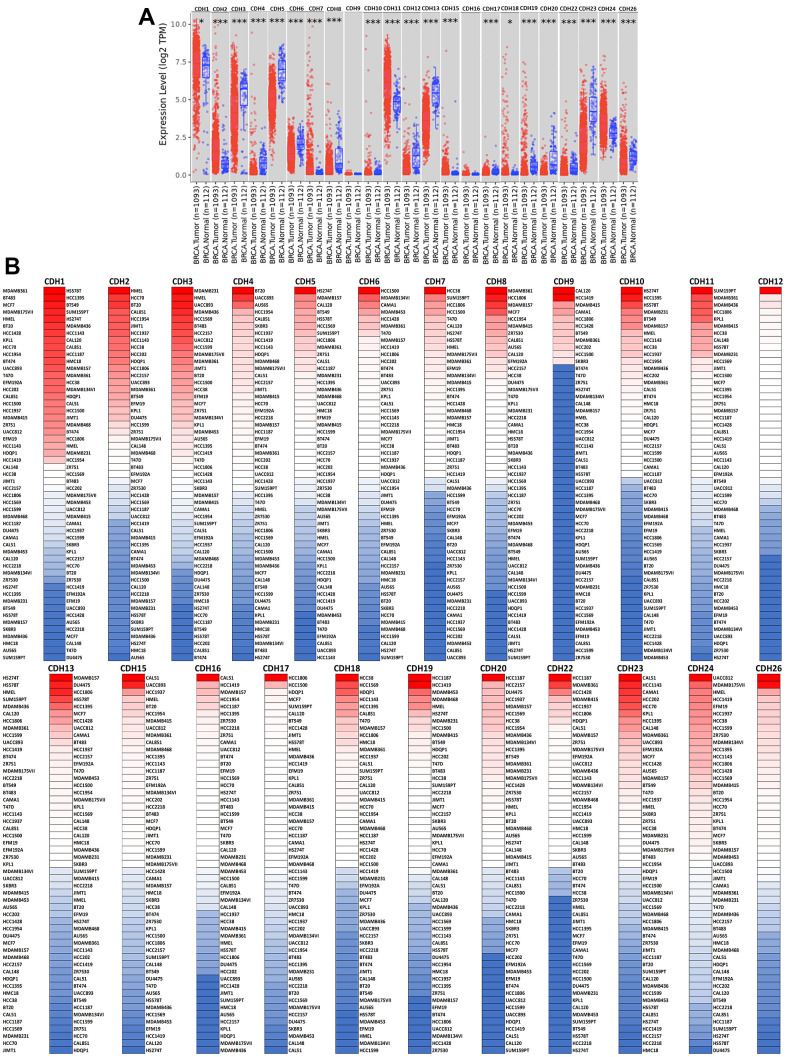
**Expression levels of cadherin (*CDH*) gene family members in breast invasive carcinoma (BRCA).** (**A**) In the TIMER database, a box plot shows transcripts of *CDH* gene family members in normal and breast cancer tissues. The Wilcoxon test was used to determine statistical significance; * *p*<0.05, *** *p*<0.001. (**B**) Expression levels of *CDH* gene family members in breast cancer cell lines are represented by a heatmap (CCLE). We used data from the CCLE database to generate mRNA expression values, which were then ranked. In CCLE, red denotes overexpression (top column), and blue denotes under-expression (bottom column).

To understand differences in expressions between breast cancer and normal tissues, all 24 *CDH* family members were investigated in the Oncomine database (Figure 2A, 2B). Findings of this database revealed that at the transcriptional level, *CDH1/2/4/6/7/11/12/13/15/22/23/24* were overexpressed in breast cancer samples compared to normal tissues, while transcriptional levels of *CDH1/3/5/8/9/10/16/17/18/19/20/26/28* were downregulated compared to normal tissues. In addition, complement expressions were explored across TCGA database via the TIMER database (Figure 3A). We investigated expression levels of *CDH* family members in breast cancer cell lines using the CCLE database as well (Figure 3B). Results revealed that expression levels were upregulated or downregulated in BRCA samples compared to non-tumor samples. Compared to normal tissues, expression levels of *CDH2/3/4/5/6/7/8/10/11/12/13/15/17/19/20/22/23/24/26* were significantly higher in BRCA tissues. In contrast, expression levels of *CDH1/18* were significantly lower in BRCA tissues. The molecular subtypes of cell lines are also shown in Supplementary Figure 1. *CDH1* and *CDH7* were highly expressed in luminal A cell lines; *CDH5* was mostly expressed in human epidermal growth factor receptor-2 (HER2) cell lines; *CDH2/3/4/6/11/12/13/15/18/19/22/23* showed high expressions in multiple triple-negative breast cancer cell lines; and other *CDH* genes showed no specific expressions in molecular subtypes of breast cancer cell lines.

### Prognostic analysis of the *CDH* family via Kaplan-Meier analyses

**Figure 4:**
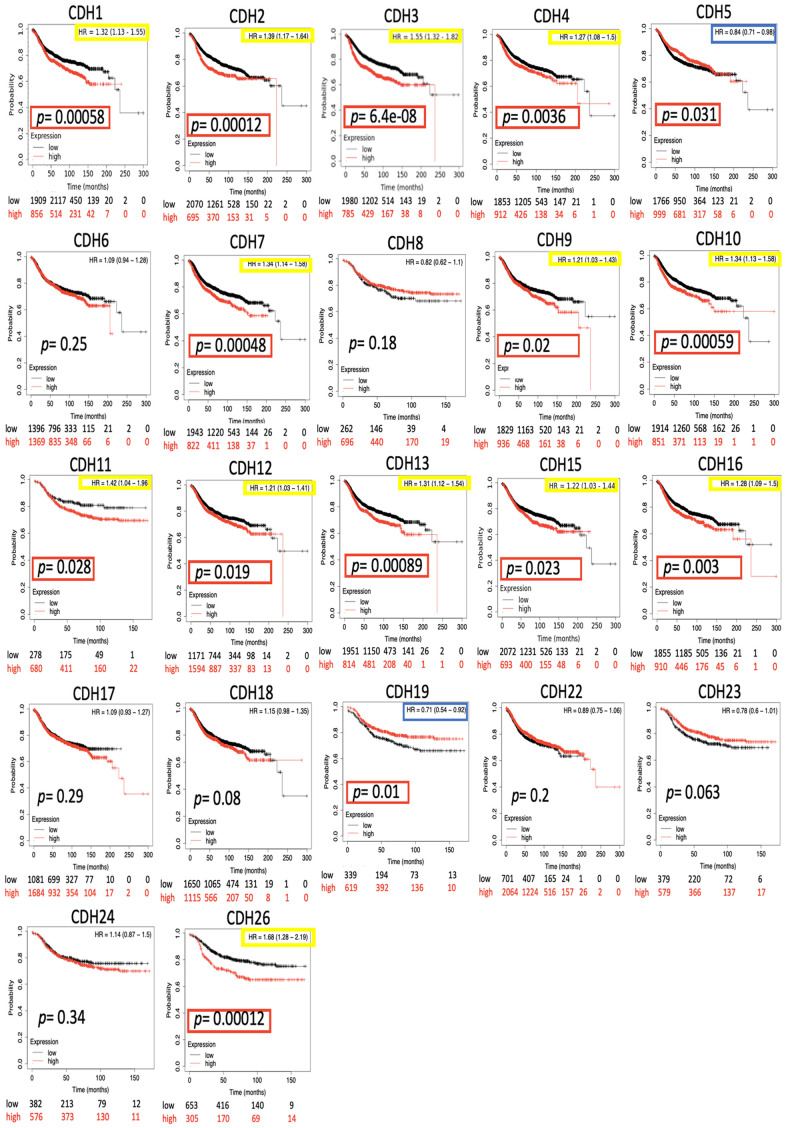
**Kaplan-Meier (KM) analysis of cadherin (*CDH*) family genes in the distant metastasis-free survival (DMFS) of breast cancer patients.** The hazard ratio (HR) represents a prognostic value of breast cancer patients. Log[rank *p*] was utilized to find out the level of prognostic significance of breast cancer patients. In addition, log[rank *p*] of <0.05 was considered a significant difference in the prognosis of breast cancer patients. High expressions of *CDH1/2/3/4/5/7/9/10/11/12/13/15/16/19/26* were significant compared to low expressions, which are highlighted with *p* values in red boxes. The HRs of *CDH1/2/3/4/7/9/10/11/12/13/15/16/26* were significantly higher, which are marked in yellow boxes, indicating poor prognostic outcomes in breast cancer. In contrast, the HRs of *CDH5/19* were significantly lower, which were marked in blue boxes, indicating better prognostic outcomes in breast cancer.

The impact of the entire *CDH* family on breast cancer survival was evaluated through the KM plotter database. Distant metastasis-free survival (DMFS) was analyzed due to its significance in clinical prognosis of advanced breast cancer. Results demonstrated that most *CDH* family genes were associated with the prognosis of BRCA patients including *CDH1/2/3/4/5/7/9/10/11/12/13/15/16/19/26* (Figure 4 and Table 2). High expression levels of *CDH1* (HR=1.32, 95% CI=1.13~1.55, *p*=0.0058), *CDH2* (HR=1.39, 95% CI=1.17~1.64, *p*=0.00012), *CDH3* (HR=1.55, 95% CI=1.32~1.82, *p*=6.4e-8), *CDH4* (HR=1.27, 95% CI=1.08~1.5, *p*=0.0036), *CDH7* (HR=1.34, 95% CI=1.14~1.58, *p*=0.00048), *CDH9* (HR=1.21, 95% CI=1.03~1.43, *p*=0.02), *CDH10* (HR=1.34, 95% CI=1.13~1.58, *p*=0.00059), *CDH11* (HR=1.42, 95% CI=1.04~1.96, *p*=0.028), *CDH12* (HR=1.21, 95% CI=1.03~1.41, *p*=0.019), *CDH13* (HR=1.31, 95% CI=1.12~1.54, *p*=0.00089), *CDH15* (HR=1.22, 95% CI=1.03~1.44, *p*=0.023), *CDH16* (HR=1.28, 95% CI=1.09~1.5, *p*=0.003), and *CDH26* (HR=1.68, 95% CI=1.28~2.19, *p*=0.00012) were correlated with poorer DMFS in BRCA patients. On the other hand, high expressions of *CDH5* (HR=0.84, 95% CI=0.71~0.98, *p*=0.031) and *CDH19* (HR=0.71, 95% CI =0.54~0.92, *p*=0.01) were associated with a good prognosis in BRCA patients. Other family members in the *CDH* family showed negative results.

**Table 2:** Kaplan-Meier analysis of CDH family genes in distant metastasis-free survival (DMFS) in breast cancer.

**Gene**	**HR (95% CI)**	***p* value**	**Gene**	**HR (95% CI)**	***p* value**
*CDH1*	1.32 (1.13~1.55)	0.0058	*CDH12*	1.21 (1.03~1.41)	0.019
*CDH2*	1.39 (1.17~1.64)	0.00012	*CDH13*	1.31 (1.12~1.54)	0.00089
*CDH3*	1.55 (1.32~1.82)	6.4e-08	*CDH15*	1.22 (1.03~1.44)	0.023
*CDH4*	1.27 (1.08~1.5)	0.0036	*CDH16*	1.28 (1.09~1.5)	0.003
*CDH5*	0.84 (0.71~0.98)	0.031	*CDH17*	1.09 (0.93~1.27)	0.29
*CDH6*	1.09 (0.94~1.28)	0.25	*CDH18*	1.15 (0.98~1.35)	0.08
*CDH7*	1.34 (1.14~1.58)	0.00048	*CDH19*	0.71 (0.54~0.92)	0.01
*CDH8*	0.82 (0.62~1.1)	0.18	*CDH22*	0.89 (0.75~1.06)	0.2
*CDH9*	1.21 (1.03~1.43)	0.02	*CDH23*	0.78 (0.6~1.01)	0.063
*CDH10*	1.34 (1.13~1.58)	0.00059	*CDH24*	1.14 (0.87~1.5)	0.34
*CDH11*	1.42 (1.04~1.96)	0.028	*CDH26*	1.68 (1.28~2.19)	0.00012

High expressions of *CDH1/2/3/4/5/7/9/10/11/12/13/15/16/19/26* were significant compared to low expressions.

A univariate Cox regression analysis was conducted to validate our results from clinical breast cancer patients, data of which were obtained from the TIMER database. The univariate Cox regression demonstrated that high levels of *CDH13* were an independent risk factor for poor overall survival (OS) (Supplementary Table 1A) in breast cancer patients. In addition, subtypes of breast cancer, including luminal, HER2, and basal, were analyzed. The luminal subtype showed no significance among *CDH*s (Supplementary Table 1B). *CDH12* was a significant risk factor for poor OS in the HER2 subtype (Supplementary Table 1C). *CDH11* and *CDH12* were significant risk factors for poor OS in the basal subtype (Supplementary Table 1D).

To further understand correlations of expression levels of *CDH* family members in breast cancer, some clinical and pathological factors were analyzed in specific genes among the *CDH* family. Among all *CDH* family members, *CDH1/2/3/4/7/9/10/11/12/13/15/16/26* were significantly positively associated with a lower DMFS (Figure 4), and *CDH1/2/4/6/7/11/12/13/15/22/23/24* mRNA expression levels were higher in breast cancer than in normal tissues in the Oncomine database (Figure 2A, 2B). Results demonstrated that the eight *CDH1/2/4/7/11/12/13/15* genes simultaneously expressed significance in the gene database and clinical survival analysis. Therefore, in this study, these eight specific genes were further analyzed with an extensive database, clinical factors, and bioinformatics tools and were demonstrated to be potential biomarkers for breast cancer.

### Correlations of *CDH1, CDH2, CDH4, CDH7, CDH11, CDH12, CDH13,* and *CDH15* expressions with prognosis and different clinical and pathological factors

**Figure 5:**
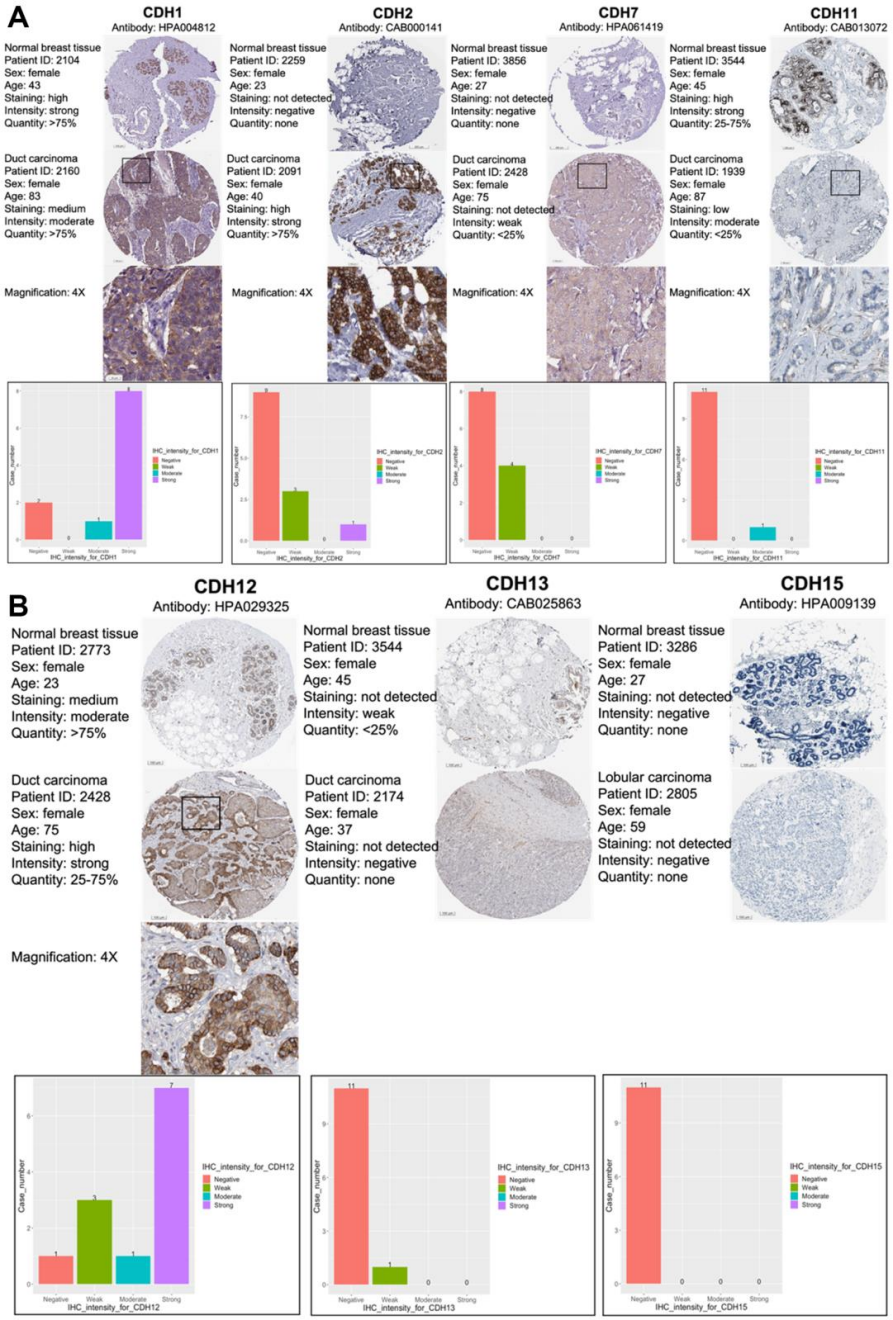
**Protein expression levels of members of cadherin (*CDH*) family genes in all clinical breast cancer specimens from the Human Protein Atlas (HPA).** (**A**) Images of immunohistochemistry (IHC) of *CDH1/2/7/11* show their staining intensities. IHC images and patients’ information were obtained from the HPA. Normal and tumor samples are listed, and bar charts represent the quantification of IHC staining in breast cancer samples. There were strong intensities of *CDH1* and *CDH2* in breast cancer samples. (**B**) IHC images of *CDH12/13/15* show their staining intensities. IHC images and patient information were obtained from the HPA. Normal and tumor samples are listed, and bar charts present quantification of IHC staining in breast cancer samples. There were strong intensities of *CDH12* in breast cancer samples.

As *CDH1/2/4/7/11/12/13/15* were positive in terms of both gene expressions and with the KM survival analysis, immunohistochemical (IHC) patterns from the HPA were utilized to validate clinical applications by pathology (Figure 5A, 5B). *CDH1, CDH2*, and *CDH12* exhibited strong intensities in cell nuclei in breast cancer samples. Otherwise, other members of the *CDH* family showed negative or weak intensities in pathological samples. The relative staining intensities of *CDH1* were negative (two cases), moderate (one case), and strong (eight cases) in breast cancer samples. *CDH2* staining intensities were negative (nine cases), weak (three cases), and strong (one case) in breast cancer samples. *CDH12* staining intensities were negative (one case), weak (three cases), moderate (one case), and strong (seven cases) in breast cancer samples. There was no IHC pattern for *CDH4* obtained from the HPA. Other staining intensities of *CDH* family members are shown in Figure 5A, 5B. CDH staining expressions, magnification in 4x, were displayed among CDH family except CDH13 and CDH15 due to negative intensities.

**Figure 6:**
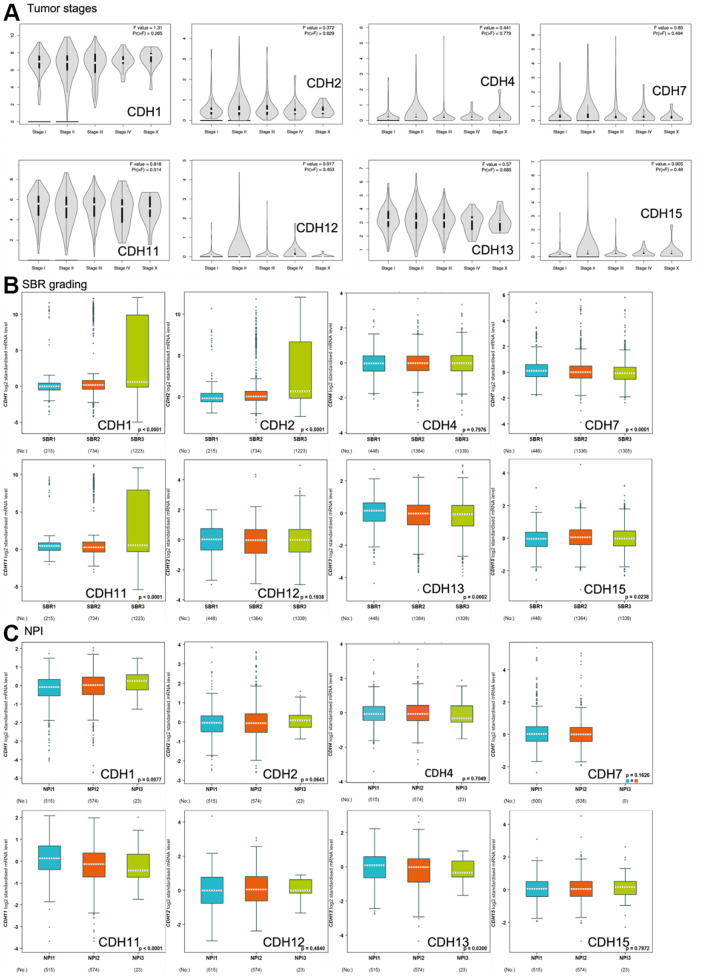
**Expression of cadherin (*CDH*) family genes in subgroups of breast cancer patients.** (**A**) Gene expression analysis among the stages of *CDH* genes in a breast cancer (GEPIA2) database. The violin plot displayed comparisons of *CDH* genes expressions from TCGA dataset of breast cancer. An independent *t*-test was utilized for *p* values; *p*<0.05 meant statistically significant; Pr(>F) <0.05 was based on Student’s *t*-test. (**B**) Scarff-Bloom-Richardson (SBR) grading of *CDH* family genes. Associations between *CDH1/2/4/7/11/12/13/15* and SBR grading were analyzed via the bc-GenExMiner dataset. (**C**) The Nottingham prognostic index (NPI) of *CDH* family genes. Associations between *CDH1/2/4/7/11/12/13/15* and NPI values were analyzed via the bc-GenExMiner dataset.

Correlations of expression levels of *CDH1/2/4/7/11/12/13/15* with pathological stages of breast cancer are shown *in violin* plots in Figure 6A. mRNA levels of *CDH1/11/13* were relatively high in breast cancer patients classified as stage IV with metastasis but without statistical significance.

Scarff-Bloom-Richardson (SBR) grading is a clinical prognostic predictor associated with cell proliferation and an indicator of the response to chemotherapy (Figure 6B). A determination of an association between the SBR grade and responsiveness would be clinically useful [82]. SBR1 indicates good differentiation, SBR2 moderate differentiation, and SBR3 poor differentiation. Figure 6B demonstrates that *CDH1, CDH2*, and *CDH11* with the poorest prognoses were assigned to grade SBR3.

The Nottingham prognostic index (NPI) is used to predict a prognosis after breast cancer surgery, and is calculated by three pathological factors: the tumor size, the number of involved lymph nodes, and the tumor grade (Figure 6C). Values are used to define three subsets of patients with different survival chances of breast cancer: 1) good prognosis, comprising 29% of patients with an 80% chance of 15-year survival; 2) moderate prognosis, 54% of patients with a 42% chance of 15-year survival; and 3) poor prognosis, 17% of patients with a 13% chance of 15-year survival [83]. The NPI can also be used to evaluate the effect of adjuvant treatment like chemotherapy or radiotherapy. Figure 6C demonstrates that only *CDH1* expression was correlated with higher NPI values, indicating poor prognoses in patients with *CDH1* gene expression. Otherwise, *CDH11* and *CDH13* expressions demonstrated lower NPI values with better prognoses. Other *CDH* family members showed no significance.

Other clinical predictors were also analyzed in terms of *CDH* family gene expressions in breast cancer (Supplementary Figure 2 in Supplementary Materials). Estrogen receptor (ER)/progesterone receptor (PR)-positive samples showed a high probability of positive effects of hormone therapy such as with tamoxifen. HER2 samples corresponded to positive effects of targeted therapy with trastuzumab. Subtypes of breast cancer including basal-like, HER2-E, luminal A, and luminal B were correlated with different pathological characteristics and clinical prognoses. Mutations of breast cancer gene-1 (*BRCA1*) and *BRCA2* were also analyzed with respect to *CDH* family gene expressions, which represent breast cancer oncogenes (Supplementary Figure 2D). Supplementary Figure 2A demonstrates that ER-/PR- expressions were correlated with higher expressions of *CDH2* and *CDH11*, indicating a poorer response to hormone therapy. Supplementary Figure 2B demonstrates that HER2-negative expression was found to be associated with *CDH7* and *CDH11*, suggesting a poorer response to targeted therapy. Supplementary Figure 2C demonstrates relationships of different subtypes of breast invasive carcinoma with *CDH* family gene expressions. *CDH1* was highly expressed by the HER2-E and luminal B types; *CDH2* was highly expressed by the HER2-E type; *CDH4* was highly expressed by the basal-like type; *CDH7* and *CDH11* were highly expressed by the luminal A type; *CDH12* and *CDH13* were highly expressed by the basal-like and luminal A types; and *CDH15* expression was significantly associated with no types.

### Gene mutation analysis of *CDH1/2/4/7/11/12/13/15* in breast cancer

**Figure 7:**
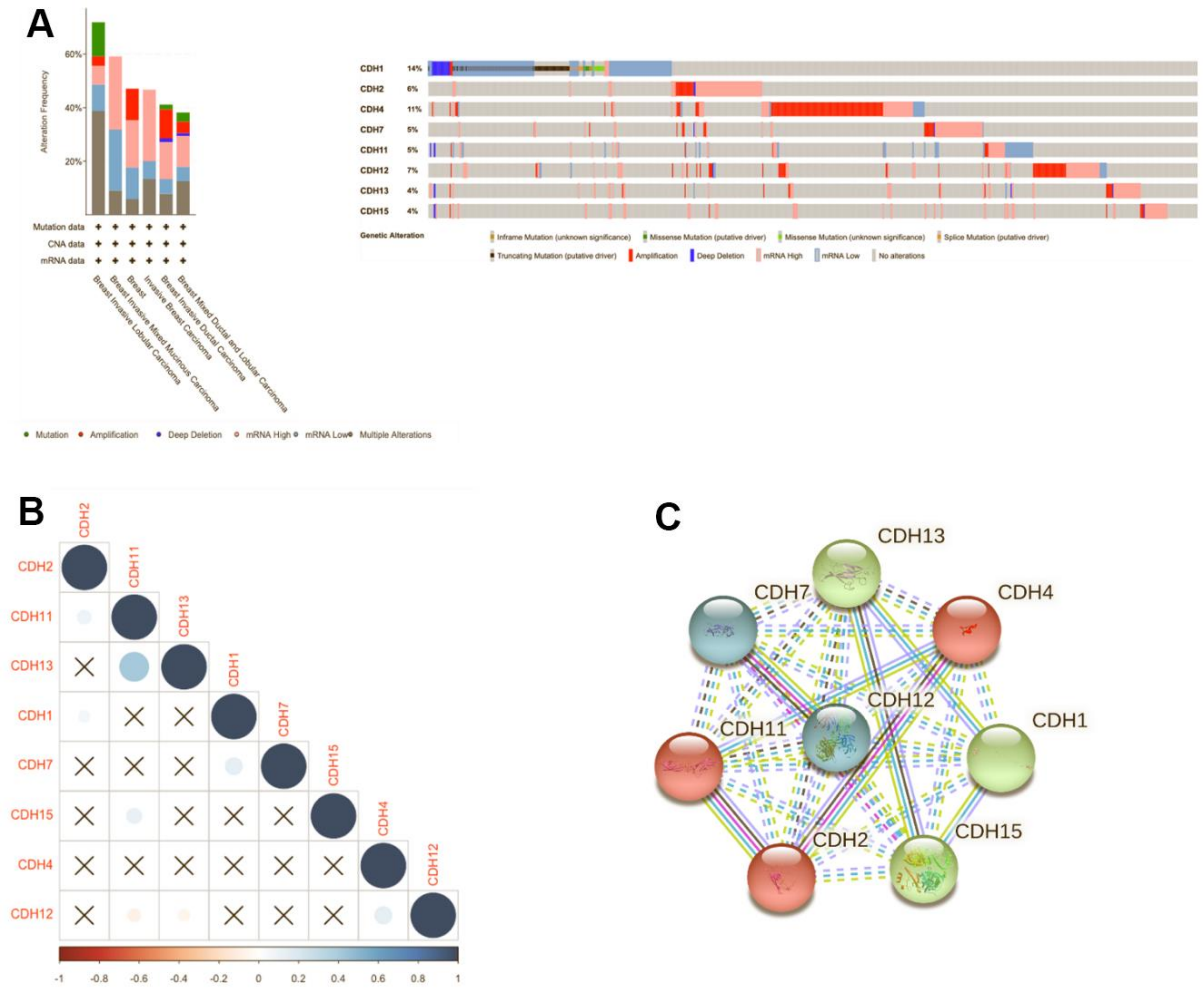
**Genomic alterations of differentially expressed cadherin 1 *(CDH1)/2/4/7/11/12/13/15* genes in breast cancer.** (**A**) The cBioPortal database was used to reveal levels of gene amplification, deep deletions, and associated nucleotide substitutions of the *CDH1/2/4/7/11/12/13/15* genes in breast cancer progression in the METABRIC dataset. (**B**) Correlation plot of the *CDH1/2/4/7/11/12/13/15* genes in breast cancer (cBioPortal) database. Insignificant correlation values were displayed by crosses; *p*<0.01 was considered statistically significant. (**C**) Protein-protein interactions (PPIs) of *CDH1/2/4/7/11/12/13/15* (STRING database). Highly interacting proteins were represented as hub protein nodes in the PPI network.

Genomic changes in the *CDH* family were analyzed via the cBioPortal database, which demonstrated changes in *CDH1* (14%), *CDH2* (6%), *CDH4* (11%), *CDH7* (5%), *CDH11* (5%), *CDH12* (7%), *CDH13* (4%), and *CDH15* (4%) (Figure 7A). Our results of mutated gene frequencies demonstrated that those of *CDH1* and *CDH4* were >10%. Altered genes at higher frequencies affect signaling pathways and cellular processes and can induce tumorigenesis based on previous studies [84–86]. *CDH1* showed more gene alterations of truncating mutations and deep deletions, and low mRNA expression, while in contrast, *CDH4* showed more amplifications and high mRNA expression. In the METABRIC dataset, *CDH1* acted more like a TSG, and *CDH4* acted like an oncogene in breast cancer. A previous study demonstrated that loss of E-cadherin was a key hallmark of ILCs [87]. In Giovanni et al. [86], *CDH1* was one of the most recurrently mutated genes in breast cancer. In mixed ILC-IDC samples, genetic alterations of ILC tumors were found at a frequency of 14%. Mutations targeting *CDH1* were mostly truncated mutations, and this result was similar to our mutation analysis.

We also used Pearson’s correlations to calculate correlations between *CDH* family members based on mRNA expressions (Figure 7B). *CDH11* was significantly positively correlated with *CDH13*. Other genes in the *CDH* family otherwise showed no relative correlations with each other (Figure 7B). In addition, a protein-protein interacting (PPI) network analysis of the *CDH* family was conducted via STRING at various transcription levels to investigate potential relationships. The STRING analysis revealed that linkages among *CDH* gene family members were complicated. Using a three-group k-means algorithm, it was found that the group consisting of *CDH1*, *CDH13*, and *CDH15* had a close relationship, and *CDH2*, *CDH4*, and *CDH11* comprised another related group. A third group consisted of *CDH7* and *CDH12* (Figure 7C).

### DNA methylation analysis of *CDH1/2/4/11/12/13* in breast cancer

We present a heatmap of DNA methylated locations of *CDH1/2/4/11/12/13* in breast cancer in Supplementary Figure 3 in “Supplementary Materials”. In total, 18 methylated CpG sites were determined for *CDH1*, with six CpG sites presenting high expressions. Among them, cg26508465 and cg09220040 showed the highest levels of DNA methylation. In total, 20 methylated CpG sites of CDH2 were determined with six CpG sites presenting high expressions. Among them, cg24776465 showed the highest level of DNA methylation. In total, there were 26 methylated CpG sites of *CDH11*, with 19 CpG sites presenting high expressions. Among them, cg02724025 showed the highest level of DNA methylation. Over half of the CpG sites of *CDH11* presented high levels of methylation and relevance to breast cancer. These results provide a potential mechanism by which *CDH11* can serve as an oncogene for breast cancer.

### Regulated networks of *CDH1/2/4/7/11/12/13/15* in breast cancer

**Figure 8:**
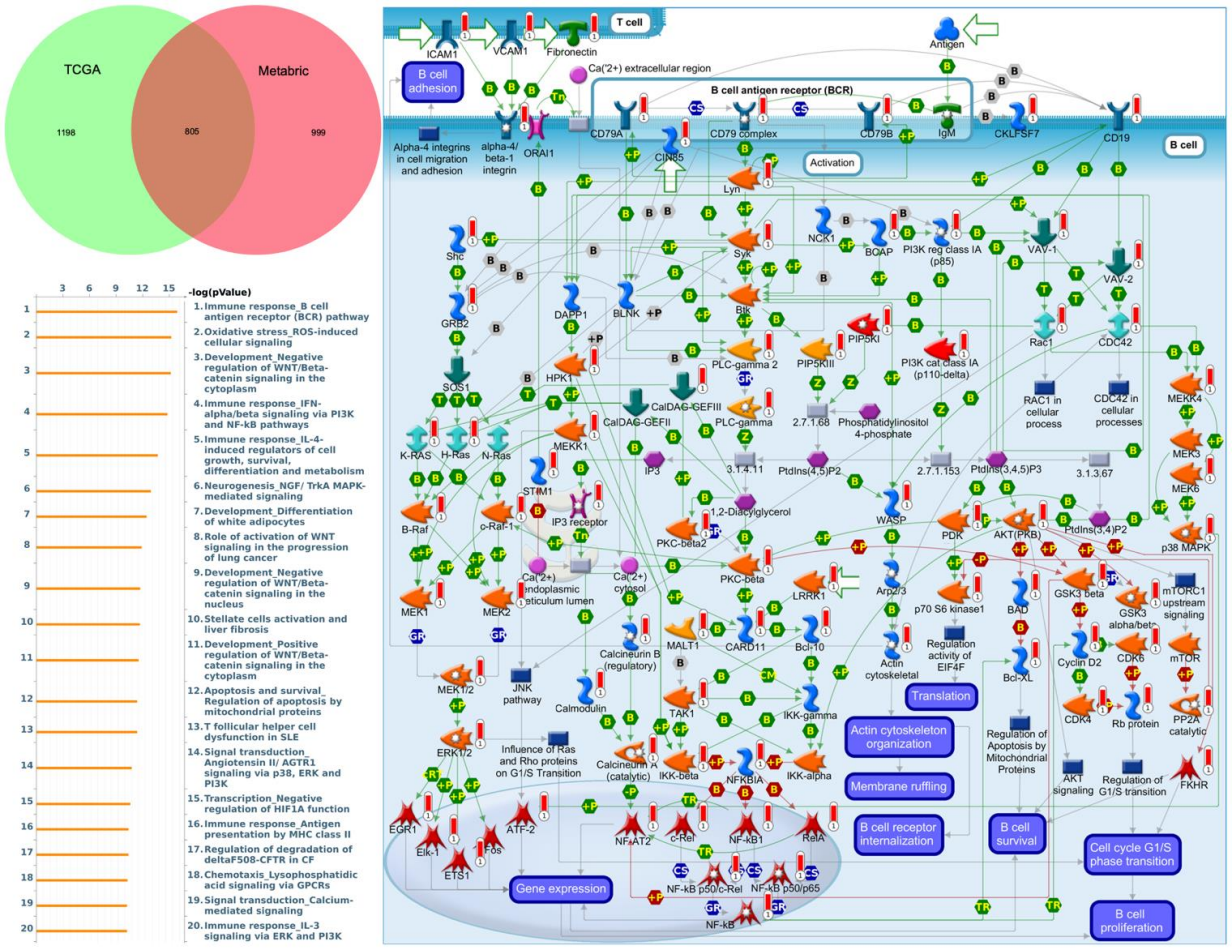
**MetaCore enrichment pathway analysis of genes coexpressed with cadherin 1 (*CDH1*).** The top 10% of expressed genes coexpressed with *CDH1* from both Metabric (1804 genes) and TCGA (2003 genes) were extracted. Overlapping (805) genes were integrated to implement a pathway analysis, which formed a pathway list ordered by the -log *p* value. “Immune response B cell antigen receptor (BCR) pathway” was at the top of the pathway list when performing the “biological process” analysis. The figure demonstrates interactions between genes and proteins. Symbols represent proteins. Arrows depict protein interactions (green, activation; red, inhibition). Thermometer-like histograms indicate microarray gene expressions (blue, downregulated; red, upregulated).

To understand how DEG lists are linked to downstream CDH-regulated networks in various biological pathways and diseases, an enrichment analysis was performed using MetaCore software. After uploading genes coexpressed with *CDH1* from Metabric and TCGA databases into MetaCore, we found that numerous pathways and networks were related to cell cycle (Figure 8 and Supplementary Table 2 in Supplementary Materials) including “Immune response_B cell antigen receptor (BCR) pathway”, “Oxidative stress ROS-induced cellular signaling”, “Development_negative regulation of WNT/Beta-catenin signaling in the cytoplasm”, and “Immune response_IFN-alpha/beta signaling via PI3K and NF-κB pathways”.

Similar pathway analyses of *CDH2, CDH4, CDH7, CDH11, CDH12, CDH13,* and *CDH15* are displayed in “Supplementary Materials” (Supplementary Figures 4–9 and Supplementary Tables 3–9). Genes coexpressed with *CDH2* were correlated with “Cell adhesion_ECM remodeling” and “Cytoskeleton remodeling_Regulation of actin cytoskeleton organization by the kinase effectors of Rho GTPases” (Supplementary Figure 4). Genes coexpressed with *CDH4* were correlated with “Protein folding and maturation POMC processing”, “Beta-catenin-dependent transcription regulation in colorectal cancer”, and “Cell adhesion_ECM remodeling” (Supplementary Figure 5). Genes coexpressed with *CDH7* were correlated with “Cell cycle_Chromosome condensation in prometaphase” and “Cell cycle_the metaphase checkpoint” (Supplementary Figure 6). Genes coexpressed with *CDH11* were correlated with “Cell adhesion_ECM remodeling”, “IL-1 beta-and endothelin-1-included fibroblast/myofibroblast migration and extracellular matrix production in asthmatic airways”, and “Development regulation of epithelial to mesenchymal transition (EMT)” (Supplementary Figure 7). Genes coexpressed with *CDH12* were correlated with “Cytoskeleton remodeling_Regulation of actin cytoskeleton organization by the kinase effectors of Rho GTPases” and “Development negative regulation of WNT/Beta catenin signaling in the cytoplasm”. Genes coexpressed with *CDH13* were correlated with “Development_Regulation of epithelial-to-mesenchymal transition (EMT)”, “Role of stellate cells in progression of pancreatic cancer”, and “Cell adhesion ECM remodeling” (Supplementary Figure 8). Genes coexpressed with *CDH15* were correlated with “Transcription_HIF-1 targets”, “Oxidative stress_ROS-induced cellular signaling”, and “Development_negative regulation of WNT/Beta catenin signaling in the cytoplasm”. In summary, genes coexpressed with *CDH11* and *CDH13* were both correlated with regulation of the EMT, while genes coexpressed with *CDH2*, *CDH4*, *CDH11*, and *CDH13* were all correlated with cell adhesion.

### Comprehensive results of *CDH1/2/4/11/12/13* in the functional enrichment analysis

### Gene ontology (GO) enrichment analysis

For comprehensive analysis, we obtained data from the METABRIC and TCGA datasets to acquire GO enrichment results including BPs, CCs, MFs, and KEGG (Supplementary Figures 9A–14A in Supplementary Materials). The BP analysis demonstrated that *CDH1* was correlated with T-cell activation; the CC analysis showed correlations with cell-cell junctions and vacuolar membranes; MFs revealed significant relationships with phospholipid binding and actin binding, while KEGG ontology indicated the role of the mitogen-activated protein kinase (MAPK) signaling pathway and cytokine-cytokine receptor interactions (Supplementary Figure 9A). For *CDH2*, BPs demonstrated correlations with positive regulation of catabolic processes; the CC analysis showed correlations with mitochondrial matrix and cell-cell junctions; MFs revealed significant relationships with actin binding and protein serine/threonine kinase activity, while KEGG ontology indicated the role of the phosphatidylinositol 3-kinase (PI3K)-Akt signaling pathway (Supplementary Figure 10A). For *CDH4*, BPs demonstrated correlations with proteasomal protein catabolic process; the CC analysis showed correlations with the mitochondrial inner membrane and mitochondrial matrix; MFs revealed significant relationships with actin binding and ion channel activity, while KEGG ontology indicated the role of pathways of multiple neurodegenerative diseases (Supplementary Figure 11A). For *CDH11*, BPs demonstrated correlations with non-coding (nc)RNA metabolic processes; the CC analysis showed correlations with the mitochondrial inner membrane and mitochondrial matrix; MFs revealed significant relationships with transcription coregulator activity and actin binding, while KEGG ontology indicated the role of pathways of multiple neurodegenerative diseases (Supplementary Figure 12A). For *CDH12*, BPs demonstrated correlations with positive regulation of catabolic processes; the CC analysis showed correlations with cell-cell junctions and the mitochondrial matrix; MFs revealed significant relationships with phospholipid binding and actin binding, while KEGG ontology indicated the role of pathways of multiple neurodegenerative diseases (Supplementary Figure 13A). For *CDH13*, BPs demonstrated correlations with positive regulation of catabolic processes; the CC analysis showed correlations with cell-cell junctions and the mitochondrial matrix; MFs revealed significant relationships with transcription coregulator activity, while KEGG ontology indicated the role of neuroactive ligand-receptor interactions (Supplementary Figure 14A).

### High expression levels of CDH2/4/11/12 were related to the epithelial-mesenchymal transition (EMT) in the GSEA analysis

GSEA results indicated that the Hallmark pathway analysis of *CDH1* was significantly associated with protein secretion, estrogen response_early, and mammalian target of rapamycin C1 (mTORC1) signaling (Supplementary Figure 9B in Supplementary Materials). Yet the EMT revealed negative correlations with *CDH1*. The Hallmark pathway analysis of *CDH2* revealed that it was significantly associated with the EMT, mTORC1 signaling, the G_2_M checkpoint, and E2F targets (Supplementary Figure 10B). The Hallmark pathway analysis of *CDH4* showed that it was significantly associated with the EMT, myogenesis, and apical junctions (Supplementary Figure 11B). The Hallmark pathway analysis of *CDH11* indicated that it was significantly associated with the EMT, UV response_DN, coagulation, and angiogenesis (Supplementary Figure 12B). The Hallmark pathway analysis of *CDH12* revealed that it was significantly associated with the EMT, tumor necrosis factor (TNF)-α signaling via nuclear factor (NF)-κB, and UV response_DN (Supplementary Figure 13B). The Hallmark pathway analysis of *CDH13* showed that it was significantly associated with UV response_DN, DNA repair, adipogenesis, and IL-2-signal transduction and activator of transcription 5 (STAT5) signaling (Supplementary Figure 14B). *CDH2/4/11/12* were all associated with EMT signaling in the GSEA and were seen to be important inflammation- and immune-related gene sets and cancer-related gene sets in tumor metastasis.

### Micro-(mi)RNA-regulated network analysis of CDH1/2/4/11/12/13

We used the miRWalk database to identify associations with *CDH1/2/4/11/12/13*, and network regulation was analyzed by an IPA. Analysis of miRNA-regulated networks with *CDHs* (Supplementary Figure 15) indicated that hsa-miR-219a-2-3p regulated *CDH1* and was thus associated with breast cancer development; hsa-miR-330-3p, has-miR-4429, and hsa-miR-199a-5p regulated *CDH2*; hsa-miR-4644, hsa-miR-211-5p, hsa-miR-520f-3p, hsa-miR-34e-5p, and hsa-miR-34a-5p regulated *CDH4*; hsa-miR-486-5p, hsa-miR-200c-3p, hsa-miR-200b-3p, hsa-miR-26a-5p, hsa-miR-140-5p, hsa-miR-128-3p, and hsa-miR-19a-3p regulated *CDH11*; and hsa-miR-30c-5p regulated *CDH13*. In a previous study, the miRNA hsa-miR-200 family was identified as being a definitive factor of the epithelial phenotype of malignant cells, which targeted the E-cadherin repressors, zinc finger E-box-binding homeobox 1 (ZEB1) and ZEB2 [88–90]. Meanwhile, hsa-miR-200 was identified as a repressor of the EMT and was downregulated in more-aggressive molecular subtypes of breast tumors such as HER2 and triple-negative [91]. Our results of miRNA-regulated networks that hsa-miR-200c-3p and hsa-miR-200b-3p regulated *CDH11* were consistent with previous studies.

### Levels of immune infiltration in breast cancer were related to *CDH1/2/4/7/11/12/13/15* Expressions

**Figure 9:**
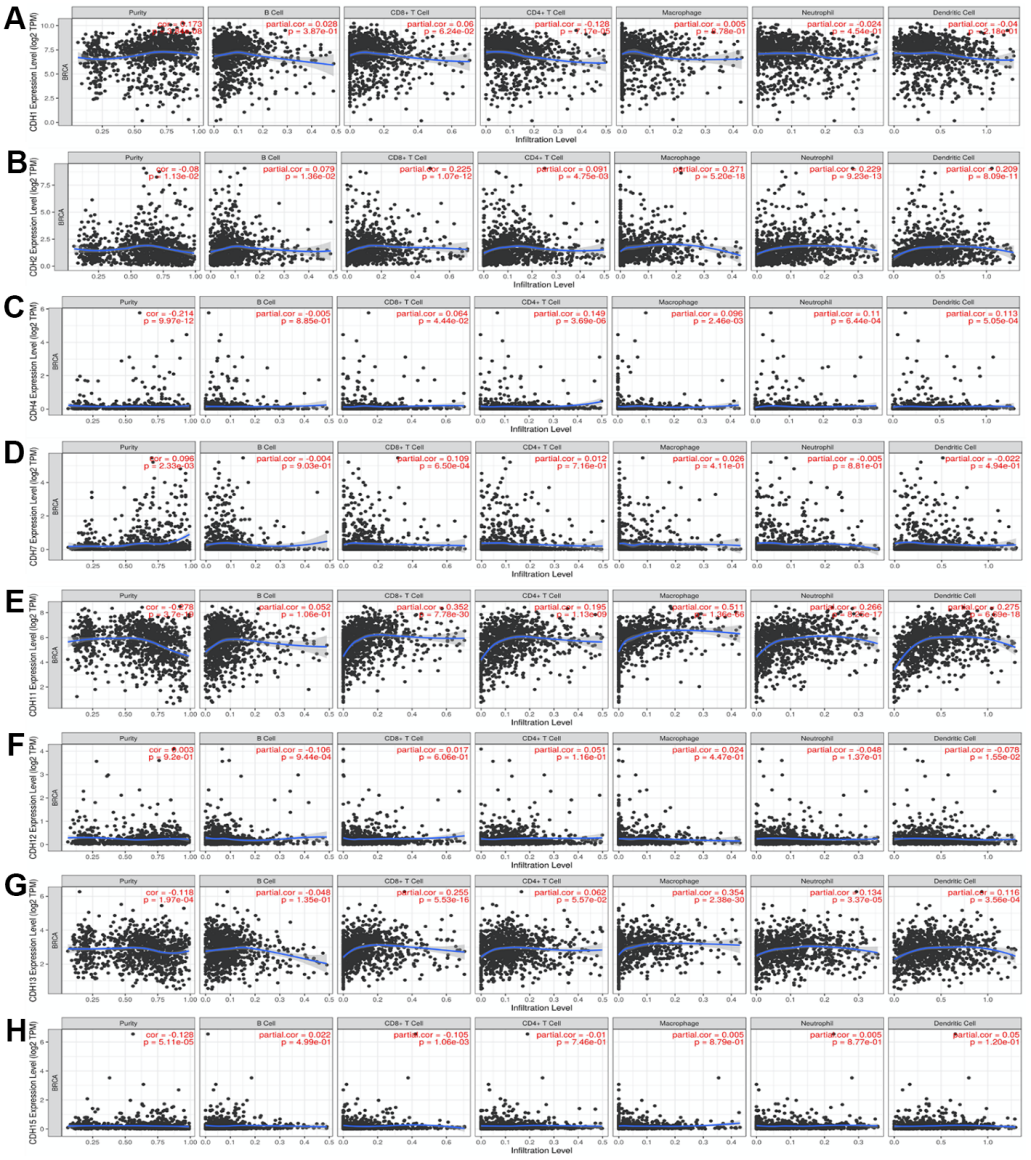
(**A**–**H**) Correlations between differentially expressed cadherin 1 (CDH1)/2/4/7/11/12/13/15 genes and immune cell infiltration in breast cancer. The figure showed that CDH1 (**A**); *CDH2* (**B**); *CDH4* (**C**); *CDH7* (**D**); *CDH11* (**E**); *CDH12* (**F**); *CDH13* (**G**); *CDH15* (**H**) gene expressions were associated with tumor purity and tumor-infiltrating immune cell markers, such as cluster of differentiation 8-positive (CD8^+^) T cells, B cells, CD4^+^ T cells, neutrophils, macrophages, and dendritic cells. Spearman correlations were applied to describe correlations between the CDH1/2/4/7/11/12/13/15 genes and the abovementioned immune cells (*p*<0.05 was accepted as statistically significant).

The TIMER database was utilized to investigate the immunological microenvironment. We identified correlations of immune infiltration levels with expressions of *CDH* gene family members in breast cancer (Figure 9). Results of the analysis showed significant correlations of *CDH1* with cluster of differentiation 4-positive (CD4^+^) T cells; *CDH2* with CD4^+^ T cells, macrophages, neutrophils, and dendritic cells (DCs); *CDH4* with CD8^+^ T cells, CD4^+^ T cells, macrophages, neutrophils, and DCs; *CDH7* with CD8^+^ T cells; *CDH11* with CD8^+^ T cells, CD4^+^ T cells, macrophages, neutrophils, and DCs; *CDH12* with B cells and DCs; *CDH13* with CD8^+^ T cells, CD4^+^ T cells, macrophages, neutrophils, and DCs; and *CDH15* with CD8^+^ T cells.

### Drug target network analysis of *CDH1/2/4/11/12/13*

After comprehensive research on *CDH* family members, we were curious about drug targets and related mechanisms of drug resistance. Hence, drug target networks of *CDH1/2/4/11/12/13* were analyzed by the MetaCore and MetaDrug system (Supplementary Figure 16 in Supplementary Materials). We found that “Signal transduction_c-myc, CREB1 signaling” was the top drug target of *CDH1*; “Cell adhesion_Fibrinogen, collagen signaling” was the top drug target of *CDH2*; “Metabolism_PPAR, RXR, VDR regulation of metabolism” was the top drug target of *CDH4*; “Cell adhesion_Fibrinogen, collagen signaling” was the top drug target of *CDH11*; “Transport_Potassium transport (core work 1)” was the top drug target of *CDH12*; and “Cell adhesion_Intergrin signaling” was the top drug target of *CDH13*.

## DISCUSSION

In previous studies, the *CDH* family was proven to be associated with invasiveness and metastasis [92–95]. The cadherin family as transmembrane glycoproteins mediate calcium-dependent cell-cell adhesion and regulates cell growth and differentiation. In the process of cell adhesion, cadherins act as essential factors to maintain stable homeostasis of tissue structures [96–98]. Once cell-cell adhesion is disturbed, adhesion-related pathways are subsequently interfered with. Disruption of cadherin signaling has determining influence on tumor progression and tumor immune responses [99–104].

In the present study, to determine whether *CDH* family members can serve as suitable biomarkers for breast cancer and pathways related to the EMT and metastasis, comprehensive integrative data mining was utilized, including gene expressions, survival analyses, clinical and pathological factors, immune infiltration, and enrichment pathway analyses. In the Oncomine, TIMER, and prognostic analyses, significantly high expression levels of *CDH1/2/4/7/11/12/13/15* were observed in breast cancer compared to normal tissue samples, and these were associated with poor DMFS outcomes. These results were confirmed by IHC staining in which *CDH1, CDH2*, and *CDH12* exhibited strong intensities. Furthermore, results of the bc-GenExMiner database demonstrated that increased *CDH4/12/13* expressions were associated with basal-like breast cancer, and increased *CDH1/2/11* expressions suggested a high SBR grade status in patients. Genetic mutations of *CDH1* and *CDH4* at frequencies of >10% showed higher possibilities of altering cell signaling pathways and promoting proliferation in malignancies. *CDH2, CDH4*, and *CDH11* had close relationships via the PPI network, and this was further confirmed by the MetaCore enrichment pathway analysis. These three *CDH* family genes, *CDH2, CDH4* and *CDH11*, and genes coexpressed with *CDH13* were correlated with the “Cell adhesion_ECM remodeling” process. *CDH11* and *CDH13* were also found to be closely related to *CDH1* due to its roles in regulating of the EMT. The enrichment pathway results suggested that in addition to *CDH1*, genes coexpressed with *CDH11* and *CDH13* were also correlated with “Development_Regulation of the epithelial-to-mesenchymal transition (EMT)”. These correlations of *CDH* family genes could lead to a better understanding of breast cancer development and metastasis.

The ability to infiltrate different tissues is a critical step in cancer because it defines the metastatic potential of tumor cells [105–108]. This capacity can be achieved by the EMT [109–111]. Previous studies reported that the EMT is featured by the loss of *CDH1* expression and the concomitant upregulation or de novo expression of *CDH2*, the so-called “cadherin switch”, which is associated with increased migration and invasiveness and thus poor prognoses [112–115]. The EMT causes disorganization of cell-cell adhesive junctions, thereby facilitating cancer metastasis. Irrespective of *CDH1* expression, the migratory and invasive capacities are present in tumor cells by *CDH2* expression. Therefore, *CDH2* seems to be the key factor in epithelial cancer metastasis and disease progression. Those studies demonstrated the key roles of *CDH2* in cancer metastasis, corresponding to our results with poor survival prognoses, strong intensities in pathological samples, and advanced SBR grading, indicating poor cell differentiation. Furthermore, we found that *CDH2/4/11* had similar signaling pathways with cell adhesion, which was further correlated with the EMT. In other words, high expression levels of *CDH2/4/11* are crucial for the EMT and cancer metastasis. To validate our results of positive correlations between *CDH* genes and the EMT, Pearson’s correlations were utilized to calculate correlations between *CDH1/2/4/7/11/12/13/15* and EMT-regulated genes such as *TWIST* and *SNAIL* based on mRNA expression levels (Supplementary Figure 17 in Supplementary Materials). Other EMT-core genes associated with cell adhesion and migration were obtained from a previous study [116]. *CDH11* displayed the greatest correlations with *COL3A1, COL1A1, COL5A1,* and *ADAM12* with Spearman’s rank correlation coefficients of >0.5 among these eight genes. *CDH13* showed mild positive correlations with *COL3A1, COL1A1, COL5A1, ADAM12, SNAI2, COL6A1,* and *TWIST2*. Conversely, traditional EMT markers, *CDH1* and *CDH2*, demonstrated nearly no correlations with these common EMT-regulated genes. *CDH1* only showed mild negative correlations with *TWIST2* and *SNAI3*. Other genes otherwise showed relatively no correlations with EMT-core genes. It was interesting to discover that *CDH11/13* demonstrated greater correlations with EMT-core genes rather than the traditional EMT-related cadherins, E-cadherin and N-cadherin, as mentioned in previous research [117, 118]. Our results supported the roles of *CDH11* in inducing the EMT, which corresponded to other research not only in cancer [119] but in other diseases including melasma [120] and pulmonary fibrosis [121].

To understand drug targets of *CDH* family genes, we implemented a drug target network analysis (Supplementary Figure 16). Since drug target network analyses of the *CDH2*, *CDH11*, and *CDH13* genes were all targeted to cell adhesion, we thus discussed the roles of cell adhesion in drug resistance. Cancer cells attaching to microenvironment components such as collagen type 1 (COL1) weakens the sensitivity of chemotherapeutic drugs like mitoxantrone, which is called cell adhesion-mediated drug resistance (CAM-DR) [122]. In consideration of the extensive presence of COL1 in mammary glands, breast cancer appears to have a high probability of presenting CAM-DR. The importance of COL1 is proven that patients with high-density breast tissues have higher risks of breast cancer [123–125] and poorer outcomes due to metastatic processes [126]. Regarding CAM-DR, the EMT plays a crucial part in drug resistance to breast cancer as well. The epithelial cell adhesion molecule (EpCAM) was implicated in tumor progression and drug resistance in breast cancer [127]. It was proven that EpCAM-knockdown resulted in upregulation of *CDH1* and attenuation of *CDH2* expression, which reversed the EMT. This process demonstrated that the EpCAM might possess the capability to induce the EMT in breast cancer to promote multidrug resistance. In addition, transcriptional silencing of *CDH1* was associated with the EMT in human breast cancer cells [128]. Previous research demonstrated that upregulation of E-cadherin by miR-200b and miR-200c via direct targeting the transcriptional repressors of E-cadherin, ZEB1 and ZEB2, inhibited the EMT [129]. In summary, *CDH1/2/11/13* were associated with the cell adhesion network on drug targets and were thus associated with important factors in drug resistance.

*CDH4* hypermethylation was significantly associated with increased risks for breast cancer in peripheral blood leukocyte DNA [130]. *CDH11* was also known as one of the mediators that interacted with malignant cells and normal cells and was detected in various cancers, especially in metastatic cancer cell lines [131–133]. In particular, *CDH11* was involved in the maintenance of high endogenous Rac activity and cytoskeletal reorganization in migratory breast cancer cells [134]. Moreover, because of the role of *CDH11* as an inducer of metastatic signaling, targeting *CDH11* triggered re-expression of the miR-335 tumor suppressor, which limited the *CDH11*-induced EMT. This phenomenon repressed cancer stem cell activities. *CDH11*-related pathways demonstrated the miR-335-mediated therapeutic value of anti-*CDH11* antibody treatment and provided a therapeutic option in patients with metastatic breast cancer. Downregulation of *CDH12* could inhibit the process of angiogenesis. Previous research implied that *CDH12* might be influential in colorectal tumor metastasis [135]. *CDH13* expression exhibited functions in cell adhesion and migration which were promoted by DNA polymerase beta (Pol β) by augmenting DNA demethylation of the *CDH13* promoter [136]. Abnormal methylation of *CDH13* promoter was observed in breast, colorectal, cervical and lung cancers, and chronic myeloid leukemia [137–139]. Those studies supported our results of the importance of *CDH4/11/12/13* in tumorigenesis. We supposed that high mRNA expression levels of *CDH4/11/12/13* were associated with breast cancer and poor survival.

As the tumor microenvironment plays important roles in tumorigenesis, we conducted an immune infiltration analysis in Figure 9. Previous studies also supported the associations between cadherin and immune pathways [140–142]. One of the most important pathways related to cadherin in immune responses is the Wnt pathway, which regulates cellular signaling by a canonical pathway with β-catenin [143]. β-Catenin plays a fundamental role in the cadherin protein complex, whose stabilization is crucial to activate the Wnt/β-catenin pathway. The WNT/β-catenin pathway mediates the self-renewal and relocation of cancer stem cells, promoting malignant progression and metastasis in breast cancer [144]. Our results of the enrichment pathway analysis were consistent with the importance of Wnt/β-catenin in breast cancer. Genes co-expressed with *CDH1/12/15* were correlated with the pathway of “Development negative regulation of WNT/Beta catenin signaling in the cytoplasm”. Induction of Wnt/β-catenin signaling was crucial in maintenance of stemness of memory CD8^+^ T cells by blocking T-cell differentiation [145]. Clinical responses to immune checkpoint inhibitors were correlated with tumors in the immune cell microenvironment [146, 147]. The Wnt/β-catenin pathway is considered to be a potential target for cancer treatment. In pancreatic cancer, effective immunotherapy is likely to require upregulation of *CDH1* expression [148]. The roles of cadherin and Wnt/β-catenin signaling in regulating immune cell infiltrations of the tumor microenvironment aroused interest in immunotherapy treatment.

This study performed a comprehensive and systematic review of the genetic expressions, prognostic values, mutation levels, immune infiltration, and enrichment pathways of the CDH family. CDH1/2/4/11/12/13 expressions are significantly increased in breast cancer and are associated with poor clinical prognoses of DMFS. We concluded that CDH1/2/4/11/12/13 may be crucial for breast cancer tumorigenesis, providing novel insights into developing detection biomarkers or targeted therapies for breast cancer. Nevertheless, evidence from clinical applications such as *in vitro* data or large patient cohorts should be produced to validate associations between *CDH1/2/4/11/12/13* and breast cancer.

## CONCLUSIONS

*CDH1/2/4/11/12/13* were overexpressed in breast cancer and were associated with poor prognoses in the distant metastasis-free survival analysis. Genes coexpressed with these *CDH* family members were correlated with regulation of the EMT and cell adhesion ECM remodeling, which were validated as playing critical roles in tumor metastasis. Although further evidence of clinical correlations for validation in the future should be determined to support our hypothesis, *CDH1/2/4/11/12/13* are expected to be potential biomarkers for breast cancer progression and metastasis.

## Supplementary Material

Supplementary Figures

Supplementary Tables

## References

[1] Gulmez A. Breast cancer after multiple myeloma treatment. Curr Probl Cancer. 2019; 43:100463. 10.1016/j.currproblcancer.2019.01.004 30738577

[2] Bray F, Ferlay J, Soerjomataram I, Siegel RL, Torre LA, Jemal A. Global cancer statistics 2018: GLOBOCAN estimates of incidence and mortality worldwide for 36 cancers in 185 countries. CA Cancer J Clin. 2018; 68:394–424. 10.3322/caac.21492 30207593

[3] Falzone L, Grimaldi M, Celentano E, Augustin LS, Libra M. Identification of Modulated MicroRNAs Associated with Breast Cancer, Diet, and Physical Activity. Cancers (Basel). 2020; 12:2555. 10.3390/cancers12092555 32911851 PMC7564431

[4] Wu M, Li Q, Wang H. Identification of Novel Biomarkers Associated With the Prognosis and Potential Pathogenesis of Breast Cancer via Integrated Bioinformatics Analysis. Technol Cancer Res Treat. 2021. 10.1177/1533033821992081 33550915 PMC7876582

[5] Pan Y, Liu G, Yuan Y, Zhao J, Yang Y, Li Y. Analysis of differential gene expression profile identifies novel biomarkers for breast cancer. Oncotarget. 2017; 8:114613–25. 10.18632/oncotarget.23061 29383106 PMC5777718

[6] Li CJ, Chen HM, Lai JC. Diagnostic, Prognostic, and Predictive Biomarkers in Breast Cancer. J Oncol. 2020; 2020:1835691. 10.1155/2020/1835691 32256579 PMC7091542

[7] Paul A, Paul S. The breast cancer susceptibility genes (BRCA) in breast and ovarian cancers. Front Biosci (Landmark Ed). 2014; 19:605–18. 10.2741/4230 24389207 PMC4307936

[8] van de Vijver MJ, He YD, van’t Veer LJ, Dai H, Hart AA, Voskuil DW, Schreiber GJ, Peterse JL, Roberts C, Marton MJ, Parrish M, Atsma D, Witteveen A, et al. A gene-expression signature as a predictor of survival in breast cancer. N Engl J Med. 2002; 347:1999–2009. 10.1056/NEJMoa021967 12490681

[9] Sultan G, Zubair S, Tayubi IA, Dahms HU, Madar IH. Towards the early detection of ductal carcinoma (a common type of breast cancer) using biomarkers linked to the PPAR(γ) signaling pathway. Bioinformation. 2019; 15:799–805. 10.6026/97320630015799 31902979 PMC6936658

[10] Fahad Ullah M. Breast Cancer: Current Perspectives on the Disease Status. Adv Exp Med Biol. 2019; 1152:51–64. 10.1007/978-3-030-20301-6_4 31456179

[11] Sever R, Brugge JS. Signal transduction in cancer. Cold Spring Harb Perspect Med. 2015; 5:a006098. 10.1101/cshperspect.a006098 25833940 PMC4382731

[12] Chakravarthi BV, Nepal S, Varambally S. Genomic and Epigenomic Alterations in Cancer. Am J Pathol. 2016; 186:1724–35. 10.1016/j.ajpath.2016.02.023 27338107 PMC4929396

[13] Herceg Z, Hainaut P. Genetic and epigenetic alterations as biomarkers for cancer detection, diagnosis and prognosis. Mol Oncol. 2007; 1:26–41. 10.1016/j.molonc.2007.01.004 19383285 PMC5543860

[14] Kaszak I, Witkowska-Piłaszewicz O, Niewiadomska Z, Dworecka-Kaszak B, Ngosa Toka F, Jurka P. Role of Cadherins in Cancer-A Review. Int J Mol Sci. 2020; 21:7624. 10.3390/ijms21207624 33076339 PMC7589192

[15] de Agustín-Durán D, Mateos-White I, Fabra-Beser J, Gil-Sanz C. Stick around: Cell-Cell Adhesion Molecules during Neocortical Development. Cells. 2021; 10:118. 10.3390/cells10010118 33435191 PMC7826847

[16] Stelzer G, Rosen N, Plaschkes I, Zimmerman S, Twik M, Fishilevich S, Stein TI, Nudel R, Lieder I, Mazor Y, Kaplan S, Dahary D, Warshawsky D, et al. The GeneCards Suite: From Gene Data Mining to Disease Genome Sequence Analyses. Curr Protoc Bioinformatics. 2016; 54:1.30. 10.1002/cpbi.5 27322403

[17] Takeichi M. Cadherin cell adhesion receptors as a morphogenetic regulator. Science. 1991; 251:1451–5. 10.1126/science.2006419 2006419

[18] Yu W, Yang L, Li T, Zhang Y. Cadherin Signaling in Cancer: Its Functions and Role as a Therapeutic Target. Front Oncol. 2019; 9:989. 10.3389/fonc.2019.00989 31637214 PMC6788064

[19] Thiery JP. Epithelial-mesenchymal transitions in tumour progression. Nat Rev Cancer. 2002; 2:442–54. 10.1038/nrc822 12189386

[20] Kalluri R, Neilson EG. Epithelial-mesenchymal transition and its implications for fibrosis. J Clin Invest. 2003; 112:1776–84. 10.1172/JCI20530 14679171 PMC297008

[21] de Vasconcelos Azevedo FVP, Zóia MAP, Lopes DS, Gimenes SN, Vecchi L, Alves PT, Rodrigues RS, Silva ACA, Yoneyama KAG, Goulart LR, de Melo Rodrigues V. Antitumor and antimetastatic effects of PLA_2_-BthTX-II from Bothrops jararacussu venom on human breast cancer cells. Int J Biol Macromol. 2019; 135:261–73. 10.1016/j.ijbiomac.2019.05.164 31128190

[22] Fujii R, Imanishi Y, Shibata K, Sakai N, Sakamoto K, Shigetomi S, Habu N, Otsuka K, Sato Y, Watanabe Y, Ozawa H, Tomita T, Kameyama K, et al. Restoration of E-cadherin expression by selective Cox-2 inhibition and the clinical relevance of the epithelial-to-mesenchymal transition in head and neck squamous cell carcinoma. J Exp Clin Cancer Res. 2014; 33:40. 10.1186/1756-9966-33-40 24887090 PMC4030015

[23] Ribatti D, Tamma R, Annese T. Epithelial-Mesenchymal Transition in Cancer: A Historical Overview. Transl Oncol. 2020; 13:100773. 10.1016/j.tranon.2020.100773 32334405 PMC7182759

[24] Chu K, Boley KM, Moraes R, Barsky SH, Robertson FM. The paradox of E-cadherin: role in response to hypoxia in the tumor microenvironment and regulation of energy metabolism. Oncotarget. 2013; 4:446–62. 10.18632/oncotarget.872 23530113 PMC3717307

[25] Kemler R, Hierholzer A, Kanzler B, Kuppig S, Hansen K, Taketo MM, de Vries WN, Knowles BB, Solter D. Stabilization of beta-catenin in the mouse zygote leads to premature epithelial-mesenchymal transition in the epiblast. Development. 2004; 131:5817–24. 10.1242/dev.01458 15525667

[26] Klymkowsky MW. beta-catenin and its regulatory network. Hum Pathol. 2005; 36:225–7. 10.1016/j.humpath.2005.02.002 15791565

[27] Thorat MA, Balasubramanian R. Breast cancer prevention in high-risk women. Best Pract Res Clin Obstet Gynaecol. 2020; 65:18–31. 10.1016/j.bpobgyn.2019.11.006 31862315

[28] Lin CY, Lee CH, Chuang YH, Lee JY, Chiu YY, Wu Lee YH, Jong YJ, Hwang JK, Huang SH, Chen LC, Wu CH, Tu SH, Ho YS, Yang JM. Membrane protein-regulated networks across human cancers. Nat Commun. 2019; 10:3131. 10.1038/s41467-019-10920-8 31311925 PMC6635409

[29] Tsai HT, Huang CS, Tu CC, Liu CY, Huang CJ, Ho YS, Tu SH, Tseng LM, Huang CC. Multi-gene signature of microcalcification and risk prediction among Taiwanese breast cancer. Sci Rep. 2020; 10:18276. 10.1038/s41598-020-74982-1 33106505 PMC7588423

[30] Nguyen HD, Liao YC, Ho YS, Chen LC, Chang HW, Cheng TC, Liu D, Lee WR, Shen SC, Wu CH, Tu SH. The α9 Nicotinic Acetylcholine Receptor Mediates Nicotine-Induced PD-L1 Expression and Regulates Melanoma Cell Proliferation and Migration. Cancers (Basel). 2019; 11:1991. 10.3390/cancers11121991 31835799 PMC6966517

[31] Lee KL, Kuo YC, Ho YS, Huang YH. Triple-Negative Breast Cancer: Current Understanding and Future Therapeutic Breakthrough Targeting Cancer Stemness. Cancers (Basel). 2019; 11:1334. 10.3390/cancers11091334 31505803 PMC6769912

[32] Rhodes DR, Yu J, Shanker K, Deshpande N, Varambally R, Ghosh D, Barrette T, Pandey A, Chinnaiyan AM. ONCOMINE: a cancer microarray database and integrated data-mining platform. Neoplasia. 2004; 6:1–6. 10.1016/s1476-5586(04)80047-2 15068665 PMC1635162

[33] Kao TJ, Wu CC, Phan NN, Liu YH, Ta HD, Anuraga G, Wu YF, Lee KH, Chuang JY, Wang CY. Prognoses and genomic analyses of proteasome 26S subunit, ATPase (PSMC) family genes in clinical breast cancer. Aging (Albany NY). 2021; 13:17970. 10.18632/aging.203345 34329194 PMC8351721

[34] Wu PS, Yen JH, Wang CY, Chen PY, Hung JH, Wu MJ. 8-Hydroxydaidzein, an Isoflavone from Fermented Soybean, Induces Autophagy, Apoptosis, Differentiation, and Degradation of Oncoprotein BCR-ABL in K562 Cells. Biomedicines. 2020; 8:506. 10.3390/biomedicines8110506 33207739 PMC7696406

[35] Wu CC, Ekanem TI, Phan NN, Loan DT, Hou SY, Lee KH, Wang CY. Gene signatures and prognostic analyses of the Tob/BTG pituitary tumor-transforming gene (PTTG) family in clinical breast cancer patients. Int J Med Sci. 2020; 17:3112–24. 10.7150/ijms.49652 33173433 PMC7646110

[36] Lin YY, Wang CY, Phan NN, Chiao CC, Li CY, Sun Z, Hung JH, Chen YL, Yen MC, Weng TY, Hsu HP, Lai MD. PODXL2 maintains cellular stemness and promotes breast cancer development through the Rac1/Akt pathway. Int J Med Sci. 2020; 17:1639–51. 10.7150/ijms.46125 32669966 PMC7359396

[37] Hsu HP, Wang CY, Hsieh PY, Fang JH, Chen YL. Knockdown of serine/threonine-protein kinase 24 promotes tumorigenesis and myeloid-derived suppressor cell expansion in an orthotopic immunocompetent gastric cancer animal model. J Cancer. 2020; 11:213–28. 10.7150/jca.35821 31892988 PMC6930401

[38] Tang Z, Kang B, Li C, Chen T, Zhang Z. GEPIA2: an enhanced web server for large-scale expression profiling and interactive analysis. Nucleic Acids Res. 2019; 47:W556–60. 10.1093/nar/gkz430 31114875 PMC6602440

[39] Barrett T, Wilhite SE, Ledoux P, Evangelista C, Kim IF, Tomashevsky M, Marshall KA, Phillippy KH, Sherman PM, Holko M, Yefanov A, Lee H, Zhang N, et al. NCBI GEO: archive for functional genomics data sets--update. Nucleic Acids Res. 2013; 41:D991–5. 10.1093/nar/gks1193 23193258 PMC3531084

[40] Lin JC, Liu TP, Yang PM. CDKN2A-Inactivated Pancreatic Ductal Adenocarcinoma Exhibits Therapeutic Sensitivity to Paclitaxel: A Bioinformatics Study. J Clin Med. 2020; 9:4019. 10.3390/jcm9124019 33322698 PMC7763913

[41] Lin TY, Wang PW, Huang CH, Yang PM, Pan TL. Characterizing the Relapse Potential in Different Luminal Subtypes of Breast Cancers with Functional Proteomics. Int J Mol Sci. 2020; 21:6077. 10.3390/ijms21176077 32846884 PMC7504407

[42] Liu LW, Hsieh YY, Yang PM. Bioinformatics Data Mining Repurposes the JAK2 (Janus Kinase 2) Inhibitor Fedratinib for Treating Pancreatic Ductal Adenocarcinoma by Reversing the *KRAS* (Kirsten Rat Sarcoma 2 Viral Oncogene Homolog)-Driven Gene Signature. J Pers Med. 2020; 10:130. 10.3390/jpm10030130 32947833 PMC7563462

[43] Yang PM, Hsieh YY, Du JL, Yen SC, Hung CF. Sequential Interferon β-Cisplatin Treatment Enhances the Surface Exposure of Calreticulin in Cancer Cells via an Interferon Regulatory Factor 1-Dependent Manner. Biomolecules. 2020; 10:643. 10.3390/biom10040643 32326356 PMC7226424

[44] Yang PM, Lin LS, Liu TP. Sorafenib Inhibits Ribonucleotide Reductase Regulatory Subunit M2 (RRM2) in Hepatocellular Carcinoma Cells. Biomolecules. 2020; 10:117. 10.3390/biom10010117 31936661 PMC7022495

[45] Nagy Á, Munkácsy G, Győrffy B. Pancancer survival analysis of cancer hallmark genes. Sci Rep. 2021; 11:6047. 10.1038/s41598-021-84787-5 33723286 PMC7961001

[46] Lánczky A, Nagy Á, Bottai G, Munkácsy G, Szabó A, Santarpia L, Győrffy B. miRpower: a web-tool to validate survival-associated miRNAs utilizing expression data from 2178 breast cancer patients. Breast Cancer Res Treat. 2016; 160:439–46. 10.1007/s10549-016-4013-7 27744485

[47] Gao J, Aksoy BA, Dogrusoz U, Dresdner G, Gross B, Sumer SO, Sun Y, Jacobsen A, Sinha R, Larsson E, Cerami E, Sander C, Schultz N. Integrative analysis of complex cancer genomics and clinical profiles using the cBioPortal. Sci Signal. 2013; 6:pl1. 10.1126/scisignal.2004088 23550210 PMC4160307

[48] Sun CC, Li SJ, Hu W, Zhang J, Zhou Q, Liu C, Li LL, Songyang YY, Zhang F, Chen ZL, Li G, Bi ZY, Bi YY, et al. RETRACTED: Comprehensive Analysis of the Expression and Prognosis for E2Fs in Human Breast Cancer. Mol Ther. 2019; 27:1153–65. 10.1016/j.ymthe.2019.03.019 31010740 PMC6554685

[49] Hagerling C, Owyong M, Sitarama V, Wang CY, Lin C, van den Bijgaart RJ, Koopman CD, Brenot A, Nanjaraj A, Wärnberg F, Jirström K, Klein OD, Werb Z, Plaks V. LGR5 in breast cancer and ductal carcinoma *in situ*: a diagnostic and prognostic biomarker and a therapeutic target. BMC Cancer. 2020; 20:542. 10.1186/s12885-020-06986-z 32522170 PMC7285764

[50] Phan NN, Liu S, Wang CY, Hsu HP, Lai MD, Li CY, Chen CF, Chiao CC, Yen MC, Sun Z, Jiang JZ. Overexpressed gene signature of EPH receptor A/B family in cancer patients-comprehensive analyses from the public high-throughput database. Int J Clin Exp Pathol. 2020; 13:1220–42. 32509099 PMC7270671

[51] Uhlén M, Fagerberg L, Hallström BM, Lindskog C, Oksvold P, Mardinoglu A, Sivertsson Å, Kampf C, Sjöstedt E, Asplund A, Olsson I, Edlund K, Lundberg E, et al. Proteomics. Tissue-based map of the human proteome. Science. 2015; 347:1260419. 10.1126/science.1260419 25613900

[52] Jézéquel P, Gouraud W, Ben Azzouz F, Guérin-Charbonnel C, Juin PP, Lasla H, Campone M. bc-GenExMiner 4.5: new mining module computes breast cancer differential gene expression analyses. Database (Oxford). 2021; 2021:baab007. 10.1093/database/baab007 33599248 PMC7904047

[53] Barretina J, Caponigro G, Stransky N, Venkatesan K, Margolin AA, Kim S, Wilson CJ, Lehár J, Kryukov GV, Sonkin D, Reddy A, Liu M, Murray L, et al. The Cancer Cell Line Encyclopedia enables predictive modelling of anticancer drug sensitivity. Nature. 2012; 483:603–7. 10.1038/nature11003 22460905 PMC3320027

[54] Lawal B, Liu YL, Mokgautsi N, Khedkar H, Sumitra MR, Wu AT, Huang HS. Pharmacoinformatics and Preclinical Studies of NSC765690 and NSC765599, Potential STAT3/CDK2/4/6 Inhibitors with Antitumor Activities against NCI60 Human Tumor Cell Lines. Biomedicines. 2021; 9:92. 10.3390/biomedicines9010092 33477856 PMC7832910

[55] Lawal B, Lee CY, Mokgautsi N, Sumitra MR, Khedkar H, Wu AT, Huang HS. mTOR/EGFR/iNOS/MAP2K1/FGFR/TGFB1 Are Druggable Candidates for N-(2,4-Difluorophenyl)-2’,4’-Difluoro-4-Hydroxybiphenyl-3-Carboxamide (NSC765598), With Consequent Anticancer Implications. Front Oncol. 2021; 11:656738. 10.3389/fonc.2021.656738 33842373 PMC8034425

[56] Lawal B, Kuo YC, Sumitra MR, Wu AT, Huang HS. *In vivo* Pharmacokinetic and Anticancer Studies of HH-N25, a Selective Inhibitor of Topoisomerase I, and Hormonal Signaling for Treating Breast Cancer. J Inflamm Res. 2021; 14:4901–13. 10.2147/JIR.S329401 34588796 PMC8473721

[57] Lawal B, Kuo YC, Wu AT, Huang HS. BC-N102 suppress breast cancer tumorigenesis by interfering with cell cycle regulatory proteins and hormonal signaling, and induction of time-course arrest of cell cycle at G1/G0 phase. Int J Biol Sci. 2021; 17:3224–38. 10.7150/ijbs.62808 34421361 PMC8375223

[58] Modhukur V, Iljasenko T, Metsalu T, Lokk K, Laisk-Podar T, Vilo J. MethSurv: a web tool to perform multivariable survival analysis using DNA methylation data. Epigenomics. 2018; 10:277–88. 10.2217/epi-2017-0118 29264942

[59] Curtis C, Shah SP, Chin SF, Turashvili G, Rueda OM, Dunning MJ, Speed D, Lynch AG, Samarajiwa S, Yuan Y, Gräf S, Ha G, Haffari G, et al, and METABRIC Group. The genomic and transcriptomic architecture of 2,000 breast tumours reveals novel subgroups. Nature. 2012; 486:346–52. 10.1038/nature10983 22522925 PMC3440846

[60] Cancer Genome Atlas Network. Comprehensive molecular portraits of human breast tumours. Nature. 2012; 490:61–70. 10.1038/nature11412 23000897 PMC3465532

[61] Anuraga G, Wang WJ, Phan NN, An Ton NT, Ta HD, Berenice Prayugo F, Minh Xuan DT, Ku SC, Wu YF, Andriani V, Athoillah M, Lee KH, Wang CY. Potential Prognostic Biomarkers of NIMA (Never in Mitosis, Gene A)-Related Kinase (NEK) Family Members in Breast Cancer. J Pers Med. 2021; 11:1089. 10.3390/jpm11111089 34834441 PMC8625415

[62] Khoa Ta HD, Tang WC, Phan NN, Anuraga G, Hou SY, Chiao CC, Liu YH, Wu YF, Lee KH, Wang CY. Analysis of LAGEs Family Gene Signature and Prognostic Relevance in Breast Cancer. Diagnostics (Basel). 2021; 11:726. 10.3390/diagnostics11040726 33921749 PMC8074247

[63] Ta HD, Wang WJ, Phan NN, An Ton NT, Anuraga G, Ku SC, Wu YF, Wang CY, Lee KH. Potential Therapeutic and Prognostic Values of LSM Family Genes in Breast Cancer. Cancers (Basel). 2021; 13:4902. 10.3390/cancers13194902 34638387 PMC8508234

[64] Wu YH, Yeh IJ, Phan NN, Yen MC, Liu HL, Wang CY, Hsu HP. Severe acute respiratory syndrome coronavirus (SARS-CoV)-2 infection induces dysregulation of immunity: *in silico* gene expression analysis. Int J Med Sci. 2021; 18:1143–52. 10.7150/ijms.52256 33526974 PMC7847623

[65] Liu HL, Yeh IJ, Phan NN, Wu YH, Yen MC, Hung JH, Chiao CC, Chen CF, Sun Z, Jiang JZ, Hsu HP, Wang CY, Lai MD. Gene signatures of SARS-CoV/SARS-CoV-2-infected ferret lungs in short- and long-term models. Infect Genet Evol. 2020; 85:104438. 10.1016/j.meegid.2020.104438 32615317 PMC7832673

[66] Liu CH, Lu CH, Lin LT. Pandemic strategies with computational and structural biology against COVID-19: A retrospective. Comput Struct Biotechnol J. 2021; 20:187–92. 10.1016/j.csbj.2021.11.040 34900126 PMC8650801

[67] Lim HG, Hsiao SH, Fann YC, Lee YG. Robust Mutation Profiling of SARS-CoV-2 Variants from Multiple Raw Illumina Sequencing Data with Cloud Workflow. Genes (Basel). 2022; 13:686. 10.3390/genes13040686 35456492 PMC9028989

[68] Yu WL, Toh HS, Liao CT, Chang WT. A Double-Edged Sword-Cardiovascular Concerns of Potential Anti-COVID-19 Drugs. Cardiovasc Drugs Ther. 2021; 35:205–14. 10.1007/s10557-020-07024-7 32557011 PMC7297930

[69] Poly TN, Islam MM, Li YJ, Alsinglawi B, Hsu MH, Jian WS, Yang HC. Application of Artificial Intelligence for Screening COVID-19 Patients Using Digital Images: Meta-analysis. JMIR Med Inform. 2021; 9:e21394. 10.2196/21394 33764884 PMC8086786

[70] Yu CS, Chang SS, Chang TH, Wu JL, Lin YJ, Chien HF, Chen RJ. A COVID-19 Pandemic Artificial Intelligence-Based System With Deep Learning Forecasting and Automatic Statistical Data Acquisition: Development and Implementation Study. J Med Internet Res. 2021; 23:e27806. 10.2196/27806 33900932 PMC8139395

[71] Boyle EI, Weng S, Gollub J, Jin H, Botstein D, Cherry JM, Sherlock G. GO::TermFinder--open source software for accessing Gene Ontology information and finding significantly enriched Gene Ontology terms associated with a list of genes. Bioinformatics. 2004; 20:3710–5. 10.1093/bioinformatics/bth456 15297299 PMC3037731

[72] Wang CY, Chiao CC, Phan NN, Li CY, Sun ZD, Jiang JZ, Hung JH, Chen YL, Yen MC, Weng TY, Chen WC, Hsu HP, Lai MD. Gene signatures and potential therapeutic targets of amino acid metabolism in estrogen receptor-positive breast cancer. Am J Cancer Res. 2020; 10:95–113. 32064155 PMC7017735

[73] Xuan DT, Yeh IJ, Wu CC, Su CY, Liu HL, Chiao CC, Ku SC, Jiang JZ, Sun Z, Ta HD, Anuraga G, Wang CY, Yen MC. Comparison of Transcriptomic Signatures between Monkeypox-Infected Monkey and Human Cell Lines. J Immunol Res. 2022; 2022:3883822. 10.1155/2022/3883822 36093436 PMC9458371

[74] Sergushichev A. An algorithm for fast preranked gene set enrichment analysis using cumulative statistic calculation. BioRxiv. 2016. [Epub ahead of print].

[75] Korotkevich G, Sukhov V, Budin N, Shpak B, Artyomov MN, Sergushichev A. Fast gene set enrichment analysis. BioRxiv. 2021. [Epub ahead of print]. 10.1101/060012

[76] Chen PS, Hsu HP, Phan NN, Yen MC, Chen FW, Liu YW, Lin FP, Feng SY, Cheng TL, Yeh PH, Omar HA, Sun Z, Jiang JZ, et al. CCDC167 as a potential therapeutic target and regulator of cell cycle-related networks in breast cancer. Aging (Albany NY). 2021; 13:4157–81. 10.18632/aging.202382 33461170 PMC7906182

[77] Bakre A, Andersen LE, Meliopoulos V, Coleman K, Yan X, Brooks P, Crabtree J, Tompkins SM, Tripp RA. Identification of Host Kinase Genes Required for Influenza Virus Replication and the Regulatory Role of MicroRNAs. PLoS One. 2013; 8:e66796. 10.1371/journal.pone.0066796 23805279 PMC3689682

[78] Fujiwara Y, Saito M, Robles AI, Nishida M, Takeshita F, Watanabe M, Ochiya T, Yokota J, Kohno T, Harris CC, Tsuchiya N. A Nucleolar Stress-Specific p53-miR-101 Molecular Circuit Functions as an Intrinsic Tumor-Suppressor Network. EBioMedicine. 2018; 33:33–48. 10.1016/j.ebiom.2018.06.031 30049386 PMC6085539

[79] Wu YH, Yeh IJ, Phan NN, Yen MC, Hung JH, Chiao CC, Chen CF, Sun Z, Hsu HP, Wang CY, Lai MD. Gene signatures and potential therapeutic targets of Middle East respiratory syndrome coronavirus (MERS-CoV)-infected human lung adenocarcinoma epithelial cells. J Microbiol Immunol Infect. 2021; 54:845–57. 10.1016/j.jmii.2021.03.007 34176764 PMC7997684

[80] Li T, Fan J, Wang B, Traugh N, Chen Q, Liu JS, Li B, Liu XS. TIMER: A Web Server for Comprehensive Analysis of Tumor-Infiltrating Immune Cells. Cancer Res. 2017; 77:e108–10. 10.1158/0008-5472.CAN-17-0307 29092952 PMC6042652

[81] Li B, Severson E, Pignon JC, Zhao H, Li T, Novak J, Jiang P, Shen H, Aster JC, Rodig S, Signoretti S, Liu JS, Liu XS. Comprehensive analyses of tumor immunity: implications for cancer immunotherapy. Genome Biol. 2016; 17:174. 10.1186/s13059-016-1028-7 27549193 PMC4993001

[82] Amat S, Penault-Llorca F, Cure H, Le Bouedëc G, Achard JL, Van Praagh I, Feillel V, Mouret-Reynier MA, Dauplat J, Chollet P. Scarff-Bloom-Richardson (SBR) grading: a pleiotropic marker of chemosensitivity in invasive ductal breast carcinomas treated by neoadjuvant chemotherapy. Int J Oncol. 2002; 20:791–6. 11894126

[83] Galea MH, Blamey RW, Elston CE, Ellis IO. The Nottingham Prognostic Index in primary breast cancer. Breast Cancer Res Treat. 1992; 22:207–19. 10.1007/BF01840834 1391987

[84] Chava S, Gupta R. Identification of the Mutational Landscape of Gynecological Malignancies. J Cancer. 2020; 11:4870–83. 10.7150/jca.46174 32626534 PMC7330690

[85] Yang SY, Lheureux S, Karakasis K, Burnier JV, Bruce JP, Clouthier DL, Danesh A, Quevedo R, Dowar M, Hanna Y, Li T, Lu L, Xu W, et al. Landscape of genomic alterations in high-grade serous ovarian cancer from exceptional long- and short-term survivors. Genome Med. 2018; 10:81. 10.1186/s13073-018-0590-x 30382883 PMC6208125

[86] Ciriello G, Gatza ML, Beck AH, Wilkerson MD, Rhie SK, Pastore A, Zhang H, McLellan M, Yau C, Kandoth C, Bowlby R, Shen H, Hayat S, et al, and TCGA Research Network. Comprehensive Molecular Portraits of Invasive Lobular Breast Cancer. Cell. 2015; 163:506–19. 10.1016/j.cell.2015.09.033 26451490 PMC4603750

[87] Dabbs DJ, Schnitt SJ, Geyer FC, Weigelt B, Baehner FL, Decker T, Eusebi V, Fox SB, Ichihara S, Lakhani SR, Palacios J, Rakha E, Richardson AL, et al. Lobular neoplasia of the breast revisited with emphasis on the role of E-cadherin immunohistochemistry. Am J Surg Pathol. 2013; 37:e1–11. 10.1097/PAS.0b013e3182918a2b 23759937

[88] Gregory PA, Bert AG, Paterson EL, Barry SC, Tsykin A, Farshid G, Vadas MA, Khew-Goodall Y, Goodall GJ. The miR-200 family and miR-205 regulate epithelial to mesenchymal transition by targeting ZEB1 and SIP1. Nat Cell Biol. 2008; 10:593–601. 10.1038/ncb1722 18376396

[89] Park SM, Gaur AB, Lengyel E, Peter ME. The miR-200 family determines the epithelial phenotype of cancer cells by targeting the E-cadherin repressors ZEB1 and ZEB2. Genes Dev. 2008; 22:894–907. 10.1101/gad.1640608 18381893 PMC2279201

[90] Korpal M, Lee ES, Hu G, Kang Y. The miR-200 family inhibits epithelial-mesenchymal transition and cancer cell migration by direct targeting of E-cadherin transcriptional repressors ZEB1 and ZEB2. J Biol Chem. 2008; 283:14910–4. 10.1074/jbc.C800074200 18411277 PMC3258899

[91] Fontana A, Barbano R, Dama E, Pasculli B, Rendina M, Morritti MG, Melocchi V, Castelvetere M, Valori VM, Ravaioli S, Bravaccini S, Ciuffreda L, Graziano P, et al. Combined analysis of miR-200 family and its significance for breast cancer. Sci Rep. 2021; 11:2980. 10.1038/s41598-021-82286-1 33536459 PMC7859396

[92] Thiery JP. Epithelial-mesenchymal transitions in development and pathologies. Curr Opin Cell Biol. 2003; 15:740–6. 10.1016/j.ceb.2003.10.006 14644200

[93] Nakajima S, Doi R, Toyoda E, Tsuji S, Wada M, Koizumi M, Tulachan SS, Ito D, Kami K, Mori T, Kawaguchi Y, Fujimoto K, Hosotani R, Imamura M. N-cadherin expression and epithelial-mesenchymal transition in pancreatic carcinoma. Clin Cancer Res. 2004; 10:4125–33. 10.1158/1078-0432.CCR-0578-03 15217949

[94] Hazan RB, Qiao R, Keren R, Badano I, Suyama K. Cadherin switch in tumor progression. Ann N Y Acad Sci. 2004; 1014:155–63. 10.1196/annals.1294.016 15153430

[95] Bartolomé RA, Torres S, Isern de Val S, Escudero-Paniagua B, Calviño E, Teixidó J, Casal JI. VE-cadherin RGD motifs promote metastasis and constitute a potential therapeutic target in melanoma and breast cancers. Oncotarget. 2017; 8:215–27. 10.18632/oncotarget.13832 27966446 PMC5352113

[96] Huang TH, Mokgautsi N, Huang YJ, Wu AT, Huang HS. Comprehensive Omics Analysis of a Novel Small-Molecule Inhibitor of Chemoresistant Oncogenic Signatures in Colorectal Cancer Cell with Antitumor Effects. Cells. 2021; 10:1970. 10.3390/cells10081970 34440739 PMC8392328

[97] Yadav VK, Huang YJ, George TA, Wei PL, Sumitra MR, Ho CL, Chang TH, Wu AT, Huang HS. Preclinical Evaluation of the Novel Small-Molecule MSI-N1014 for Treating Drug-Resistant Colon Cancer via the LGR5/β-catenin/miR-142-3p Network and Reducing Cancer-Associated Fibroblast Transformation. Cancers (Basel). 2020; 12:1590. 10.3390/cancers12061590 32560222 PMC7352915

[98] Lawal B, Wang YC, Wu AT, Huang HS. Pro-Oncogenic c-Met/EGFR, Biomarker Signatures of the Tumor Microenvironment are Clinical and Therapy Response Prognosticators in Colorectal Cancer, and Therapeutic Targets of 3-Phenyl-2H-benzo[e][1,3]-Oxazine-2,4(3H)-Dione Derivatives. Front Pharmacol. 2021; 12:691234. 10.3389/fphar.2021.691234 34512327 PMC8429938

[99] Chung CC, Huang TY, Chu HR, De Luca R, Candelotti E, Huang CH, Yang YS, Incerpi S, Pedersen JZ, Lin CY, Huang HM, Lee SY, Li ZL, et al. Heteronemin and tetrac derivatives suppress non-small cell lung cancer growth via ERK1/2 inhibition. Food Chem Toxicol. 2022; 161:112850. 10.1016/j.fct.2022.112850 35151786

[100] Hsiao SH, Chen WT, Chung CL, Chou YT, Lin SE, Hong SY, Chang JH, Chang TH, Chien LN. Comparative survival analysis of platinum-based adjuvant chemotherapy for early-stage squamous cell carcinoma and adenocarcinoma of the lung. Cancer Med. 2022; 11:2067–78. 10.1002/cam4.4570 35274494 PMC9119352

[101] Kuo KT, Lin CH, Wang CH, Pikatan NW, Yadav VK, Fong IH, Yeh CT, Lee WH, Huang WC. HNMT Upregulation Induces Cancer Stem Cell Formation and Confers Protection against Oxidative Stress through Interaction with HER2 in Non-Small-Cell Lung Cancer. Int J Mol Sci. 2022; 23:1663. 10.3390/ijms23031663 35163585 PMC8835856

[102] Tseng PC, Chen CL, Lee KY, Feng PH, Wang YC, Satria RD, Lin CF. Epithelial-to-mesenchymal transition hinders interferon-γ-dependent immunosurveillance in lung cancer cells. Cancer Lett. 2022; 539:215712. 10.1016/j.canlet.2022.215712 35490920

[103] Lee HC, Lu YH, Huang YL, Huang SL, Chuang HC. Air Pollution Effects to the Subtype and Severity of Lung Cancers. Front Med (Lausanne). 2022; 9:835026. 10.3389/fmed.2022.835026 35433740 PMC9008538

[104] Chen YL, Lee KT, Wang CY, Shen CH, Chen SC, Chung WP, Hsu YT, Kuo YL, Chen PS, Cheung CH, Chang CP, Shen MR, Hsu HP. Low expression of cytosolic NOTCH1 predicts poor prognosis of breast cancer patients. Am J Cancer Res. 2022; 12:2084–101. 35693094 PMC9185622

[105] Hagerling C, Gonzalez H, Salari K, Wang CY, Lin C, Robles I, van Gogh M, Dejmek A, Jirström K, Werb Z. Immune effector monocyte-neutrophil cooperation induced by the primary tumor prevents metastatic progression of breast cancer. Proc Natl Acad Sci USA. 2019; 116:21704–14. 10.1073/pnas.1907660116 31591235 PMC6815161

[106] Wang WJ, Lai HY, Zhang F, Shen WJ, Chu PY, Liang HY, Liu YB, Wang JM. MCL1 participates in leptin-promoted mitochondrial fusion and contributes to drug resistance in gallbladder cancer. JCI Insight. 2021; 6:e135438. 10.1172/jci.insight.135438 34156978 PMC8410075

[107] Chou CW, Hsieh YH, Ku SC, Shen WJ, Anuraga G, Khoa Ta HD, Lee KH, Lee YC, Lin CH, Wang CY, Wang WJ. Potential Prognostic Biomarkers of OSBPL Family Genes in Patients with Pancreatic Ductal Adenocarcinoma. Biomedicines. 2021; 9:1601. 10.3390/biomedicines9111601 34829830 PMC8615799

[108] Ramezani M, Baharzadeh F, Almasi A, Sadeghi M. A Systematic Review and Meta-Analysis: Evaluation of the β-Human Papillomavirus in Immunosuppressed Individuals with Cutaneous Squamous Cell Carcinoma. Biomedicine (Taipei). 2020; 10:1–10. 10.37796/2211-8039.1110 33854928 PMC7735980

[109] Lindsey S, Langhans SA. Crosstalk of Oncogenic Signaling Pathways during Epithelial-Mesenchymal Transition. Front Oncol. 2014; 4:358. 10.3389/fonc.2014.00358 25566498 PMC4263086

[110] Falzone L, Candido S, Salemi R, Basile MS, Scalisi A, McCubrey JA, Torino F, Signorelli SS, Montella M, Libra M. Computational identification of microRNAs associated to both epithelial to mesenchymal transition and NGAL/MMP-9 pathways in bladder cancer. Oncotarget. 2016; 7:72758–66. 10.18632/oncotarget.11805 27602581 PMC5341942

[111] McCubrey JA, Fitzgerald TL, Yang LV, Lertpiriyapong K, Steelman LS, Abrams SL, Montalto G, Cervello M, Neri LM, Cocco L, Martelli AM, Laidler P, Dulińska-Litewka J, et al. Roles of GSK-3 and microRNAs on epithelial mesenchymal transition and cancer stem cells. Oncotarget. 2017; 8:14221–50. 10.18632/oncotarget.13991 27999207 PMC5355173

[112] Lade-Keller J, Riber-Hansen R, Guldberg P, Schmidt H, Hamilton-Dutoit SJ, Steiniche T. E- to N-cadherin switch in melanoma is associated with decreased expression of phosphatase and tensin homolog and cancer progression. Br J Dermatol. 2013; 169:618–28. 10.1111/bjd.12426 23662813

[113] Araki K, Shimura T, Suzuki H, Tsutsumi S, Wada W, Yajima T, Kobayahi T, Kubo N, Kuwano H. E/N-cadherin switch mediates cancer progression via TGF-β-induced epithelial-to-mesenchymal transition in extrahepatic cholangiocarcinoma. Br J Cancer. 2011; 105:1885–93. 10.1038/bjc.2011.452 22068819 PMC3251878

[114] Aleskandarany MA, Negm OH, Green AR, Ahmed MA, Nolan CC, Tighe PJ, Ellis IO, Rakha EA. Epithelial mesenchymal transition in early invasive breast cancer: an immunohistochemical and reverse phase protein array study. Breast Cancer Res Treat. 2014; 145:339–48. 10.1007/s10549-014-2927-5 24771047

[115] Gravdal K, Halvorsen OJ, Haukaas SA, Akslen LA. A switch from E-cadherin to N-cadherin expression indicates epithelial to mesenchymal transition and is of strong and independent importance for the progress of prostate cancer. Clin Cancer Res. 2007; 13:7003–11. 10.1158/1078-0432.CCR-07-1263 18056176

[116] Gröger CJ, Grubinger M, Waldhör T, Vierlinger K, Mikulits W. Meta-analysis of gene expression signatures defining the epithelial to mesenchymal transition during cancer progression. PLoS One. 2012; 7:e51136. 10.1371/journal.pone.0051136 23251436 PMC3519484

[117] Thiery JP, Acloque H, Huang RY, Nieto MA. Epithelial-mesenchymal transitions in development and disease. Cell. 2009; 139:871–90. 10.1016/j.cell.2009.11.007 19945376

[118] Peinado H, Olmeda D, Cano A. Snail, Zeb and bHLH factors in tumour progression: an alliance against the epithelial phenotype? Nat Rev Cancer. 2007; 7:415–28. 10.1038/nrc2131 17508028

[119] Chen JH, Huang WC, Bamodu OA, Chang PM, Chao TY, Huang TH. Monospecific antibody targeting of CDH11 inhibits epithelial-to-mesenchymal transition and represses cancer stem cell-like phenotype by up-regulating miR-335 in metastatic breast cancer, *in vitro* and *in vivo*. BMC Cancer. 2019; 19:634. 10.1186/s12885-019-5811-1 31248373 PMC6598338

[120] Kim NH, Choi SH, Lee TR, Lee CH, Lee AY. Cadherin 11, a miR-675 target, induces N-cadherin expression and epithelial-mesenchymal transition in melasma. J Invest Dermatol. 2014; 134:2967–76. 10.1038/jid.2014.257 24940649

[121] Schneider DJ, Wu M, Le TT, Cho SH, Brenner MB, Blackburn MR, Agarwal SK. Cadherin-11 contributes to pulmonary fibrosis: potential role in TGF-β production and epithelial to mesenchymal transition. FASEB J. 2012; 26:503–12. 10.1096/fj.11-186098 21990376 PMC3290437

[122] Jakubzig B, Baltes F, Henze S, Schlesinger M, Bendas G. Mechanisms of Matrix-Induced Chemoresistance of Breast Cancer Cells-Deciphering Novel Potential Targets for a Cell Sensitization. Cancers (Basel). 2018; 10:495. 10.3390/cancers10120495 30563275 PMC6315379

[123] McCormack VA, dos Santos Silva I. Breast density and parenchymal patterns as markers of breast cancer risk: a meta-analysis. Cancer Epidemiol Biomarkers Prev. 2006; 15:1159–69. 10.1158/1055-9965.EPI-06-0034 16775176

[124] Boyd NF, Martin LJ, Yaffe MJ, Minkin S. Mammographic density and breast cancer risk: current understanding and future prospects. Breast Cancer Res. 2011; 13:223. 10.1186/bcr2942 22114898 PMC3326547

[125] Nazari SS, Mukherjee P. An overview of mammographic density and its association with breast cancer. Breast Cancer. 2018; 25:259–67. 10.1007/s12282-018-0857-5 29651637 PMC5906528

[126] Liu J, Shen JX, Wu HT, Li XL, Wen XF, Du CW, Zhang GJ. Collagen 1A1 (COL1A1) promotes metastasis of breast cancer and is a potential therapeutic target. Discov Med. 2018; 25:211–23. 29906404

[127] Shi RZ, He YF, Wen J, Niu YN, Gao Y, Liu LH, Zhang XP, Wang Y, Zhang XL, Zhang HF, Chen M, Hu XL. Epithelial cell adhesion molecule promotes breast cancer resistance protein-mediated multidrug resistance in breast cancer by inducing partial epithelial-mesenchymal transition. Cell Biol Int. 2021; 45:1644–53. 10.1002/cbin.11598 33760350

[128] Tryndyak VP, Beland FA, Pogribny IP. E-cadherin transcriptional down-regulation by epigenetic and microRNA-200 family alterations is related to mesenchymal and drug-resistant phenotypes in human breast cancer cells. Int J Cancer. 2010; 126:2575–83. 10.1002/ijc.24972 19839049

[129] Burk U, Schubert J, Wellner U, Schmalhofer O, Vincan E, Spaderna S, Brabletz T. A reciprocal repression between ZEB1 and members of the miR-200 family promotes EMT and invasion in cancer cells. EMBO Rep. 2008; 9:582–9. 10.1038/embor.2008.74 18483486 PMC2396950

[130] Miotto E, Sabbioni S, Veronese A, Calin GA, Gullini S, Liboni A, Gramantieri L, Bolondi L, Ferrazzi E, Gafà R, Lanza G, Negrini M. Frequent aberrant methylation of the CDH4 gene promoter in human colorectal and gastric cancer. Cancer Res. 2004; 64:8156–9. 10.1158/0008-5472.CAN-04-3000 15548679

[131] Li L, Ying J, Li H, Zhang Y, Shu X, Fan Y, Tan J, Cao Y, Tsao SW, Srivastava G, Chan AT, Tao Q. The human cadherin 11 is a pro-apoptotic tumor suppressor modulating cell stemness through Wnt/β-catenin signaling and silenced in common carcinomas. Oncogene. 2012; 31:3901–12. 10.1038/onc.2011.541 22139084 PMC3426851

[132] Sarrió D, Rodriguez-Pinilla SM, Hardisson D, Cano A, Moreno-Bueno G, Palacios J. Epithelial-mesenchymal transition in breast cancer relates to the basal-like phenotype. Cancer Res. 2008; 68:989–97. 10.1158/0008-5472.CAN-07-2017 18281472

[133] Tomita K, van Bokhoven A, van Leenders GJ, Ruijter ET, Jansen CF, Bussemakers MJ, Schalken JA. Cadherin switching in human prostate cancer progression. Cancer Res. 2000; 60:3650–4. 10910081

[134] Li Y, Guo Z, Chen H, Dong Z, Pan ZK, Ding H, Su SB, Huang S. HOXC8-Dependent Cadherin 11 Expression Facilitates Breast Cancer Cell Migration through Trio and Rac. Genes Cancer. 2011; 2:880–8. 10.1177/1947601911433129 22593800 PMC3352157

[135] Zhao J, Li P, Feng H, Wang P, Zong Y, Ma J, Zhang Z, Chen X, Zheng M, Zhu Z, Lu A. Cadherin-12 contributes to tumorigenicity in colorectal cancer by promoting migration, invasion, adhersion and angiogenesis. J Transl Med. 2013; 11:288. 10.1186/1479-5876-11-288 24237488 PMC3879717

[136] Wang M, Long K, Li E, Li L, Li B, Ci S, He L, Pan F, Hu Z, Guo Z. DNA polymerase beta modulates cancer progression via enhancing CDH13 expression by promoter demethylation. Oncogene. 2020; 39:5507–19. 10.1038/s41388-020-1386-1 32641859

[137] Krishnamachary B, Zagzag D, Nagasawa H, Rainey K, Okuyama H, Baek JH, Semenza GL. Hypoxia-inducible factor-1-dependent repression of E-cadherin in von Hippel-Lindau tumor suppressor-null renal cell carcinoma mediated by TCF3, ZFHX1A, and ZFHX1B. Cancer Res. 2006; 66:2725–31. 10.1158/0008-5472.CAN-05-3719 16510593

[138] Toyooka S, Toyooka KO, Harada K, Miyajima K, Makarla P, Sathyanarayana UG, Yin J, Sato F, Shivapurkar N, Meltzer SJ, Gazdar AF. Aberrant methylation of the CDH13 (H-cadherin) promoter region in colorectal cancers and adenomas. Cancer Res. 2002; 62:3382–6. 12067979

[139] Takeuchi T, Misaki A, Sonobe H, Liang SB, Ohtsuki Y. Is T-cadherin (CDH13, H-cadherin) expression related to lung metastasis of osteosarcoma? Histopathology. 2000; 37:193–4. 10.1046/j.1365-2559.2000.00985-5.x 10931247

[140] Calì B, Molon B, Viola A. Tuning cancer fate: the unremitting role of host immunity. Open Biol. 2017; 7:170006. 10.1098/rsob.170006 28404796 PMC5413907

[141] Nawijn MC, Hackett TL, Postma DS, van Oosterhout AJ, Heijink IH. E-cadherin: gatekeeper of airway mucosa and allergic sensitization. Trends Immunol. 2011; 32:248–55. 10.1016/j.it.2011.03.004 21493142

[142] Jiang Y, Wan T, Chen G, Xiu F, Xia D, Zhang W, Zhou X, Cao X. DC-CLM, a cadherin-like molecule cloned from human dendritic cells, inhibits growth of breast cancer cells. J Cancer Res Clin Oncol. 2003; 129:57–64. 10.1007/s00432-002-0404-8 12618902 PMC12161908

[143] Pai SG, Carneiro BA, Mota JM, Costa R, Leite CA, Barroso-Sousa R, Kaplan JB, Chae YK, Giles FJ. Wnt/beta-catenin pathway: modulating anticancer immune response. J Hematol Oncol. 2017; 10:101. 10.1186/s13045-017-0471-6 28476164 PMC5420131

[144] Jang GB, Kim JY, Cho SD, Park KS, Jung JY, Lee HY, Hong IS, Nam JS. Blockade of Wnt/β-catenin signaling suppresses breast cancer metastasis by inhibiting CSC-like phenotype. Sci Rep. 2015; 5:12465. 10.1038/srep12465 26202299 PMC5378883

[145] Gattinoni L, Zhong XS, Palmer DC, Ji Y, Hinrichs CS, Yu Z, Wrzesinski C, Boni A, Cassard L, Garvin LM, Paulos CM, Muranski P, Restifo NP. Wnt signaling arrests effector T cell differentiation and generates CD8+ memory stem cells. Nat Med. 2009; 15:808–13. 10.1038/nm.1982 19525962 PMC2707501

[146] Iglesia MD, Parker JS, Hoadley KA, Serody JS, Perou CM, Vincent BG. Genomic Analysis of Immune Cell Infiltrates Across 11 Tumor Types. J Natl Cancer Inst. 2016; 108:djw144. 10.1093/jnci/djw144 27335052 PMC5241901

[147] Meng X, Huang Z, Teng F, Xing L, Yu J. Predictive biomarkers in PD-1/PD-L1 checkpoint blockade immunotherapy. Cancer Treat Rev. 2015; 41:868–76. 10.1016/j.ctrv.2015.11.001 26589760

[148] French JJ, Cresswell J, Wong WK, Seymour K, Charnley RM, Kirby JA. T cell adhesion and cytolysis of pancreatic cancer cells: a role for E-cadherin in immunotherapy? Br J Cancer. 2002; 87:1034–41. 10.1038/sj.bjc.6600597 12434297 PMC2364324

