# Supplementary material for: Comprehensive analysis of prognostic significance of cadherin (CDH) gene family in breast cancer
Source: Aging (Albany NY). 2022 Oct 25;14(20):8498–567. doi: 10.18632/aging.204357 (PMC9648792; doi:10.18632/aging.204357)
Supplement: Supplementary Figures [file aging-14-204357-s001.pdf]

SUPPLEMENTARY FIGURES

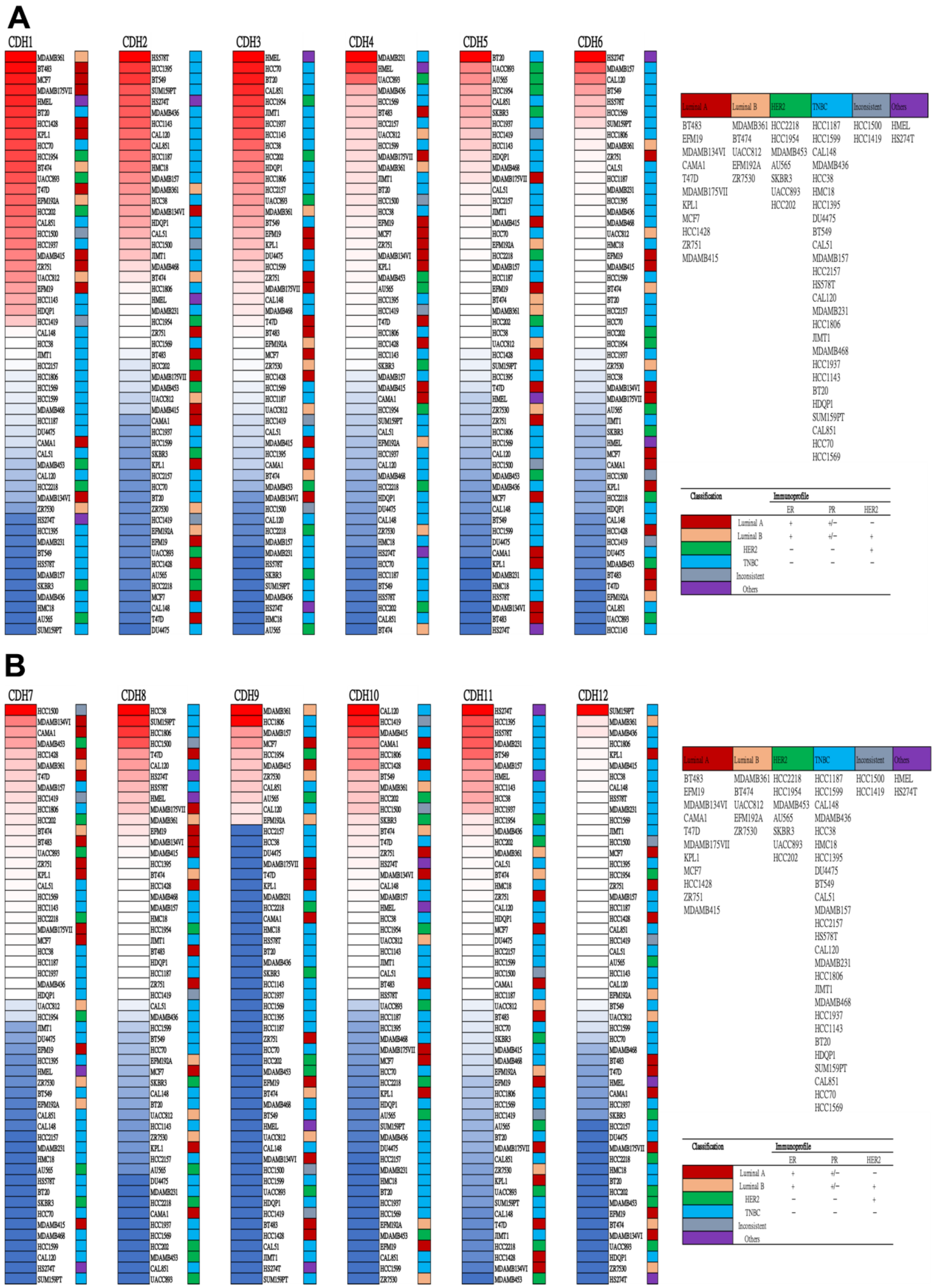

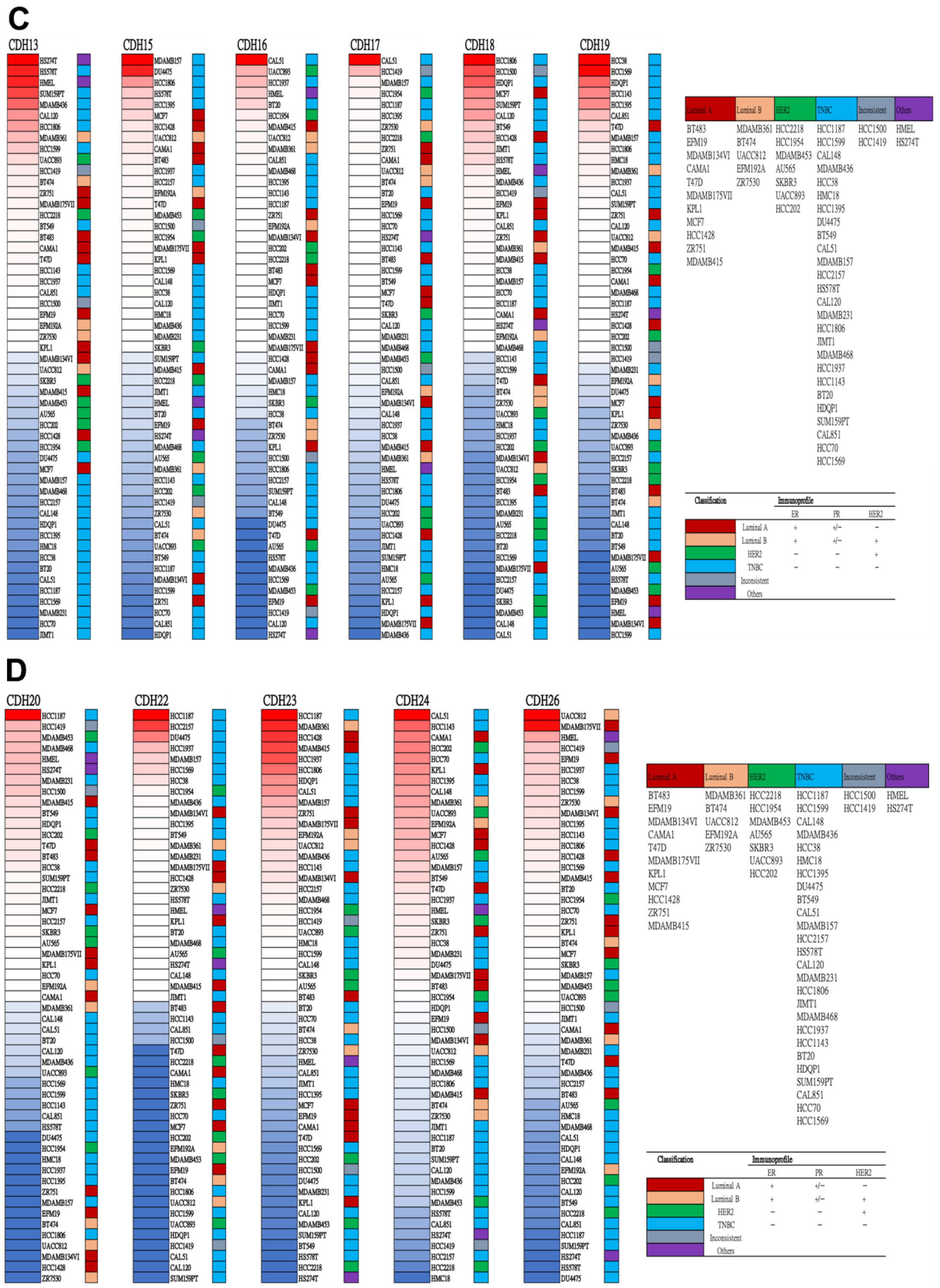

Supplementary Figure 1. (**A**–**D**) Molecular subtypes of breast cancer cell lines with expressions of CDH family. Colored columns on the right side displayed the molecular subtypes of each cell line. “Inconsistent” denoted cell lines that are inconsistently annotated regarding the status of markers. “Others” included two cell lines that were not breast cancers (HMEL, engineered breast; HS274T, breast fibroblast). TNBC, triple negative breast cancer.

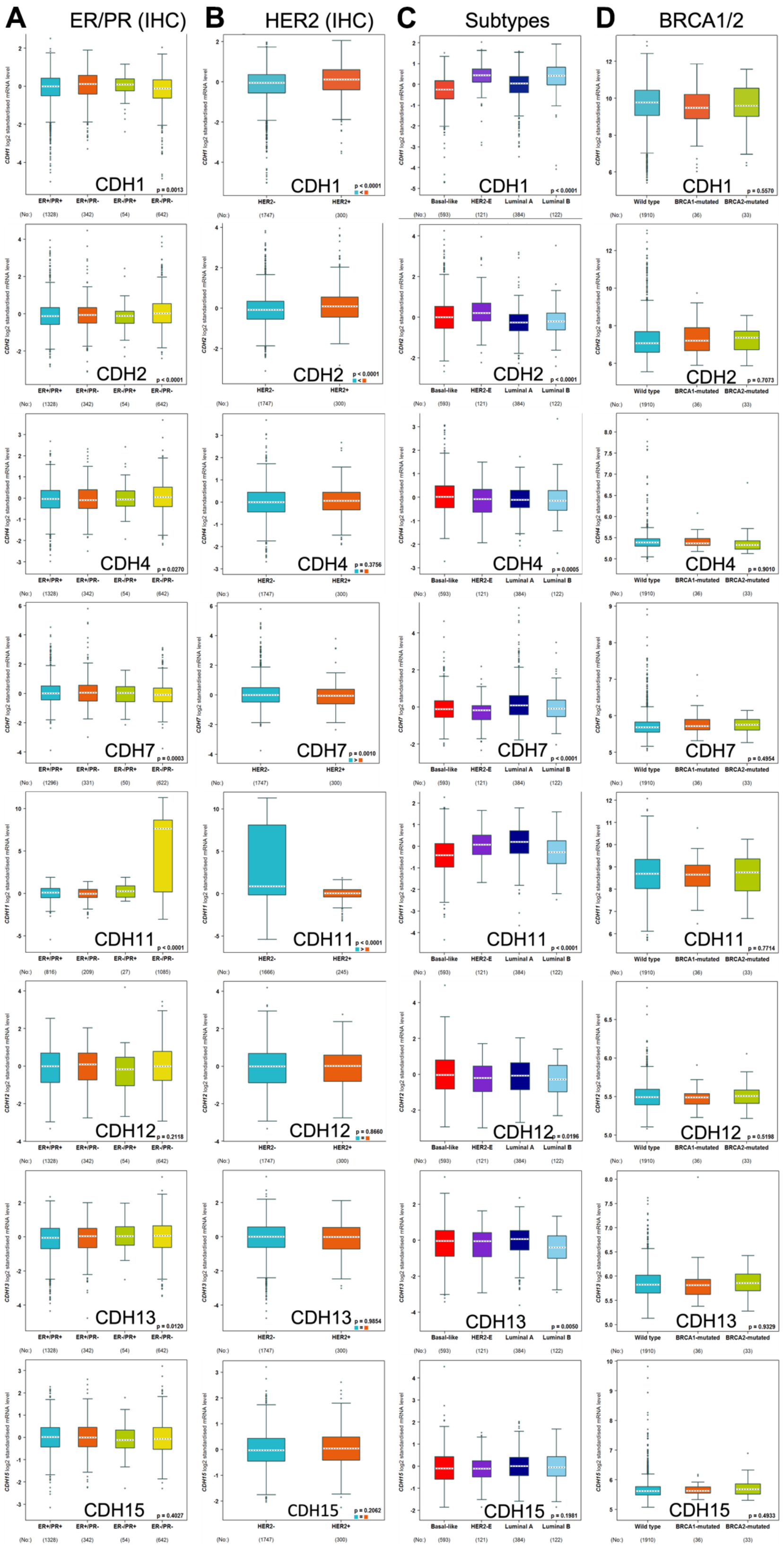

Supplementary Figure 2. Clinical indicators for treatment and subtypes of breast invasive carcinoma patients with cadherin 1 *(CDH1)/2/4/7/11/12/13/15* expressions. (**A**) Estrogen receptor (ER)/progesterone (PR) expression with *CDH* family genes co-expression. (**B**) Human epidermal growth factor receptor-2 (HER2) expression with *CDH* family genes co-expression. (**C**) Subtypes of breast invasive carcinoma with *CDH* family genes co-expression. (**D**) Breast cancer gene-1 *(BRCA1)/ BRCA2* mutations with *CDH* family genes co-expression.

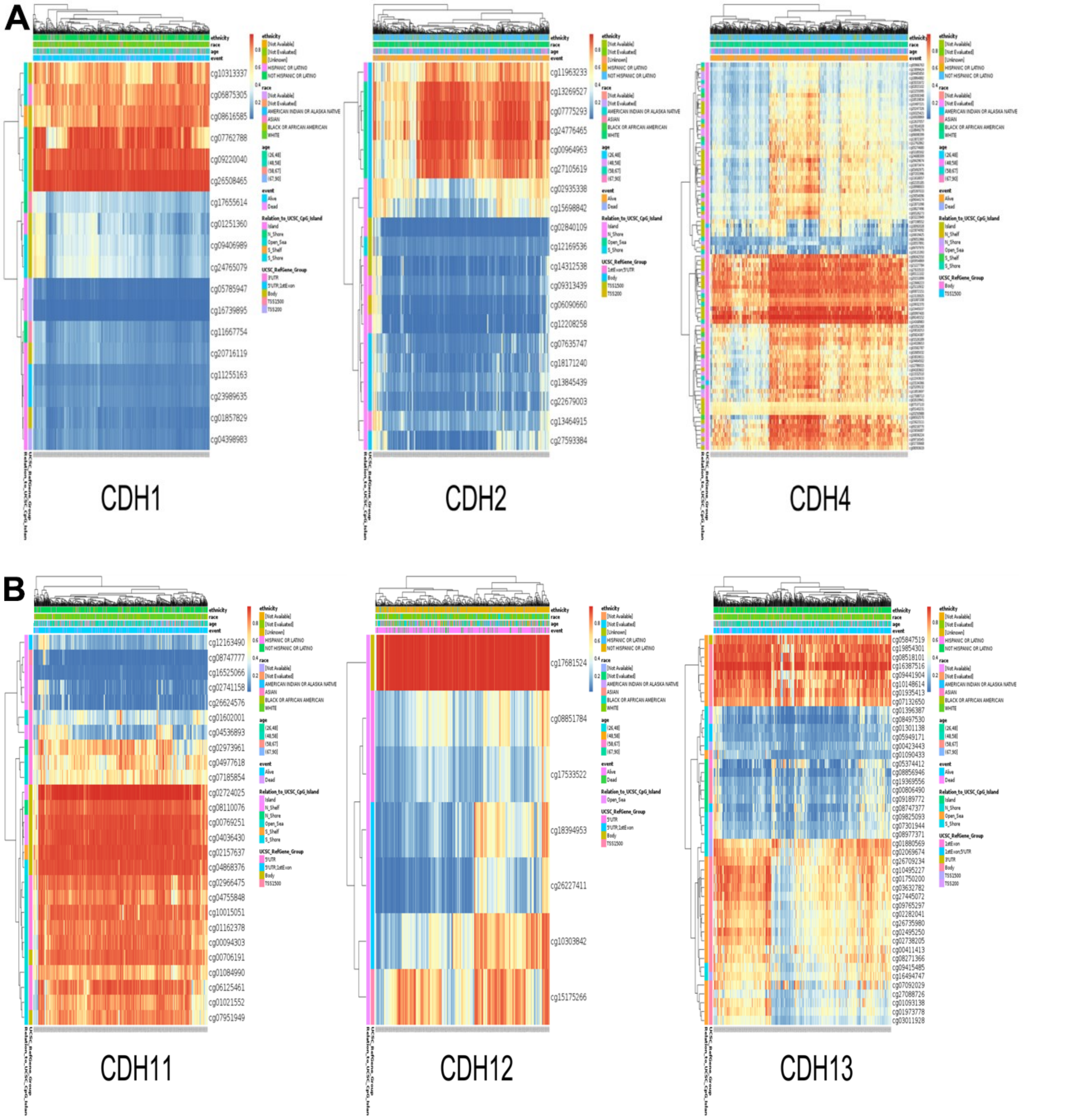

Supplementary Figure 3. Heatmap of DNA methylation expression levels of *CDH1/2/4/11/12/13* in breast cancer by the MethSurv platform. Blue bars indicated low expressions, while red bars indicated high expressions. Different colored boxes indicated different ethnicities, races, ages, events, relation to UCSC CpG island and UCSC RefGene Groups. DNA methylation status was represented as β-values (ranging from 0 to 1). (**A**) Among *CDH1*, cg26508465 and cg09220040 showed the highest levels of DNA methylation; cg24776465 showed the highest level among *CDH2*; cg6140152 showed the highest level among *CDH4*; (**B**) cg02724025 showed the highest level among *CDH11*; cg17681524 showed the highest level among *CDH12*; cg16387516 showed the highest level among *CDH13*.

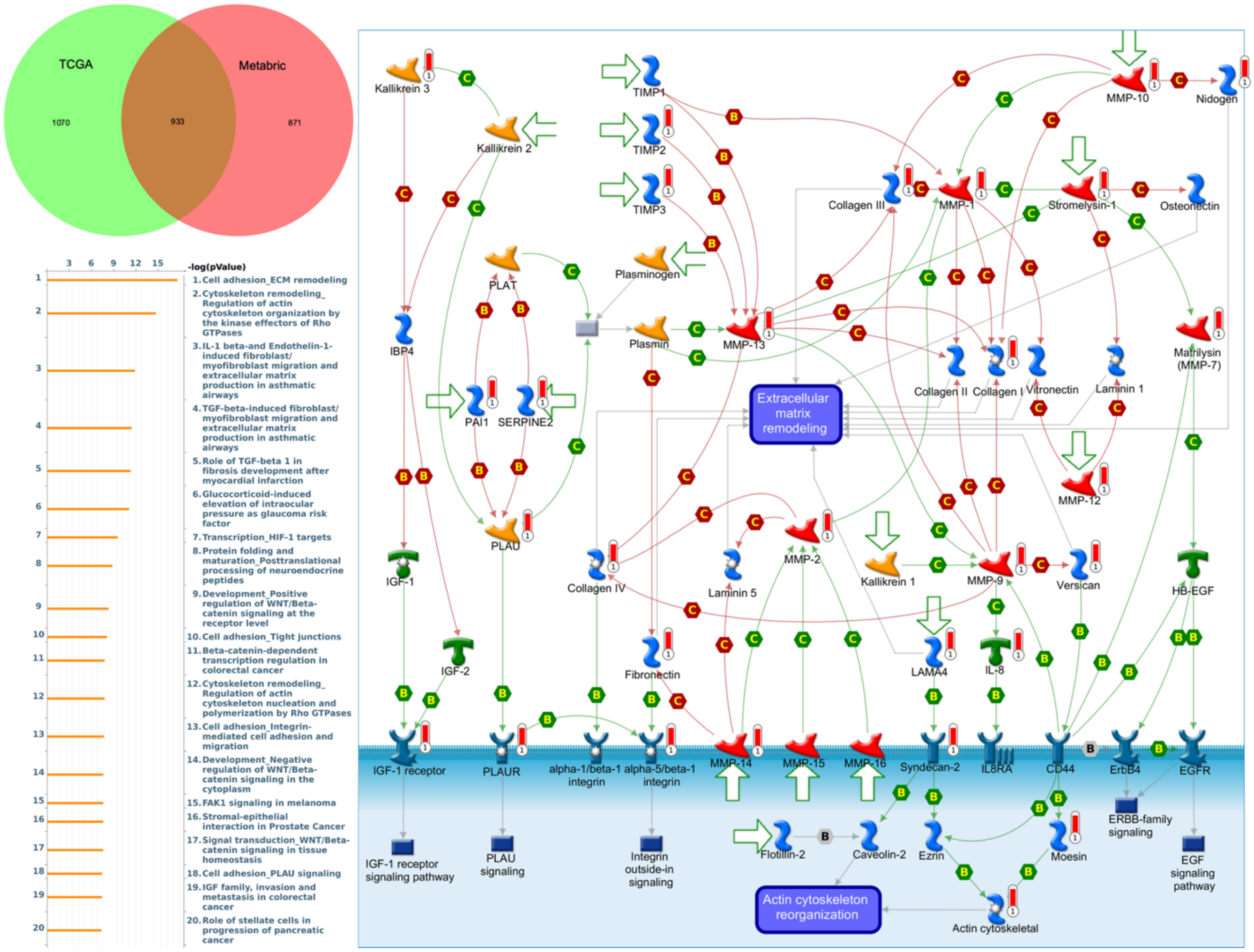

Supplementary Figure 4. MetaCore enrichment pathway analysis of genes co-expressed with cadherin 2 (*CDH2*). Top 10% of expression gene lists from both Metabric (1804 genes) and TCGA (2003 genes) were extracted. Overlapped (933) genes were integrated to implement pathway analysis, which formed a pathway list ordered by the -log *p*-value. “Cell adhesion_ECM remodeling” was at the top of the pathway list when performing the “biological process” analysis. The figure demonstrates the interactions between genes and proteins. Symbols represent proteins. Arrows depict protein interactions (green, activation; red, inhibition). Thermometer-like histograms indicate microarray gene expression (blue, down-regulation; red, up-regulation).

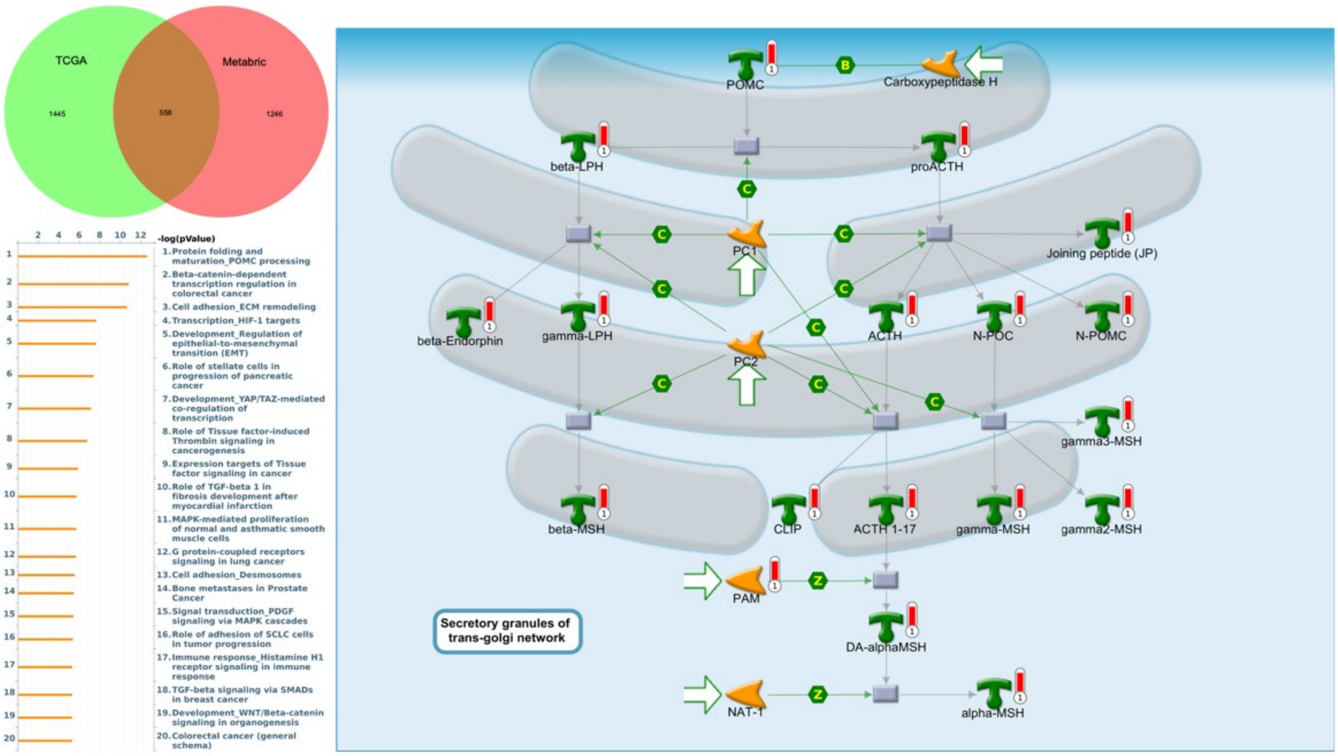

Supplementary Figure 5. MetaCore enrichment pathway analysis of genes co-expressed with cadherin 4 (*CDH4*). Top 10% of expression gene lists from both Metabric (1804 genes) and TCGA (2003 genes) were extracted. Overlapped (558) genes were integrated to implement pathway analysis, which formed a pathway list ordered by the -log *p*-value. “Protein folding and maturation_POMC processing” was at the top of the pathway list when performing the “biological process” analysis. The figure demonstrates the interactions between genes and proteins. Symbols represent proteins. Arrows depict protein interactions (green, activation; red, inhibition). Thermometer-like histograms indicate microarray gene expression (blue, down-regulation; red, up-regulation).

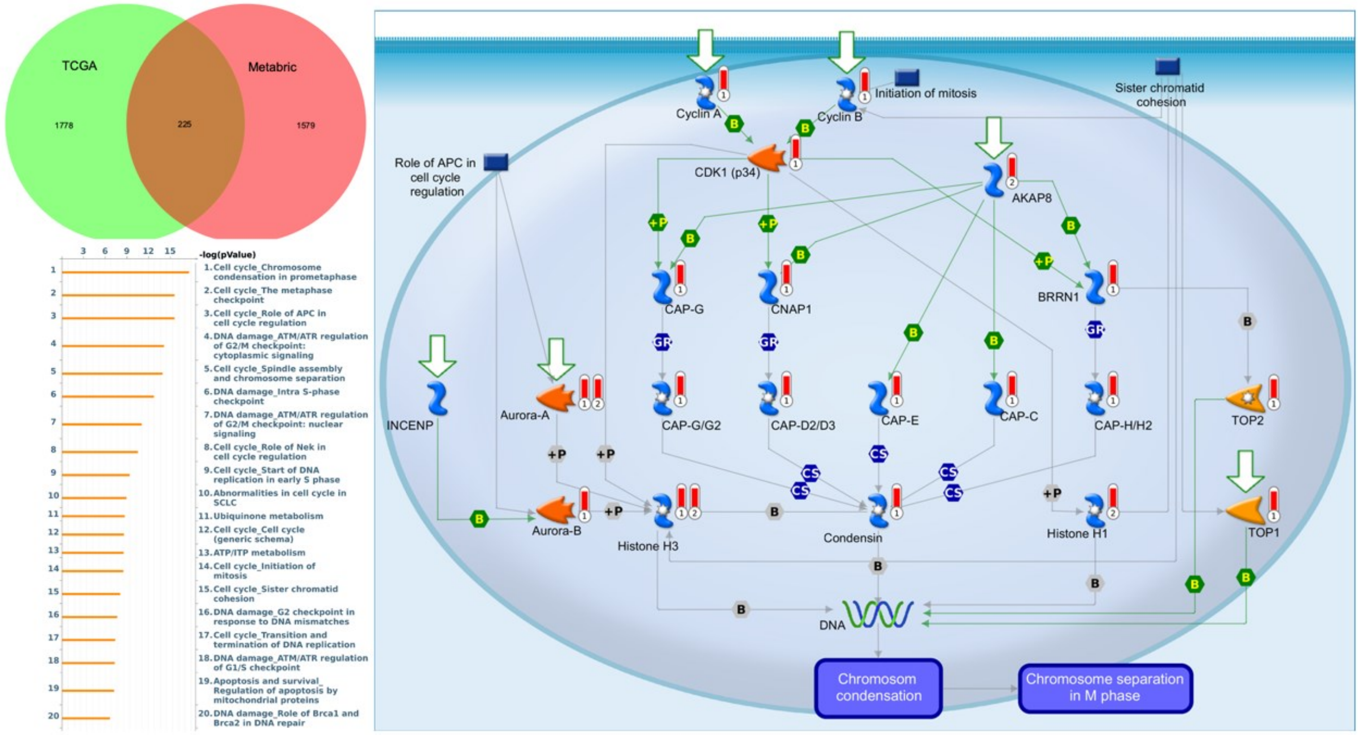

Supplementary Figure 6. MetaCore enrichment pathway analysis of genes co-expressed with cadherin 7 (*CDH7*). Top 10% of expression gene lists from both Metabric (1804 genes) and TCGA (2003 genes) were extracted. Overlapped (225) genes were integrated to implement pathway analysis, which formed a pathway list ordered by the -log *p*-value. "Cell cycle_Chromosome condensation in prometaphase" was at the top of the pathway list when performing the "biological process" analysis. The figure demonstrates the interactions between genes and proteins. Symbols represent proteins. Arrows depict protein interactions (green, activation; red, inhibition). Thermometer-like histograms indicate microarray gene expression (blue, down-regulation; red, up-regulation).

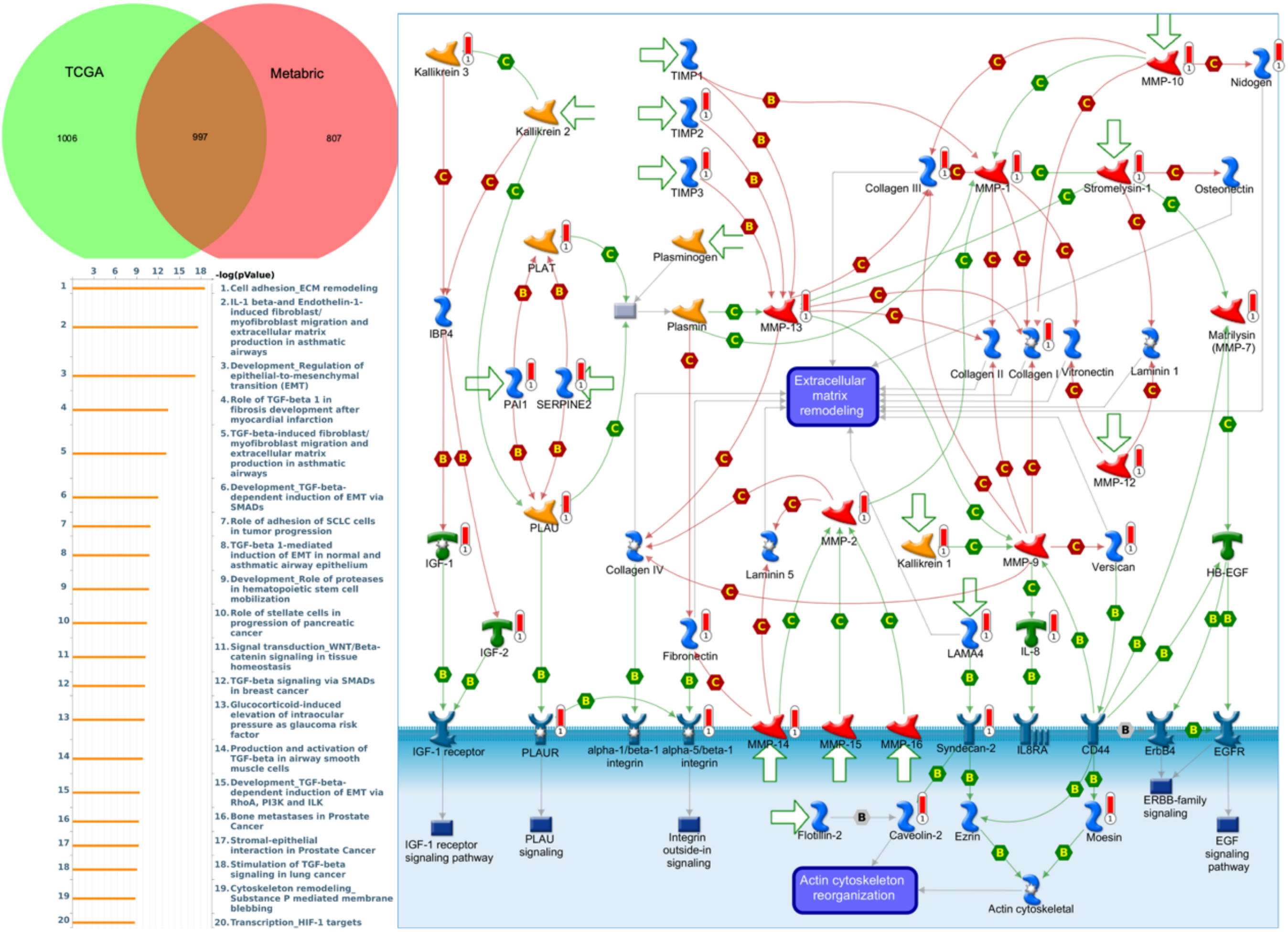

Supplementary Figure 7. MetaCore enrichment pathway analysis of genes co-expressed with cadherin 11 (*CDH11*). Top 10% of expression gene lists from both Metabric (1804 genes) and TCGA (2003 genes) were extracted. Overlapped (997) genes were integrated to implement pathway analysis, which formed a pathway list ordered by the -log *p*-value. "Cell adhesion_ECM remodeling" was at the top of the pathway list when performing the "biological process" analysis. The figure demonstrates the interactions between genes and proteins. Symbols represent proteins. Arrows depict protein interactions (green, activation; red, inhibition). Thermometer-like histograms indicate microarray gene expression (blue, down-regulation; red, up-regulation).

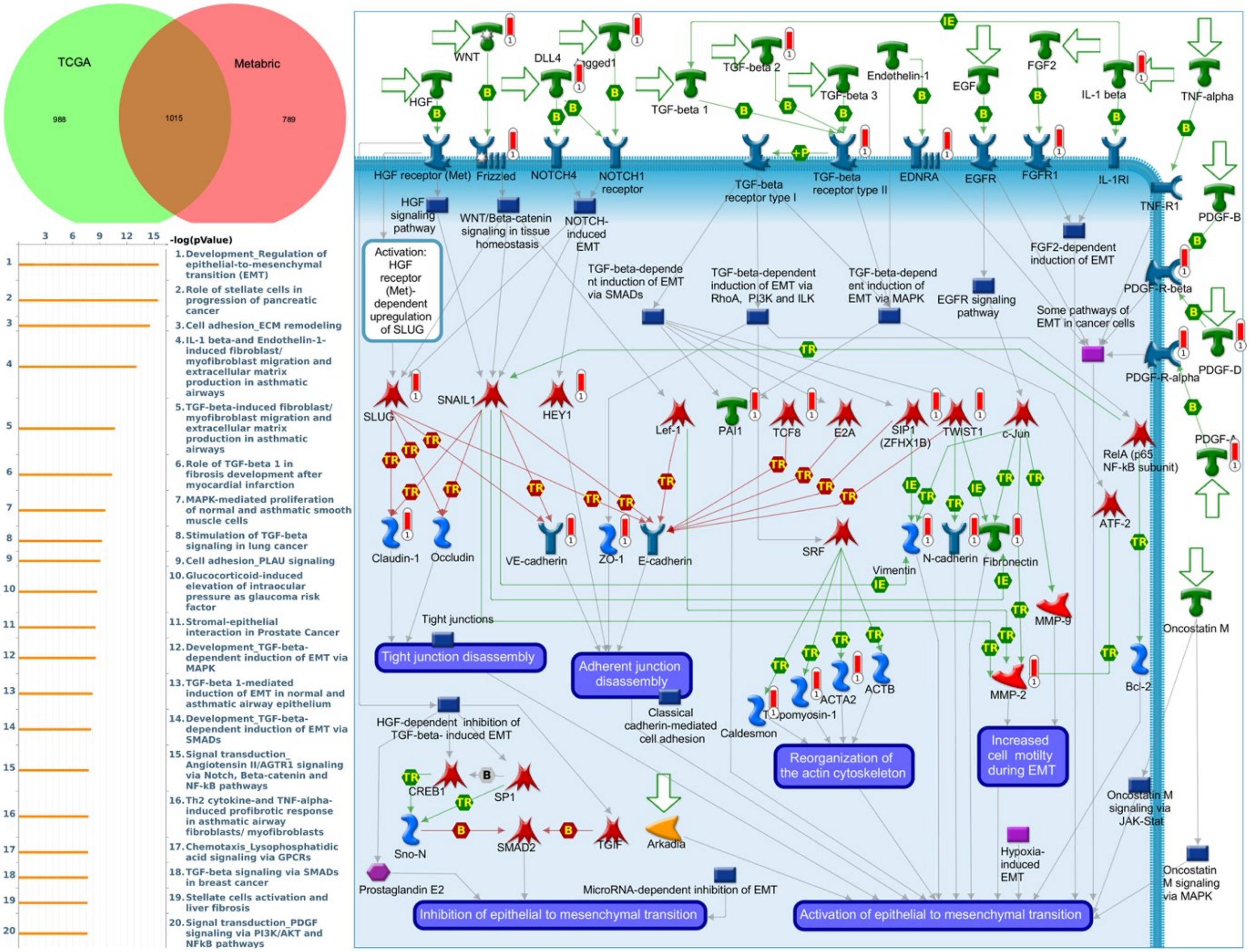

Supplementary Figure 8. MetaCore enrichment pathway analysis of genes co-expressed with cadherin 13 (*CDH13*). Top 10% of expression gene lists from both Metabric (1804 genes) and TCGA (2003 genes) were extracted. Overlapped (1015) genes were integrated to implement pathway analysis, which formed a pathway list ordered by the -log *p*-value. "Development_Regulation of epithelial-to-mesenchymal transition (EMT)" was at the top of the pathway list when performing the "biological process" analysis. The figure demonstrates the interactions between genes and proteins. Symbols represent proteins. Arrows depict protein interactions (green, activation; red, inhibition). Thermometer-like histograms indicate microarray gene expression (blue, down-regulation; red, up-regulation).

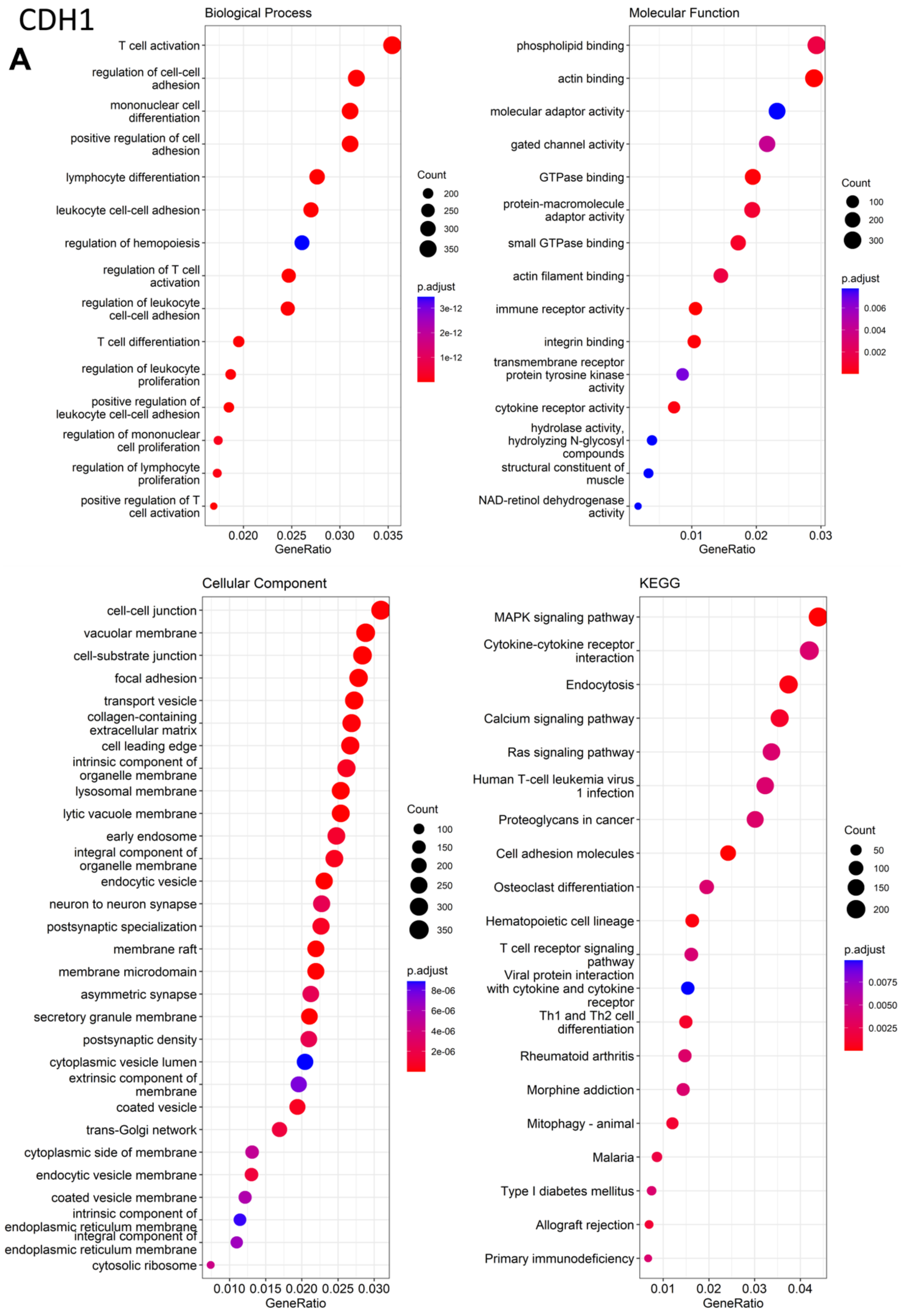
CDH1
A
Biological Process
T cell activation
regulation of cell-cell adhesion
mononuclear cell differentiation
positive regulation of cell adhesion
lymphocyte differentiation
leukocyte cell-cell adhesion
regulation of hemopoiesis
regulation of T cell activation
regulation of leukocyte cell-cell adhesion
T cell differentiation
regulation of leukocyte proliferation
positive regulation of leukocyte cell-cell adhesion
regulation of mononuclear cell proliferation
regulation of lymphocyte proliferation
positive regulation of T cell activation
0.020 0.025 0.030 0.035
GeneRatio
Count
200
250
300
350
p.adjust
3e-12
2e-12
1e-12
Molecular Function
phospholipid binding
actin binding
molecular adaptor activity
gated channel activity
GTPase binding
protein-macromolecule adaptor activity
small GTPase binding
actin filament binding
immune receptor activity
integrin binding
transmembrane receptor protein tyrosine kinase activity
cytokine receptor activity
hydrolase activity, hydrolyzing N-glycosyl compounds
structural constituent of muscle
NAD-retinol dehydrogenase activity
0.01 0.02 0.03
GeneRatio
Count
100
200
300
p.adjust
0.006
0.004
0.002
Cellular Component
cell-cell junction
vacuolar membrane
cell-substrate junction
focal adhesion
transport vesicle
collagen-containing extracellular matrix
cell leading edge
intrinsic component of organelle membrane
lysosomal membrane
lytic vacuole membrane
early endosome
integral component of organelle membrane
endocytic vesicle
neuron to neuron synapse
postsynaptic specialization
membrane raft
membrane microdomain
asymmetric synapse
secretory granule membrane
postsynaptic density
cytoplasmic vesicle lumen
extrinsic component of membrane
coated vesicle
trans-Golgi network
cytoplasmic side of membrane
endocytic vesicle membrane
coated vesicle membrane
intrinsic component of endoplasmic reticulum membrane
integral component of endoplasmic reticulum membrane
cytosolic ribosome
0.010 0.015 0.020 0.025 0.030
GeneRatio
Count
100
150
200
250
300
350
p.adjust
8e-06
6e-06
4e-06
2e-06
KEGG
MAPK signaling pathway
Cytokine-cytokine receptor interaction
Endocytosis
Calcium signaling pathway
Ras signaling pathway
Human T-cell leukemia virus 1 infection
Proteoglycans in cancer
Cell adhesion molecules
Osteoclast differentiation
Hematopoietic cell lineage
T cell receptor signaling pathway
Viral protein interaction with cytokine and cytokine receptor
Th1 and Th2 cell differentiation
Rheumatoid arthritis
Morphine addiction
Mitophagy - animal
Malaria
Type I diabetes mellitus
Allograft rejection
Primary immunodeficiency
0.01 0.02 0.03 0.04
GeneRatio
Count
50
100
150
200
p.adjust
0.0075
0.0050
0.0025

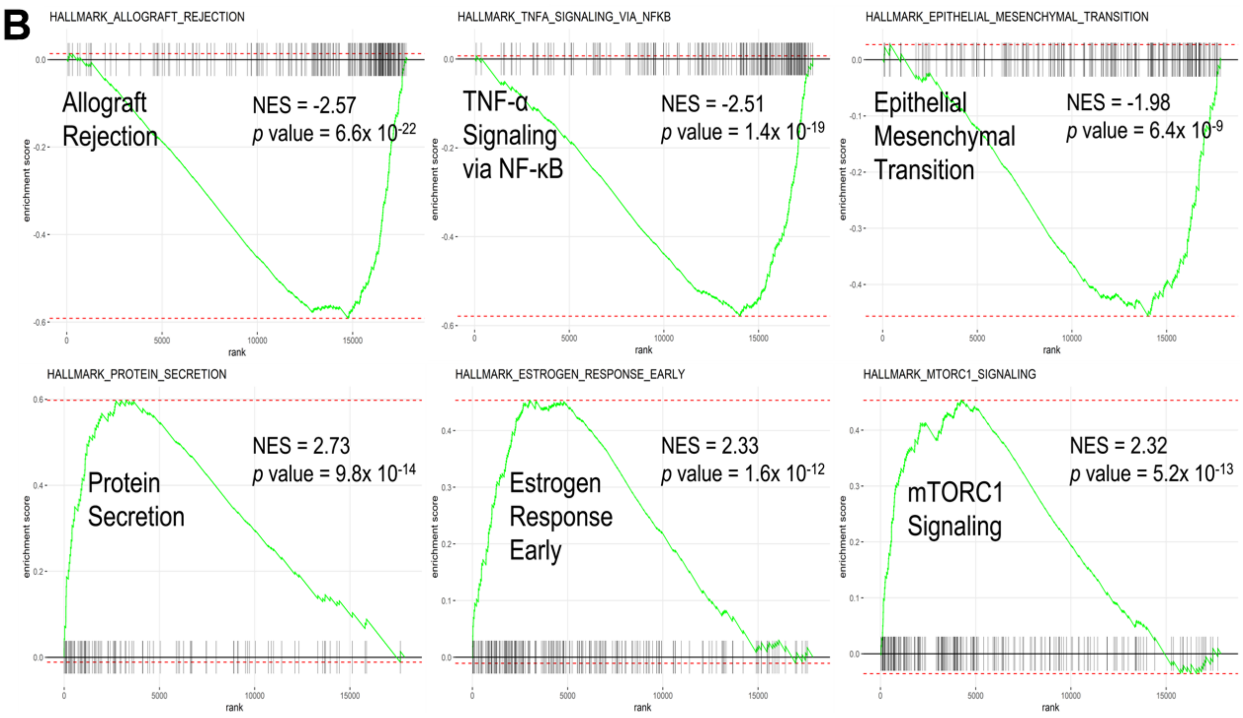

Supplementary Figure 9. (**A**, **B**) Gene Ontology (GO) analysis and Gene Set Enrichment Analysis (GSEA) based on genes co-expressed with *CDH1*. (**A**) Dot plots display biological processes, cellular components, molecular functions, and KEGG. The dot size is determined by the count of enriched genes in the pathway, and the color of the dots represents the significance of enrichment pathway. "clusterProfiler" package in R/Bioconductor was used to perform the GO analyses of dot plots. (**B**) Hallmark signaling pathway analysis of *CDH1* in breast cancer. Results of the analysis showed significant values of gene classes in the Hallmark database. Statistical significance was presented by *p* value, and the normalized enrichment score (NES) reflected the rank of gene classes.

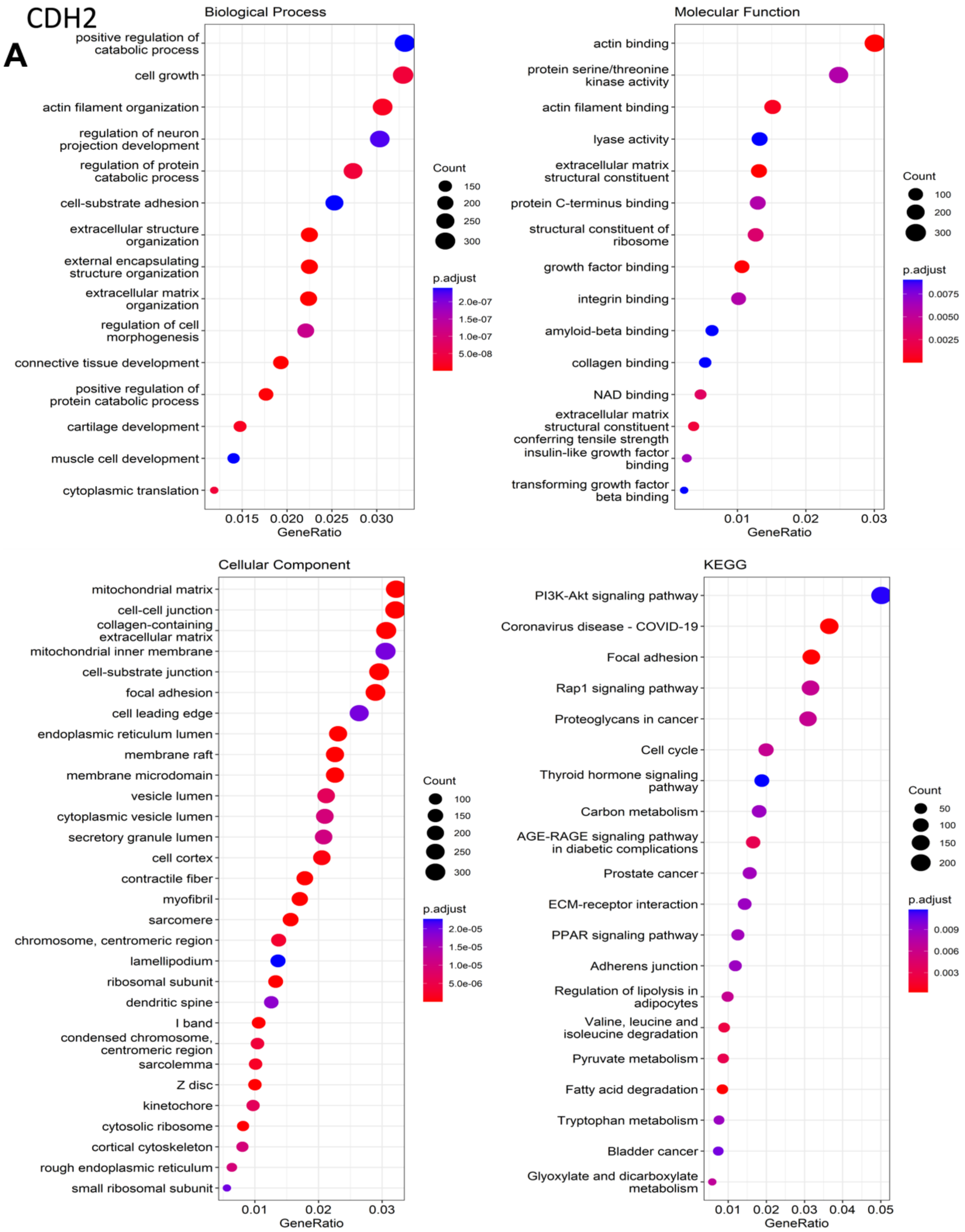

CDH2
A
Biological Process
positive regulation of catabolic process
cell growth
actin filament organization
regulation of neuron projection development
regulation of protein catabolic process
cell-substrate adhesion
extracellular structure organization
external encapsulating structure organization
extracellular matrix organization
regulation of cell morphogenesis
connective tissue development
positive regulation of protein catabolic process
cartilage development
muscle cell development
cytoplasmic translation
0.015 0.020 0.025 0.030
GeneRatio
Count
150
200
250
300
p.adjust
2.0e-07
1.5e-07
1.0e-07
5.0e-08
Molecular Function
actin binding
protein serine/threonine kinase activity
actin filament binding
lyase activity
extracellular matrix structural constituent
protein C-terminus binding
structural constituent of ribosome
growth factor binding
integrin binding
amyloid-beta binding
collagen binding
NAD binding
extracellular matrix structural constituent conferring tensile strength
insulin-like growth factor binding
transforming growth factor beta binding
0.01 0.02 0.03
GeneRatio
Count
100
200
300
p.adjust
0.0075
0.0050
0.0025
Cellular Component
mitochondrial matrix
cell-cell junction
collagen-containing extracellular matrix
mitochondrial inner membrane
cell-substrate junction
focal adhesion
cell leading edge
endoplasmic reticulum lumen
membrane raft
membrane microdomain
vesicle lumen
cytoplasmic vesicle lumen
secretory granule lumen
cell cortex
contractile fiber
myofibril
sarcomere
chromosome, centromeric region
lamellipodium
ribosomal subunit
dendritic spine
I band
condensed chromosome, centromeric region
sarcolemma
Z disc
kinetochore
cytosolic ribosome
cortical cytoskeleton
rough endoplasmic reticulum
small ribosomal subunit
0.01 0.02 0.03
GeneRatio
Count
100
150
200
250
300
p.adjust
2.0e-05
1.5e-05
1.0e-05
5.0e-06
KEGG
PI3K-Akt signaling pathway
Coronavirus disease - COVID-19
Focal adhesion
Rap1 signaling pathway
Proteoglycans in cancer
Cell cycle
Thyroid hormone signaling pathway
Carbon metabolism
AGE-RAGE signaling pathway in diabetic complications
Prostate cancer
ECM-receptor interaction
PPAR signaling pathway
Adherens junction
Regulation of lipolysis in adipocytes
Valine, leucine and isoleucine degradation
Pyruvate metabolism
Fatty acid degradation
Tryptophan metabolism
Bladder cancer
Glyoxylate and dicarboxylate metabolism
0.01 0.02 0.03 0.04 0.05
GeneRatio
Count
50
100
150
200
p.adjust
0.009
0.006
0.003

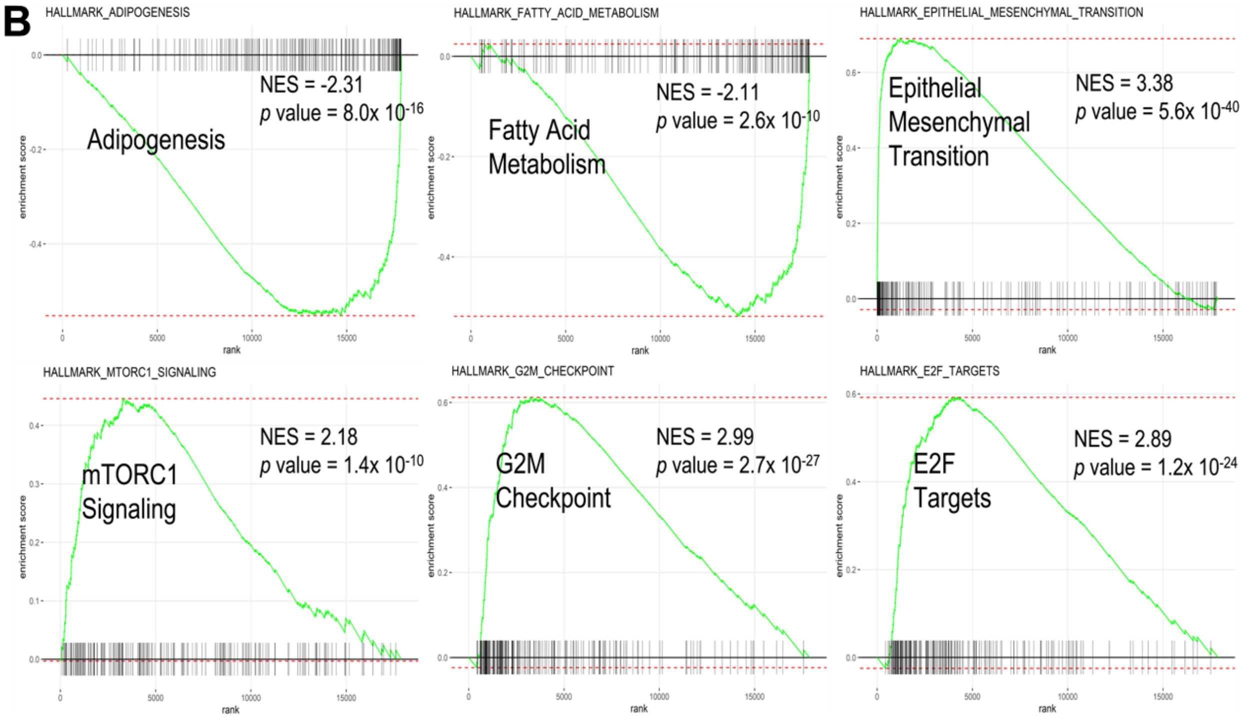

Supplementary Figure 10. (**A**, **B**) Gene Ontology (GO) analysis and Gene Set Enrichment Analysis (GSEA) based on genes co-expressed with *CDH2*. (**A**) Dot plots display biological processes, cellular components, molecular functions, and KEGG. The dot size is determined by the count of enriched genes in the pathway, and the color of the dots represents the significance of enrichment pathway. "clusterProfiler" package in R/Bioconductor was used to perform the GO analyses of dot plots. (**B**) Hallmark signaling pathway analysis of *CDH2* in breast cancer. Results of the analysis showed significant values of gene classes in the Hallmark database. Statistical significance was presented by *p* value, and the normalized enrichment score (NES) reflected the rank of gene classes.

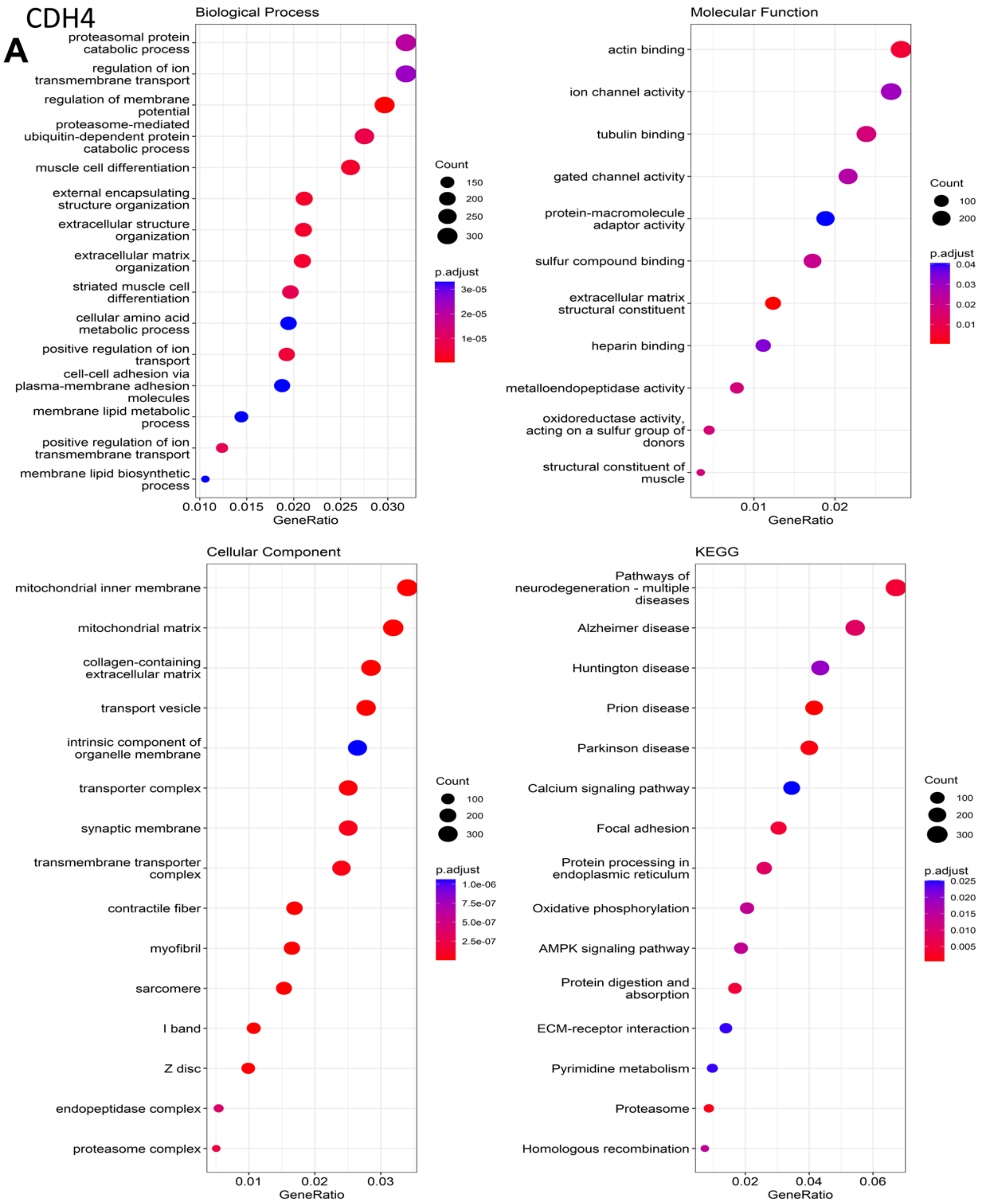
CDH4
A
Biological Process
proteasomal protein catabolic process
regulation of ion transmembrane transport
regulation of membrane potential
proteasome-mediated ubiquitin-dependent protein catabolic process
muscle cell differentiation
external encapsulating structure organization
extracellular structure organization
extracellular matrix organization
striated muscle cell differentiation
cellular amino acid metabolic process
positive regulation of ion transport
cell-cell adhesion via plasma-membrane adhesion molecules
membrane lipid metabolic process
positive regulation of ion transmembrane transport
membrane lipid biosynthetic process
0.010 0.015 0.020 0.025 0.030
GeneRatio
Count
150
200
250
300
p.adjust
3e-05
2e-05
1e-05
Molecular Function
actin binding
ion channel activity
tubulin binding
gated channel activity
protein-macromolecule adaptor activity
sulfur compound binding
extracellular matrix structural constituent
heparin binding
metalloendopeptidase activity
oxidoreductase activity, acting on a sulfur group of donors
structural constituent of muscle
0.01 0.02
GeneRatio
Count
100
200
p.adjust
0.04
0.03
0.02
0.01
Cellular Component
mitochondrial inner membrane
mitochondrial matrix
collagen-containing extracellular matrix
transport vesicle
intrinsic component of organelle membrane
transporter complex
synaptic membrane
transmembrane transporter complex
contractile fiber
myofibril
sarcomere
I band
Z disc
endopeptidase complex
proteasome complex
0.01 0.02 0.03
GeneRatio
Count
100
200
300
p.adjust
1.0e-06
7.5e-07
5.0e-07
2.5e-07
KEGG
Pathways of neurodegeneration - multiple diseases
Alzheimer disease
Huntington disease
Prion disease
Parkinson disease
Calcium signaling pathway
Focal adhesion
Protein processing in endoplasmic reticulum
Oxidative phosphorylation
AMPK signaling pathway
Protein digestion and absorption
ECM-receptor interaction
Pyrimidine metabolism
Proteasome
Homologous recombination
0.02 0.04 0.06
GeneRatio
Count
100
200
300
p.adjust
0.025
0.020
0.015
0.010
0.005

B

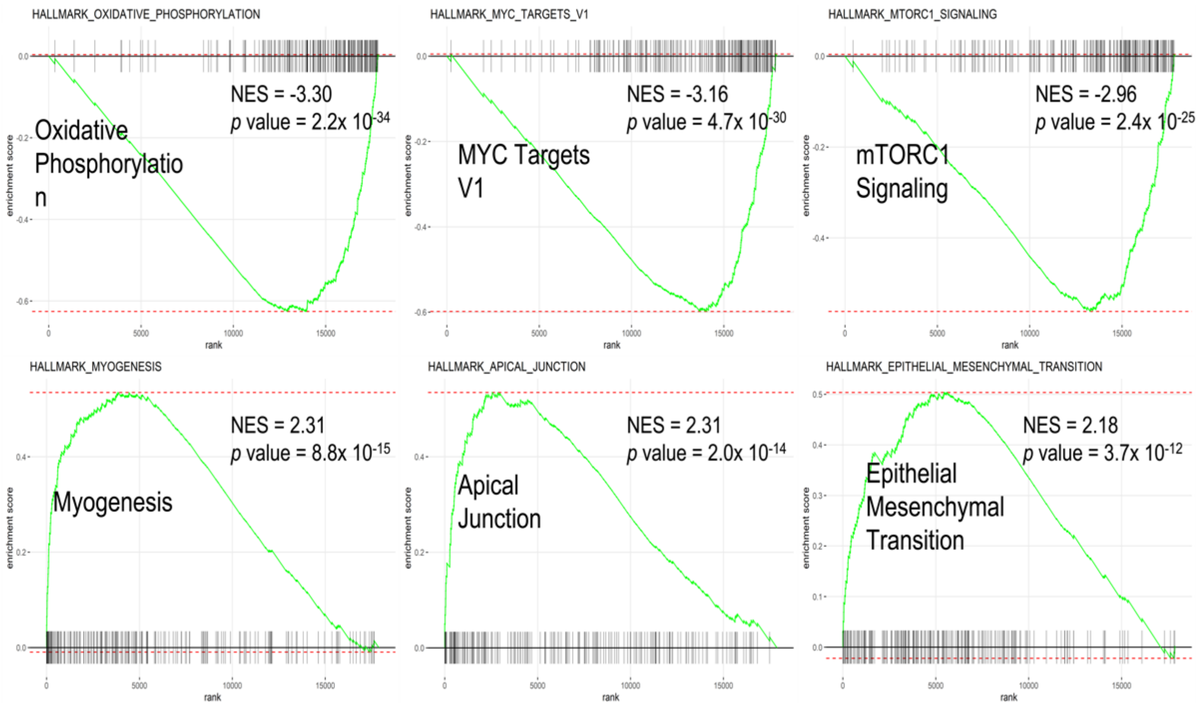

Supplementary Figure 11. (**A**, **B**) Gene Ontology (GO) analysis and Gene Set Enrichment Analysis (GSEA) based on genes co-expressed with *CDH4*. (**A**) Dot plots display biological processes, cellular components, molecular functions, and KEGG. The dot size is determined by the count of enriched genes in the pathway, and the color of the dots represents the significance of enrichment pathway. "clusterProfiler" package in R/Bioconductor was used to perform the GO analyses of dot plots. (**B**) Hallmark signaling pathway analysis of *CDH4* in breast cancer. Results of the analysis showed significant values of gene classes in the Hallmark database. Statistical significance was presented by *p* value, and the normalized enrichment score (NES) reflected the rank of gene classes.

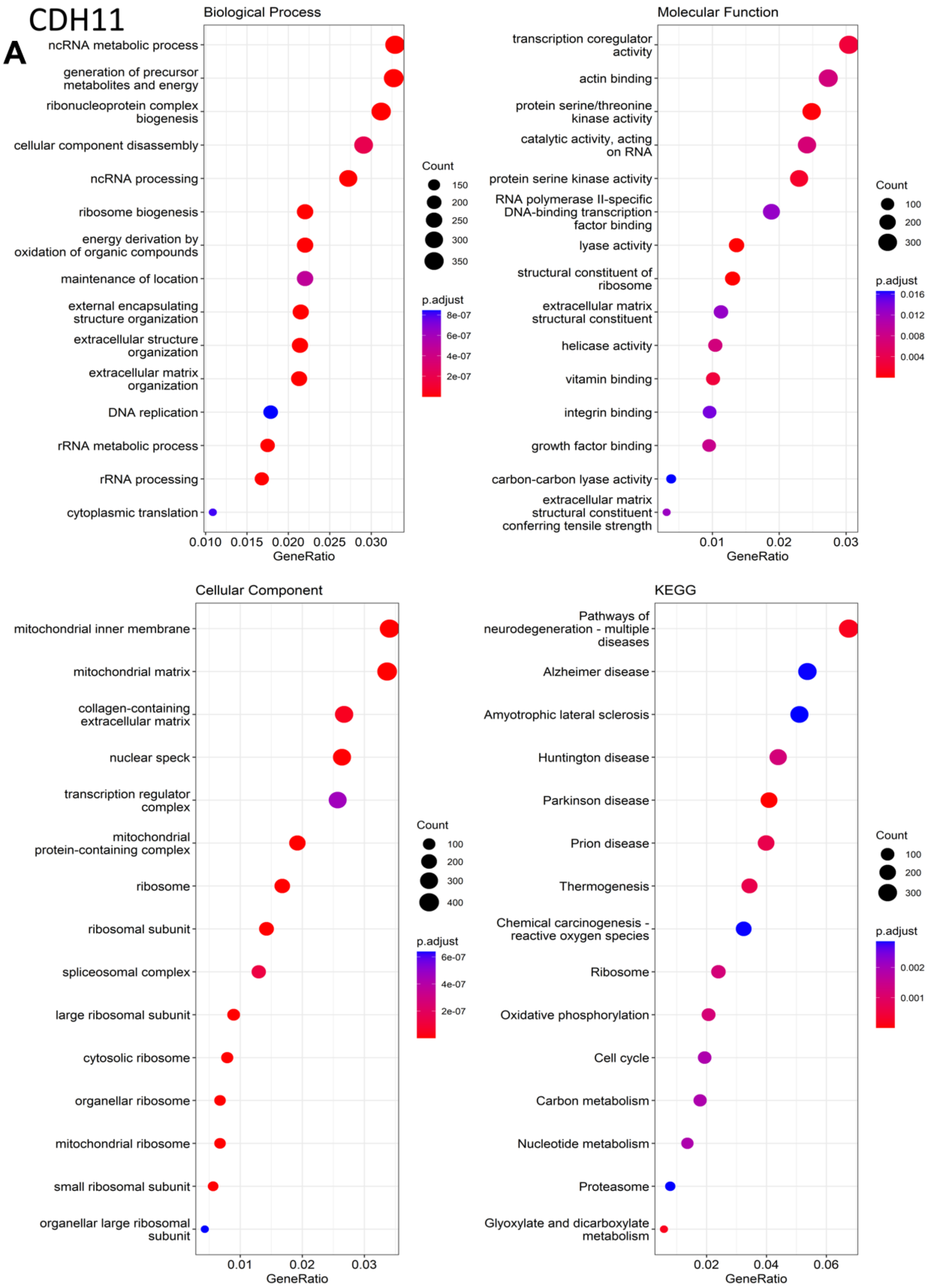
CDH11
A
Biological Process
ncRNA metabolic process
generation of precursor metabolites and energy
ribonucleoprotein complex biogenesis
cellular component disassembly
ncRNA processing
ribosome biogenesis
energy derivation by oxidation of organic compounds
maintenance of location
external encapsulating structure organization
extracellular structure organization
extracellular matrix organization
DNA replication
rRNA metabolic process
rRNA processing
cytoplasmic translation
0.010 0.015 0.020 0.025 0.030
GeneRatio
Count
150
200
250
300
350
p.adjust
8e-07
6e-07
4e-07
2e-07
Molecular Function
transcription coregulator activity
actin binding
protein serine/threonine kinase activity
catalytic activity, acting on RNA
protein serine kinase activity
RNA polymerase II-specific DNA-binding transcription factor binding
lyase activity
structural constituent of ribosome
extracellular matrix structural constituent
helicase activity
vitamin binding
integrin binding
growth factor binding
carbon-carbon lyase activity
extracellular matrix structural constituent conferring tensile strength
0.01 0.02 0.03
GeneRatio
Count
100
200
300
p.adjust
0.016
0.012
0.008
0.004
Cellular Component
mitochondrial inner membrane
mitochondrial matrix
collagen-containing extracellular matrix
nuclear speck
transcription regulator complex
mitochondrial protein-containing complex
ribosome
ribosomal subunit
spliceosomal complex
large ribosomal subunit
cytosolic ribosome
organellar ribosome
mitochondrial ribosome
small ribosomal subunit
organellar large ribosomal subunit
0.01 0.02 0.03
GeneRatio
Count
100
200
300
400
p.adjust
6e-07
4e-07
2e-07
KEGG
Pathways of neurodegeneration - multiple diseases
Alzheimer disease
Amyotrophic lateral sclerosis
Huntington disease
Parkinson disease
Prion disease
Thermogenesis
Chemical carcinogenesis - reactive oxygen species
Ribosome
Oxidative phosphorylation
Cell cycle
Carbon metabolism
Nucleotide metabolism
Proteasome
Glyoxylate and dicarboxylate metabolism
0.02 0.04 0.06
GeneRatio
Count
100
200
300
p.adjust
0.002
0.001

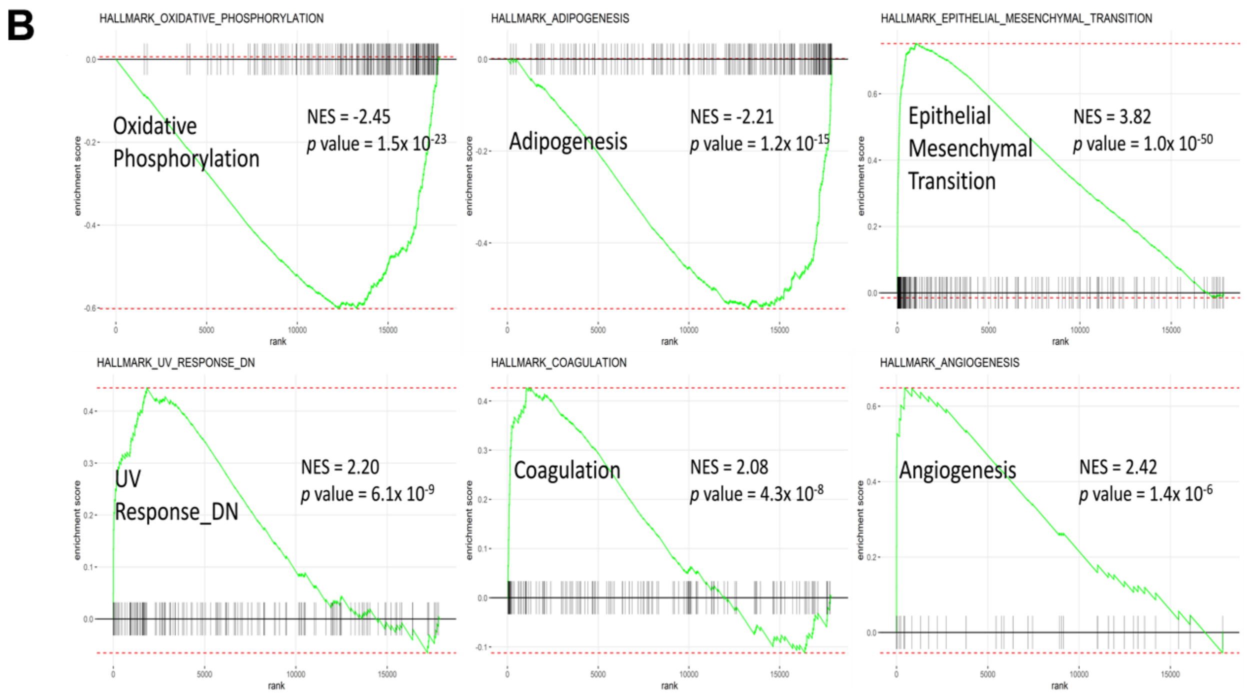

Supplementary Figure 12. (**A**, **B**) Gene Ontology (GO) analysis and Gene Set Enrichment Analysis (GSEA) based on genes co-expressed with *CDH11*. (**A**) Dot plots display biological processes, cellular components, molecular functions, and KEGG. The dot size is determined by the count of enriched genes in the pathway, and the color of the dots represents the significance of enrichment pathway. "clusterProfiler" package in R/Bioconductor was used to perform the GO analyses of dot plots. (**B**) Hallmark signaling pathway analysis of *CDH11* in breast cancer. Results of the analysis showed significant values of gene classes in the Hallmark database. Statistical significance was presented by *p* value, and the normalized enrichment score (NES) reflected the rank of gene classes.

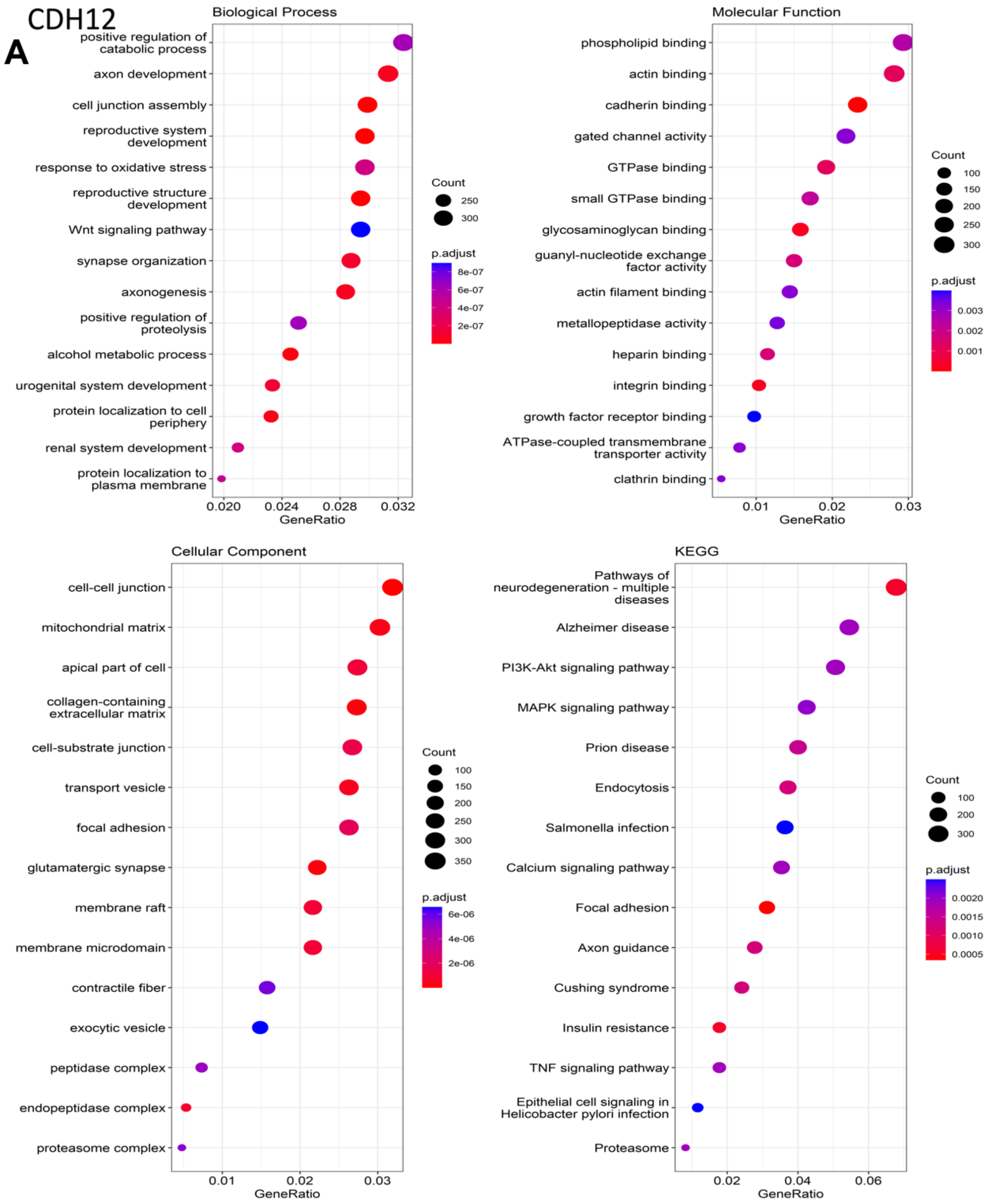

CDH12
A
Biological Process
positive regulation of catabolic process
axon development
cell junction assembly
reproductive system development
response to oxidative stress
reproductive structure development
Wnt signaling pathway
synapse organization
axonogenesis
positive regulation of proteolysis
alcohol metabolic process
urogenital system development
protein localization to cell periphery
renal system development
protein localization to plasma membrane
GeneRatio
Count
p.adjust
Molecular Function
phospholipid binding
actin binding
cadherin binding
gated channel activity
GTPase binding
small GTPase binding
glycosaminoglycan binding
guanyl-nucleotide exchange factor activity
actin filament binding
metallopeptidase activity
heparin binding
integrin binding
growth factor receptor binding
ATPase-coupled transmembrane transporter activity
clathrin binding
Cellular Component
cell-cell junction
mitochondrial matrix
apical part of cell
collagen-containing extracellular matrix
cell-substrate junction
transport vesicle
focal adhesion
glutamatergic synapse
membrane raft
membrane microdomain
contractile fiber
exocytic vesicle
peptidase complex
endopeptidase complex
proteasome complex
KEGG
Pathways of neurodegeneration - multiple diseases
Alzheimer disease
PI3K-Akt signaling pathway
MAPK signaling pathway
Prion disease
Endocytosis
Salmonella infection
Calcium signaling pathway
Focal adhesion
Axon guidance
Cushing syndrome
Insulin resistance
TNF signaling pathway
Epithelial cell signaling in Helicobacter pylori infection
Proteasome

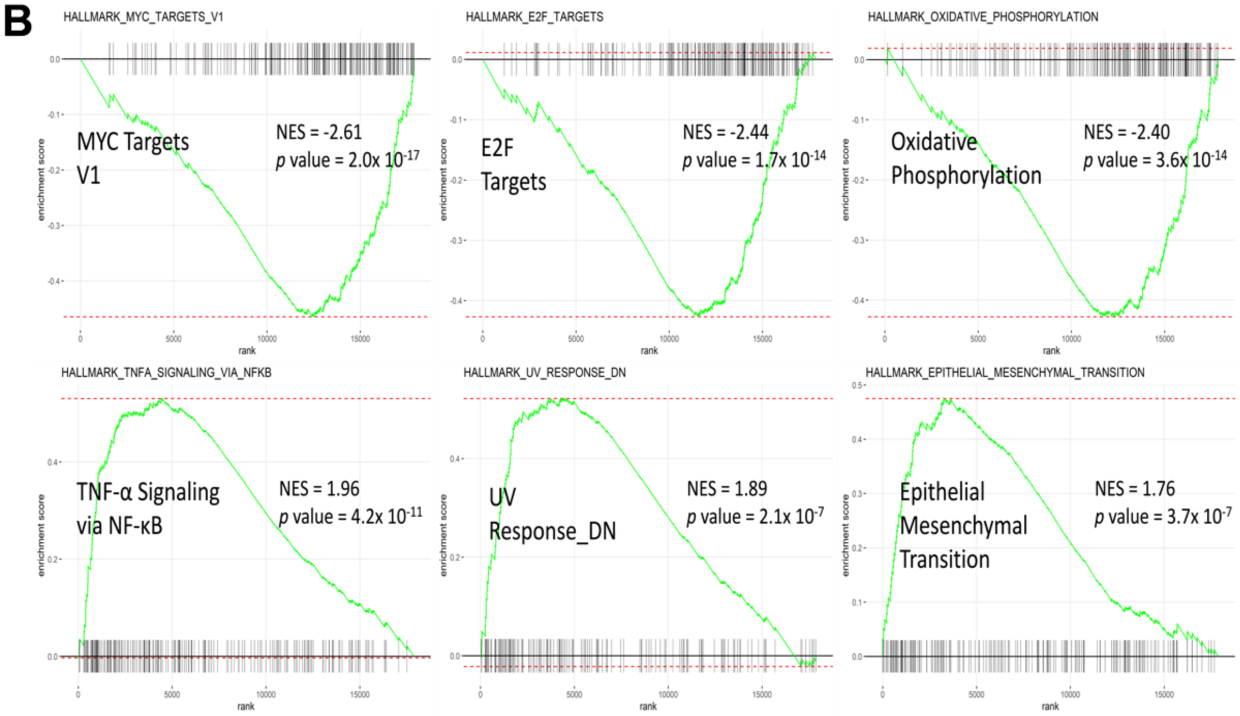

Supplementary Figure 13. (**A**, **B**) Gene Ontology (GO) analysis and Gene Set Enrichment Analysis (GSEA) based on genes co-expressed with *CDH12*. (**A**) Dot plots display biological processes, cellular components, molecular functions, and KEGG. The dot size is determined by the count of enriched genes in the pathway, and the color of the dots represents the significance of enrichment pathway. "clusterProfiler" package in R/Bioconductor was used to perform the GO analyses of dot plots. (**B**) Hallmark signaling pathway analysis of *CDH12* in breast cancer. Results of the analysis showed significant values of gene classes in the Hallmark database. Statistical significance was presented by *p* value, and the normalized enrichment score (NES) reflected the rank of gene classes.

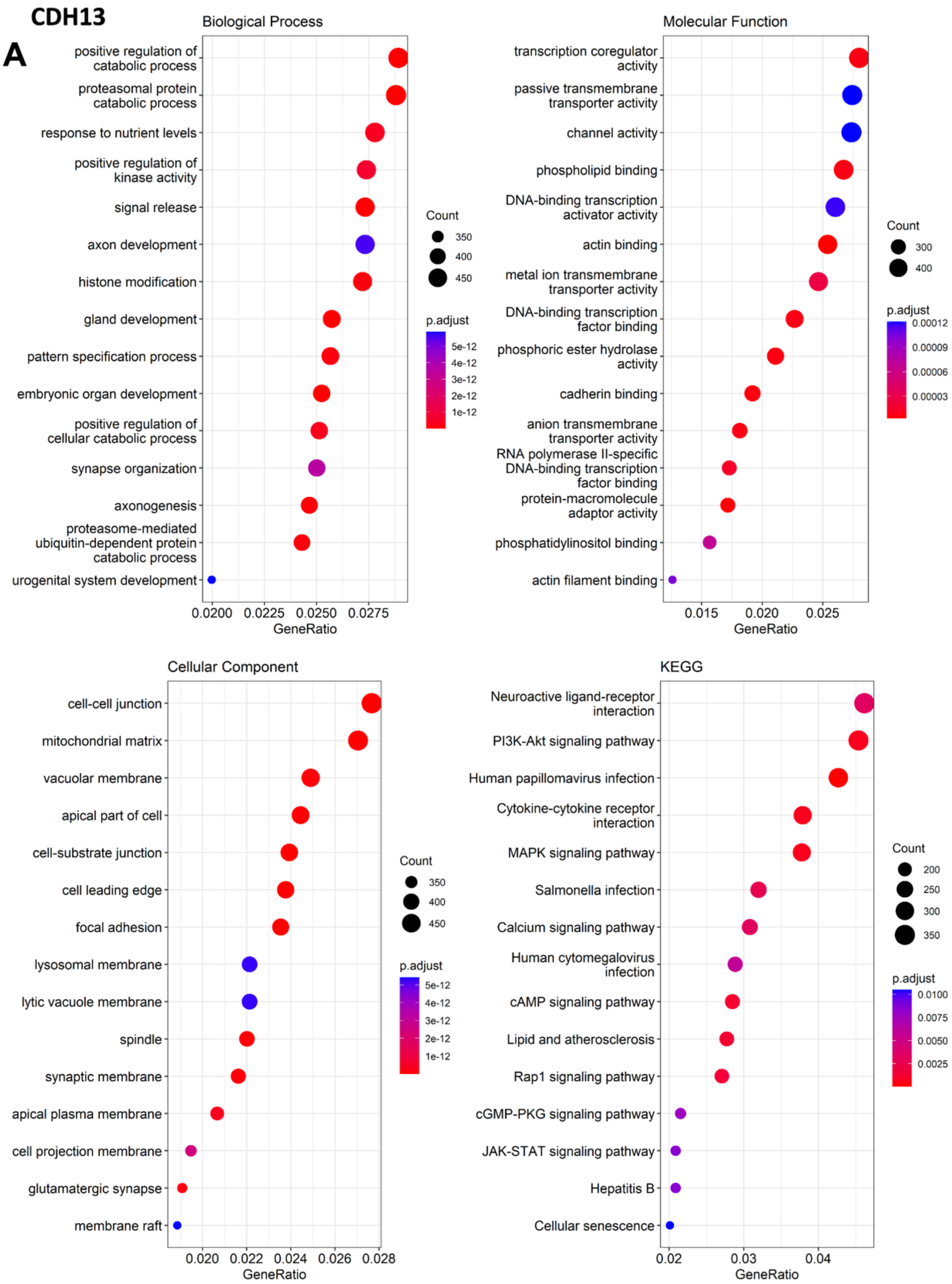
CDH13
A
Biological Process
positive regulation of catabolic process
proteasomal protein catabolic process
response to nutrient levels
positive regulation of kinase activity
signal release
axon development
histone modification
gland development
pattern specification process
embryonic organ development
positive regulation of cellular catabolic process
synapse organization
axonogenesis
proteasome-mediated ubiquitin-dependent protein catabolic process
urogenital system development
0.0200 0.0225 0.0250 0.0275
GeneRatio
Count
350
400
450
p.adjust
5e-12
4e-12
3e-12
2e-12
1e-12
Molecular Function
transcription coregulator activity
passive transmembrane transporter activity
channel activity
phospholipid binding
DNA-binding transcription activator activity
actin binding
metal ion transmembrane transporter activity
DNA-binding transcription factor binding
phosphoric ester hydrolase activity
cadherin binding
anion transmembrane transporter activity
RNA polymerase II-specific DNA-binding transcription factor binding
protein-macromolecule adaptor activity
phosphatidylinositol binding
actin filament binding
0.015 0.020 0.025
GeneRatio
Count
300
400
p.adjust
0.00012
0.00009
0.00006
0.00003
Cellular Component
cell-cell junction
mitochondrial matrix
vacuolar membrane
apical part of cell
cell-substrate junction
cell leading edge
focal adhesion
lysosomal membrane
lytic vacuole membrane
spindle
synaptic membrane
apical plasma membrane
cell projection membrane
glutamatergic synapse
membrane raft
0.020 0.022 0.024 0.026 0.028
GeneRatio
Count
350
400
450
p.adjust
5e-12
4e-12
3e-12
2e-12
1e-12
KEGG
Neuroactive ligand-receptor interaction
PI3K-Akt signaling pathway
Human papillomavirus infection
Cytokine-cytokine receptor interaction
MAPK signaling pathway
Salmonella infection
Calcium signaling pathway
Human cytomegalovirus infection
cAMP signaling pathway
Lipid and atherosclerosis
Rap1 signaling pathway
cGMP-PKG signaling pathway
JAK-STAT signaling pathway
Hepatitis B
Cellular senescence
0.02 0.03 0.04
GeneRatio
Count
200
250
300
350
p.adjust
0.0100
0.0075
0.0050
0.0025

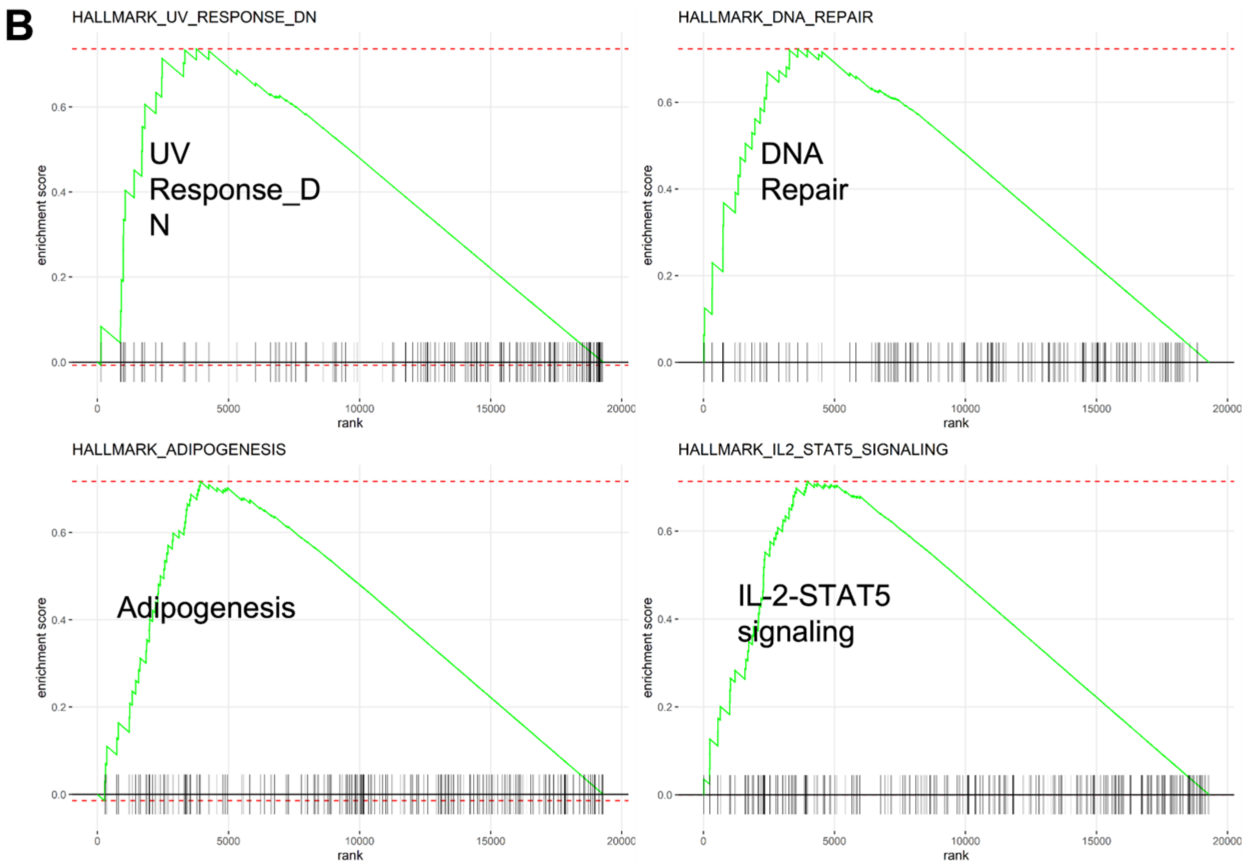

Supplementary Figure 14. (**A**, **B**) Gene Ontology (GO) analysis and Gene Set Enrichment Analysis (GSEA) based on genes co-expressed with *CDH13*. (**A**) Dot plots display biological processes, cellular components, molecular functions, and KEGG. The dot size is determined by the count of enriched genes in the pathway, and the color of the dots represents the significance of enrichment pathway. "clusterProfiler" package in R/Bioconductor was used to perform the GO analyses of dot plots. (**B**) Hallmark signaling pathway analysis of *CDH13* in breast cancer. Results of the analysis showed significant values of gene classes in the Hallmark database. Statistical significance was presented by *p* value, and the normalized enrichment score (NES) reflected the rank of gene classes.

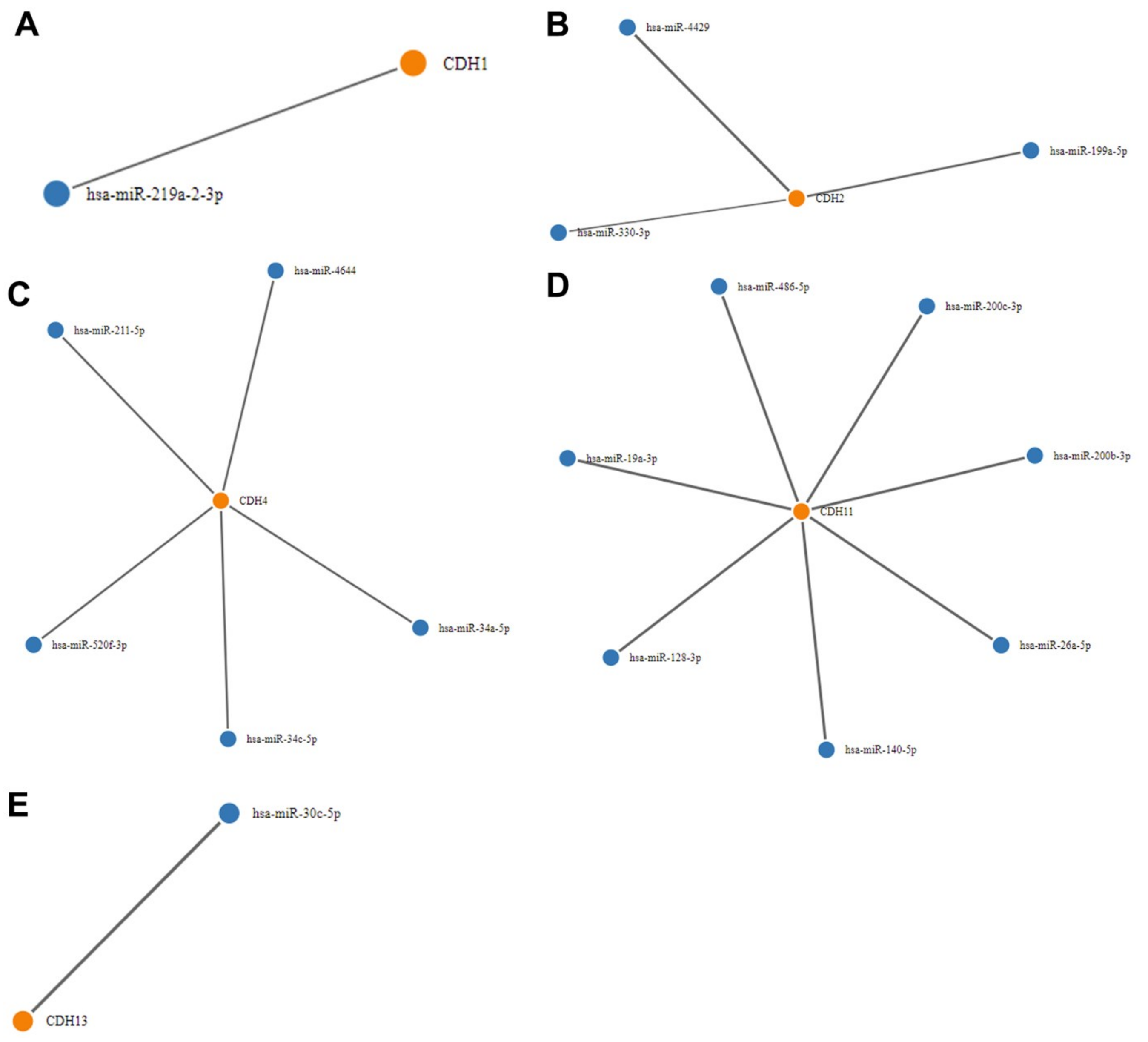

Supplementary Figure 15. Analysis of micro-(mi)RNA networks with *CDH1/2/4/11/13* in breast cancer. (**A**) *CDH1* demonstrated network interaction with hsa-miR-219a-2-3p in breast cancer development; (**B**) *CDH2* demonstrated network interaction with hsa-miR-330-3p, has-miR-4429, and hsa-miR-199a-5p; (**C**) *CDH4* demonstrated network interaction with hsa-miR-4644, hsa-miR-211-5p, hsa-miR-520f-3p, hsa-miR-34e-5p, and hsa-miR-34a-5p; (**D**) *CDH11* demonstrated network interaction with hsa-miR-486-5p, hsa-miR-200c-3p, hsa-miR-200b-3p, hsa-miR-26a-5p, hsa-miR-140-5p, hsa-miR-128-3p, and hsa-miR-19a-3p; (**E**) *CDH13* demonstrated network interaction with hsa-miR-30c-5p.

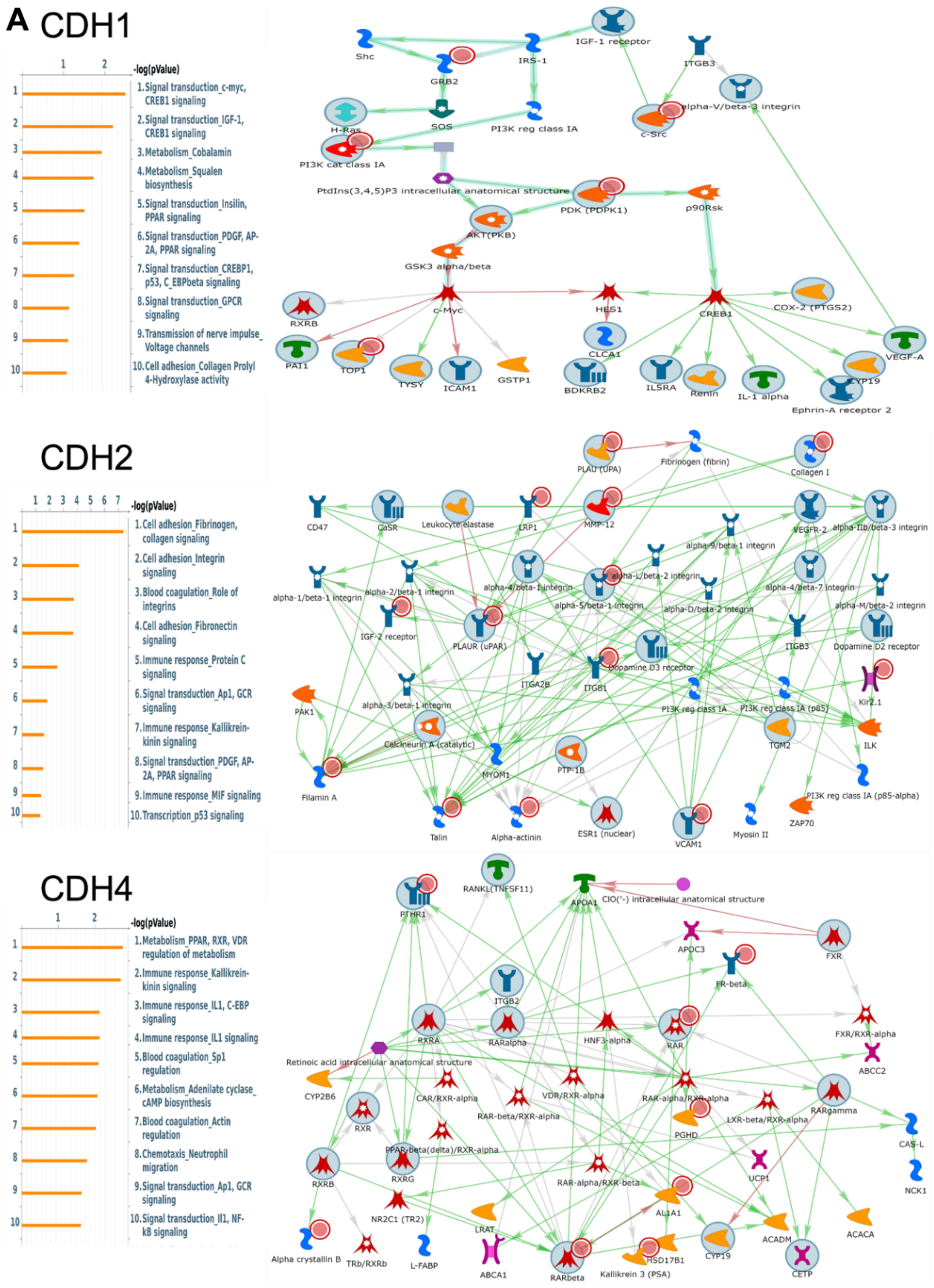

A CDH1
-log(pValue)
1. Signal transduction_c-myc, CREB1 signaling
2. Signal transduction_IGF-1, CREB1 signaling
3. Metabolism_Cobalamin
4. Metabolism_Squalen biosynthesis
5. Signal transduction_Insilin, PPAR signaling
6. Signal transduction_PDGF, AP-2A, PPAR signaling
7. Signal transduction_CREBP1, p53, C_EBPbeta signaling
8. Signal transduction_GPCR signaling
9. Transmission of nerve impulse_Voltage channels
10. Cell adhesion_Collagen Prolyl 4-Hydroxylase activity
Shc
IRS-1
IGF-1 receptor
GRB2
ITGB3
alpha-V/beta-3 integrin
H-Ras
SOS
PI3K reg class IA
c-Src
PI3K cat class IA
PtdIns(3,4,5)P3 intracellular anatomical structure
PDK (PDPK1)
p90Rsk
AKT(PKB)
GSK3 alpha/beta
RXRB
c-Myc
HES1
CREB1
COX-2 (PTGS2)
PAI1
TOP1
TYSY
ICAM1
GSTP1
CLCA1
BDKRB2
IL5RA
Renin
IL-1 alpha
CYP19
VEGF-A
Ephrin-A receptor 2
CDH2
-log(pValue)
1. Cell adhesion_Fibrinogen, collagen signaling
2. Cell adhesion_Integrin signaling
3. Blood coagulation_Role of integrins
4. Cell adhesion_Fibronectin signaling
5. Immune response_Protein C signaling
6. Signal transduction_Ap1, GCR signaling
7. Immune response_Kallikrein-kinin signaling
8. Signal transduction_PDGF, AP-2A, PPAR signaling
9. Immune response_MIF signaling
10. Transcription_p53 signaling
PLAU (UPA)
Fibrinogen (fibrin)
Collagen I
CD47
CaSR
Leukocyte elastase
LRP1
MMP-12
VEGFR-2
alpha-IIb/beta-3 integrin
alpha-9/beta-1 integrin
alpha-1/beta-1 integrin
alpha-2/beta-1 integrin
alpha-4/beta-1 integrin
alpha-L/beta-2 integrin
alpha-4/beta-7 integrin
alpha-5/beta-1 integrin
alpha-D/beta-2 integrin
alpha-M/beta-2 integrin
IGF-2 receptor
PLAUR (uPAR)
ITGB3
Dopamine D2 receptor
ITGA2B
ITGB1
Dopamine D3 receptor
PAK1
alpha-3/beta-1 integrin
PI3K reg class IA
PI3K reg class IA (p85)
Kir2.1
Calcineurin A (catalytic)
TGM2
ILK
MYOM1
PTP-1B
Filamin A
PI3K reg class IA (p85-alpha)
ZAP70
Talin
Alpha-actinin
ESR1 (nuclear)
VCAM1
Myosin II
CDH4
-log(pValue)
1. Metabolism_PPAR, RXR, VDR regulation of metabolism
2. Immune response_Kallikrein-kinin signaling
3. Immune response_IL1, C-EBP signaling
4. Immune response_IL1 signaling
5. Blood coagulation_Sp1 regulation
6. Metabolism_Adenilate cyclase_cAMP biosynthesis
7. Blood coagulation_Actin regulation
8. Chemotaxis_Neutrophil migration
9. Signal transduction_Ap1, GCR signaling
10. Signal transduction_Il1, NF-kB signaling
PTHR1
RANKL(TNFSF11)
APOA1
ClO('-) intracellular anatomical structure
APOC3
FXR
FR-beta
ITGB2
RXRA
RARalpha
HNF3-alpha
RAR
FXR/RXR-alpha
ABCC2
Retinoic acid intracellular anatomical structure
CYP2B6
CAR/RXR-alpha
VDR/RXR-alpha
RAR-alpha/RXR-alpha
RAR-beta/RXR-alpha
LXR-beta/RXR-alpha
RARgamma
PGHD
RXR
PPAR-beta(delta)/RXR-alpha
CAS-L
UCP1
RXRB
RXRG
RAR-alpha/RXR-beta
NCK1
NR2C1 (TR2)
AL1A1
LRAT
ACADM
ACACA
Alpha crystallin B
TRb/RXRb
L-FABP
ABCA1
RARbeta
Kallikrein 3 (PSA)
HSD17B1
CYP19
CETP

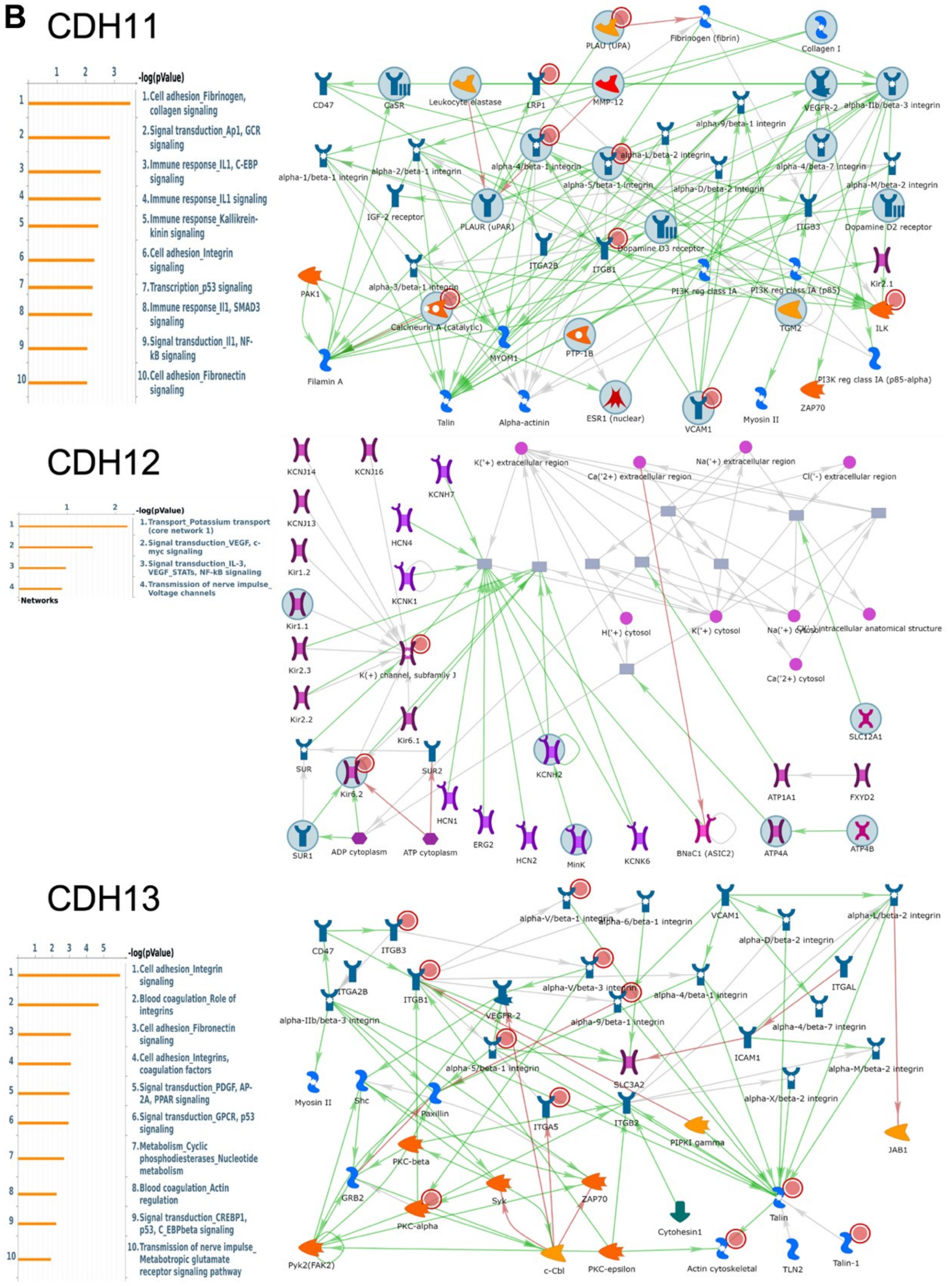

Supplementary Figure 16. (**A**, **B**) Drug target networks analysis of genes co-expressed with cadherin (*CDH)* 1/2/4/11/12/13. Genes co-expressed with CDH1/2/4/11/12/13 were integrated to implement drug target networks analysis, which formed a pathway list ordered by the -log *p*-value on the left. (**A**) "Signal transduction_c-myc, CREB1 signaling" was at the top of the drug target of CDH1; "Cell adhesion_Fibrinogen, collagen signaling" was at the top of the drug target of CDH2; "Metabolism_PPAR, RXR, VDR regulation of metabolism" was at the top of the drug target of CDH4; (**B**) "Cell adhesion_Fibrinogen, collagen signaling" was at the top of the drug target of CDH11; "Transport_Potassium transport (core work 1)" was at the top of the drug target of CDH12; "Cell adhesion_Intergrin signaling" was at the top of the drug target of CDH13. The figure demonstrates the interactions between genes and proteins. Symbols represent proteins. Arrows depict protein interactions (green, activation; red, inhibition).

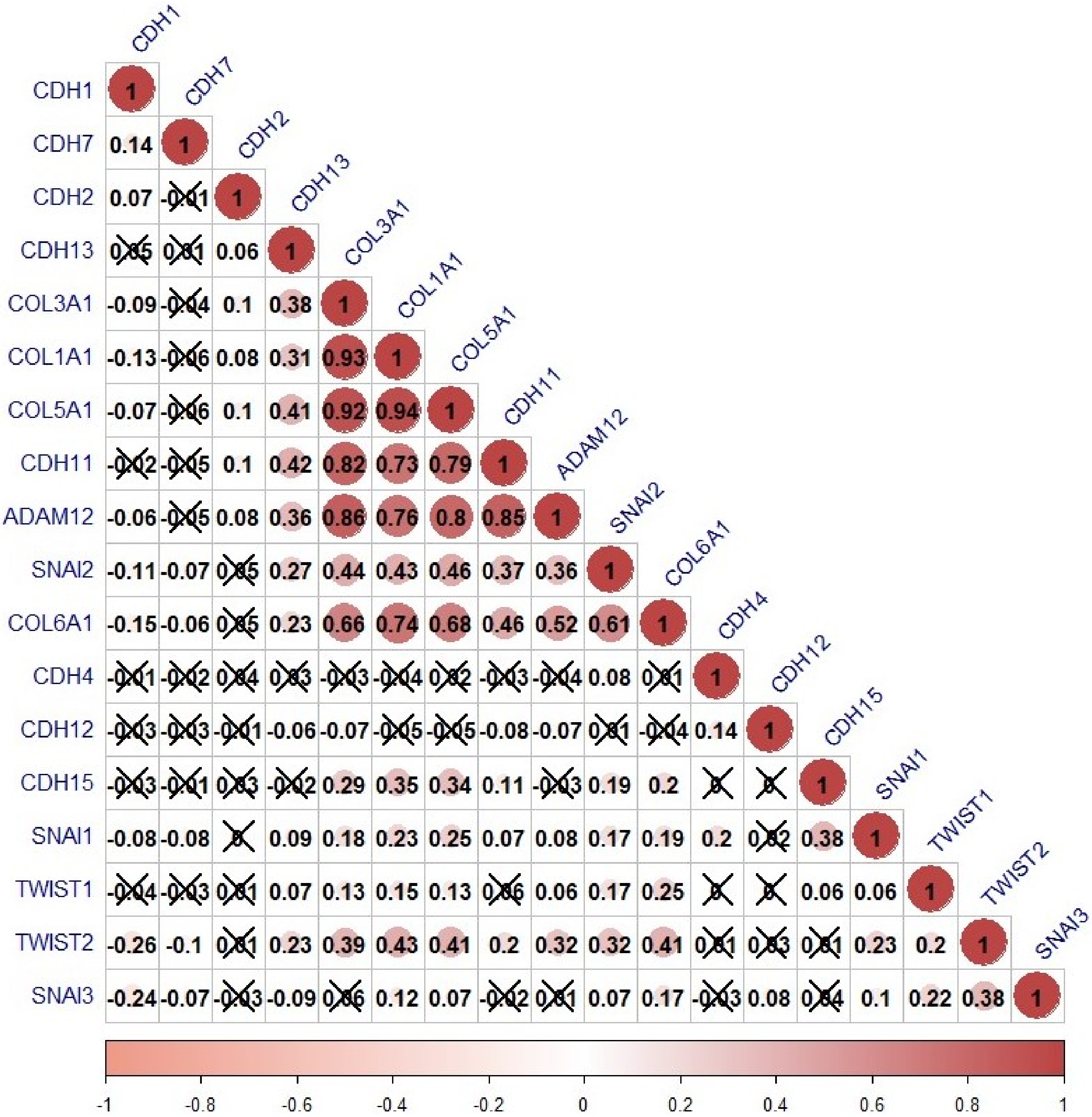

Supplementary Figure 17. Correlations between cadherin (*CDH*) family and epithelial-mesenchymal transition (EMT)-regulated genes in breast cancer using TCGA dataset. The symmetric correlation matrix was created by the “corrplot” R package. The color represents the degree of pairwise correlation regarding Spearman’s rank correlation coefficient. Darker red and larger dot size mean stronger positive correlation, while orange indicates stronger negative correlation. The cross symbols represent non-significant correlation coefficient values (*p* value > 0.01).
