# Supplementary material for: Comprehensive analysis of prognostic significance of cadherin (CDH) gene family in breast cancer
Source: Aging (Albany NY). 2022 Oct 25;14(20):8498–567. doi: 10.18632/aging.204357 (PMC9648792; doi:10.18632/aging.204357)
Supplement: Supplementary Tables [file aging-14-204357-s002.pdf]

SUPPLEMENTARY TABLES

Supplementary Table 1A. Univariate Cox regression analysis of CDH1/2/4/11/12/13 and age, gender, tumor stage with overall survival in breast cancer (BRCA) patients.

Univariate	BRCA			
	Overall survival			
	Coefficient	HR	(95% CI)	*p* value
Age	0.036	1.037	(1.023-1.050)	0.000***
gender male	-0.49	0.613	(0.084-4.450)	0.628
stage2	0.668	1.951	(1.117-3.405)	0.019*
stage3	1.374	3.95	(2.189-7.126)	0.000***
stage4	2.718	15.143	(7.074-32.417)	0.000***
CDH1	0.014	1.014	(0.913-1.127)	0.792
CDH2	0.101	1.106	(0.944-1.297)	0.213
CDH4	-0.066	0.936	(0.609-1.439)	0.764
CDH11	-0.025	0.976	(0.828-1.150)	0.769
CDH12	-0.291	0.747	(0.431-1.295)	0.299
CDH13	0.264	1.302	(1.053-1.611)	0.015*

The coefficient represents the regression coefficient. HR represents the hazard ratio, and 95% confidential interval are showed in (95% CI).

Supplementary Table 1B. Univariate Cox regression analysis of CDH1/2/4/11/12/13 and age, gender, tumor stage with overall survival in molecular subtype-Luminal of breast cancer (BRCA) patients.

Univariate	BRCA-luminal			
	Overall survival			
	Coefficient	HR	(95% CI)	*p* value
Age	0.039	1.039	(1.020-1.059)	0.000***
gender male	-15.811	0	(0.000-Inf)	0.995
stage2	0.559	1.749	(0.899-3.403)	0.1
stage3	1.106	3.021	(1.448-6.303)	0.003**
stage4	2.223	9.232	(3.471-24.552)	0.000***
CDH1	0.069	1.071	(0.917-1.252)	0.387
CDH2	0.159	1.172	(0.947-1.451)	0.144
CDH4	-0.197	0.821	(0.312-2.160)	0.69
CDH11	-0.032	0.969	(0.761-1.234)	0.798
CDH12	-0.419	0.658	(0.297-1.454)	0.3
CDH13	0.331	1.392	(0.973-1.992)	0.071

The coefficient represents the regression coefficient. HR represents the hazard ratio, and 95% confidential interval are showed in (95% CI).

Supplementary Table 1C. Univariate Cox regression analysis of CDH1/2/4/11/12/13 and age, gender, tumor stage with overall survival in molecular subtype-HER2 of breast cancer (BRCA) patients.

Univariate	BRCA-HER2			
	Overall survival			
	Coefficient	HR	(95% CI)	*p* value
Age	0.086	1.09	(1.018-1.168)	0.014*
stage2	-1.178	0.308	(0.022-4.285)	0.381
stage3	0.474	1.606	(0.112-22.999)	0.727
stage4	3.033	20.763	(1.181-365.073)	0.038*
CDH1	-0.404	0.668	(0.429-1.040)	0.074
CDH2	0.459	1.583	(0.653-3.835)	0.309
CDH4	-29.735	0	(0-0.072)	0.032*
CDH11	0.4	1.492	(0.351-6.339)	0.587
CDH12	3.279	26.541	(1.308-538.706)	0.033*
CDH13	-0.442	0.643	(0.043-9.605)	0.749

The coefficient represents the regression coefficient. HR represents the hazard ratio, and 95% confidential interval are showed in (95% CI).

Supplementary Table 1D. Univariate Cox regression analysis of CDH1/2/4/11/12/13 and age, gender, tumor stage with overall survival in molecular subtype-Basal of breast cancer (BRCA) patients.

Univariate	BRCA-basal			
	Overall survival			
	Coefficient	HR	(95% CI)	*p* value
Age	0.023	1.023	(0.986-1.063)	0.226
stage2	18.162	77244950	(2.8e+07-2.1e+08)	0.000***
stage3	19.179	2.13e+08	(7.5e+07-6.1e+08)	0.000***
stage4	22.124	4.06e+09	(3.7e+08-4.5e+10)	0.000***
CDH1	-0.048	0.953	(0.651-1.395)	0.805
CDH2	-0.26	0.771	(0.553-1.074)	0.124
CDH4	-0.089	0.915	(0.617-1.358)	0.659
CDH11	0.427	1.533	(1.064-2.209)	0.022*
CDH12	0.899	2.457	(1.224-4.935)	0.012*
CDH13	0.156	1.169	(0.684-2.000)	0.567

The coefficient represents the regression coefficient. HR represents the hazard ratio, and 95% confidential interval are showed in (95% CI).

Supplementary Table 2. Pathway analysis of genes co-expressed cadherin 1 (*CDH1*) from the MetaCore database (with p-value< 0.05 set as the cutoff value) (Supplementary Table 2 and Figure 8).

#	Maps	p-Value	Network objects from active data
1	Immune response_B cell antigen receptor (BCR) pathway	1.298E-16	STIM1, Calcineurin A (catalytic), GRB2, alpha-4/beta-1 integrin, ICAM1, c-Rel (NF-kB subunit), H-Ras, NF-kB p50/c-Rel, ERK1/2, EGR1, c-Raf-1, BCAP, Fibronectin, CD19, VAV-1, Rac1, IP3 receptor, BAD, Syk, Cyclin D2, Shc, PI3K reg class IA (p85), GSK3 beta, Rb protein, CD79A, CDK4, FKHR, MEK6(MAP2K6), VAV-2, MEK2(MAP2K2), GSK3 alpha/beta, Bcl-XL, MEK1/2, CDC42, WASP, Actin cytoskeletal, NF-kB, AKT(PKB), NF-kB p50/p65, PDK (PDPK1), TAK1(MAP3K7), NF-AT2(NFATC1), MEKK4(MAP3K4), MEK1(MAP2K1), VCAM1, RelA (p65 NF-kB subunit), LRRK1, B-Raf, NF-kB1 (p50), K-RAS, ETS1, Lyn, PIP5KIII, CIN85, CD79 complex, PP2A catalytic, PIP5KI, PKC-beta2, NFKBIA, PI3K cat class IA (p110-delta), CARD11, CDK6, HPK1(MAP4K1), PKC-beta, p70 S6 kinase1, Calmodulin, CalDAG-GEFIII, CKLFSF7, MEKK1(MAP3K1), ATF-2, MALT1, IKK-beta, PLC-gamma, CD79B, Bcl-10, Calcineurin B (regulatory), PLC-gamma 2, p38 MAPK, Elk-1
2	Oxidative stress_ROS-induced cellular signaling	6.173E-16	Casein kinase II, alpha chains, p38alpha (MAPK14), Tuberin, SREBP1 (nuclear), SCD, ERK1/2, EGR1, VEGF-A, PKA-reg (cAMP-dependent), TXNIP (VDUP1), p21, Bak, Cytochrome c, FASN, E2I, Syk, Bax, GSK3 beta, FTH1, IRP2, GRP75, MDM2, IL-6, NF-kB, AKT(PKB), Catalase, NF-kB p50/p65, Cyclin B1, c-Src, TNF-alpha, NOTCH1 (NICD), Thioredoxin, Chk2, ACACA, RelA (p65 NF-kB subunit), KEAP1, IRP1, HIF1A, SRX1, AMPK alpha subunit, Pin1, NIK(MAP3K14), Isoform p66 Shc, Glutaredoxin 1, NFKBIA, HSPA1A, GSTP1, COX-2 (PTGS2), ELAVL1 (HuR), HSF1, PKC-beta, NOTCH3 (3ICD), PRKD1, p70 S6 kinase1, GPX1, PKC, LKB1, PTEN, MEKK1(MAP3K1), HES1, HSP27, IKK-beta, DLC1 (Dynein LC8a), c-Abl, Cyclin D1, JNK(MAPK8-10), HIF-prolyl hydroxylase, HDAC1, SAE2, SP1, p53, NRF2, ADAM17, p38 MAPK, APEX, PAI1, NALP3
3	Development_Negative regulation of WNT/Beta-catenin signaling in the cytoplasm	6.950E-16	Casein kinase I delta, NOTCH1 receptor, CXXC4, VHL, DP1, NKD1, PP1-cat, RIPK4, Presenilin 1, Alpha-1 catenin, Laforin, FAF1, Casein kinase I epsilon, Beta-catenin, CYLD, CXXC5, DAB2, Nucleoredoxin, Dsh, Casein kinase I alpha, Rac1, YAP1 (YAp65), G-protein alpha-13, WWP1, STK4, SIAH1, GSK3 alpha/beta, LATS2, G-protein beta/gamma, WDR26, Skp2/TrCP/FBXW, TAZ, Axin, RACK1, HECTD1, LRP5/LRP6, Prickle-1, c-Cbl, STK3, Tcf(Lef), E2F1, Amer1, PP2A catalytic, SENP2, RNF185, A20, Cul1/Rbx1 E3 ligase, YAP1/TAZ, CDK6, ELAVL1 (HuR), PEG3, WNT5A, Malin, PKC-alpha, Porf-2, WNT, Beclin 1, Cyclin D1, p53, NKD2, DACT3, Frizzled, DACT1
4	Immune response_IFN-alpha/beta signaling via PI3K and NF-kB pathways	1.750E-15	Tuberin, AKT1, Cyclin D3, ISG15, IRS-2, Tyk2, RPS6, DHFR, NMI, eIF4E, ERK1/2, c-Raf-1, CDC25A, IFN-alpha/beta receptor, p21, IFNAR1, PKC-epsilon, CDK1 (p34), I-kB, p70 S6 kinases, PI3K reg class IA (p85), GSK3 beta, NF-kB2 (p100), Rb protein, CDK4, EMSY, IFN-alpha, p19, ISG54, CAK complex, CREB1, p130, MEK1/2, RSAD2, b-Myb, NF-kB, AKT(PKB), PDK (PDPK1), p107, PU.1, pRB/E2F4, IFI17, PI3K cat class IA, RelA (p65 NF-kB subunit), NF-kB2 (p52), PKC-delta, TRAF2, p130/E2F4, E2F1, p107/E2F4, IFNAR2, eIF4B, p90RSK1, eIF4G1/3, NIK(MAP3K14), MNK1, CDK2, PCNA, 4E-BP1, GBP1, IFIT1, E2F4, PKC-alpha, Cyclin E, eIF4A, FOXO3A, IRF7, MNK2(GPRK7), Cyclin A
5	Immune response_IL-4-induced regulators of cell growth, survival, differentiation and metabolism	2.174E-14	ATP6V1B2, RARbeta, DHA2, MCM5, IL-4R type I, CCR2, PPAR-alpha, SOCS1, CDC25A, p21, Cytochrome c, STAT1, Loricrin, CPT-1A, Filaggrin, EGR2 (Krox20), Bax, CDK4, FKHR, SOCS3, Bcl-XL, ACADM, FasL(TNFSF6), Osteoprotegerin, AKT(PKB), PPAR-gamma, GATA-3, A-FABP, MMP-13, PLEKHF1, ANGPTL4, MCM4, IL-4R type II, Cyclin D, CYP2E1, IL4RA, Bcl-6, SK4/IK1, Cathepsin V, CDK2, CDK6, AP-1, Cyclin E, HSD3B1, FOXO3A, PERC, STAT5, Cyclin A, STAT6, LPL
6	Neurogenesis_NGF/ TrkA MAPK-mediated signaling	1.343E-13	SPHK1, NEFL, TrkA, ERK5 (MAPK7), CDK5, GRB2, APS, Fra-1, H-Ras, ERK1/2, MEF2C, CrkL, EGR1, PP2A regulatory, SGK1, PKA-reg (cAMP-dependent), CRK, c-Raf-1, FRS2, DNAJA3 (TID1), p21, VGF, PKC-epsilon, Ephrin-A receptor 2, IP3 receptor, TY3H, Shc, MAPKAPK2, PLC-gamma 1, MEK6(MAP2K6), MAP2K5 (MEK5), KIDINS220, CREB1, MEK1/2, PLAUR (uPAR), M-Ras, RGS2, p107, Stromelysin-1, c-Src, KCTD11, GAB2, SHPS-1, MATK, RIN, B-Raf, NF-kB1 (p50), JunB, K-RAS, PKC-delta, PP2A catalytic, C3G, SH2B, p90Rsk, p130CAS, NGF, AP-1, Calmodulin, MAGI-2, PKC-lambda/iota, SHP-2, Cyclin D1, SORBS1, MMP-10, SP1, PVR, JMJD3, RIT, p38 MAPK, Elk-1, PKA-cat (cAMP-dependent), FosB
7	Development_Differentiation of white adipocytes	4.070E-13	p38alpha (MAPK14), SREBP1 (nuclear), GRB2, BMP4, H-Ras, ERK1/2, FOXC2, c-Raf-1, SMAD4, PRKAR1A, SREBP1 precursor, Shc, HIVEP2, Rb protein, Resistin, FTase-alpha, MEK2(MAP2K2), CREB1, MEK1/2, PPARGC1 (PGC1-alpha), INSIG1, p107, PPAR-gamma, TAK1(MAP3K7), MEK1(MAP2K1), A-FABP, CIDEC, p90RSK1, LIPS, RIP140, PSAT, Factor D, FTase, TAB1, Angiotensinogen, LXR-alpha, BMPR1A, SMAD1, Leptin, BMP2, LPL, Perilipin, C/EBPalpha, C/EBPdelta
8	Role of activation of WNT signaling in the progression of lung cancer	1.531E-12	DVL-3, RUNX3, WNT3A, FZD6, Oct-3/4, NKD1, SFRP2, Krm1, Lef-1, DVL-1, TCF7L2 (TCF4), DKK1, FZD8, Beta-catenin, Matrilysin (MMP-7), VEGF-A, p21, Dsh, Rac1, ING4, GSK3 beta, FZD2, Survivin, Axin2, EZH2, SKP2, FZD9, FZD7, DVL-2, Axin, CD147, RUVBL1, WNT4, ROR2, hASH1, WNT9A, Tcf(Lef), Porcn, WNT3, FZD3, ARD1, WNT1, SFRP4, WNT2B, WNT5A, LKB1, MEKK1(MAP3K1), WNT, WNT10B, LRP6, SUZ12, Cyclin D1, SFRP1, p38 MAPK, Frizzled, NOTCH3

9	Development_Negative regulation of WNT/Beta-catenin signaling in the nucleus	2.248E-12	ZNF703, Casein kinase I delta, AKT1, Calcineurin A (catalytic), RUNX3, Oct-3/4, VHL, Lef-1, PGAM5, Alpha-1 catenin, TCF7L2 (TCF4), 14-3-3, Jade-1, Casein kinase I epsilon, Beta-catenin, VEGF-A, BCL9/B9L, PC1-CTT, TLE, Dsh, Menin, GSK3 beta, Nephrocystin-4, HDAC2, HIC1, LATS2, RANBP3, CtBP, PJA2, HIC5, PPAR-gamma, Axin, TAK1(MAP3K7), SOX9, TCF7 (TCF1), KDM2, LRP5/LRP6, TRRAP, WWOX, NARF, c-Cbl, PKC-delta, Tcf(Lef), E2F1, SOX17, SENP2, Cul1/Rbx1 E3 ligase, NLK, RNF43, WNT5A, SOX2, WNT, FOXO3A, eNOS, CHIBBY, HDAC1, Plakoglobin, RUVBL2, CDX1, Frizzled, Histone H1, DACT1
10	Stellate cells activation and liver fibrosis	2.435E-12	COL1A1, GRO-2, Biglycan, TNF-R2, GRB2, ICAM1, WNT3A, CCL2, H-Ras, Beta-catenin, c-Raf-1, TRADD, SMAD4, PDGF-R-alpha, DAB2, Dsh, Smoothened, I-kB, Shc, PI3K reg class IA (p85), GSK3 beta, RIPK1, MEK2(MAP2K2), MMP-2, COL1A2, ERK2 (MAPK1), AKT(PKB), PDGF receptor, NF-kB p50/p65, TNF-R1, ERK1 (MAPK3), TNF-alpha, MEK1(MAP2K1), PI3K cat class IA, PTCH1, TRAF2, Tcf(Lef), TGF-beta receptor type II, IL1RAP, KLF6, NIK(MAP3K14), TIMP1, TRAF1, ACTA2, Cyclin D1, IRAK1/2, SMAD3, SP1, Elk-1, Frizzled, TLR2, PDGF-R-beta
11	Development_Positive regulation of WNT/Beta-catenin signaling in the cytoplasm	3.380E-12	Casein kinase II, alpha chains, Bcl-9, EGF, BIG1, GRB2, IRS-2, NKD1, UBE2B, PP1-cat, RIPK4, Alpha-1 catenin, 14-3-3, Beta-catenin, TGIF, SMAD4, Dsh, Rac1, YAP1 (YAp65), CDK1 (p34), PPP2R2A, SIAH1, USP25, GSK3 alpha/beta, ERK2 (MAPK1), AKT(PKB), Axin, GSKIP, HECTD1, ITGB1, LRP5/LRP6, SIAH2, Tcf(Lef), 14-3-3 zeta/delta, TGT, HSP105, PKA-reg type II (cAMP-dependent), PP2A catalytic, RNF220, COX-2 (PTGS2), Trabid, BIG2, Miz-1, USP9X, WNT, USP7, Jouberin, PP2C alpha, JNK(MAPK8-10), SMAD3, NKD2, MITF, PKA-cat (cAMP-dependent), Frizzled, SET7, DACT1
12	Apoptosis and survival_Regulation of apoptosis by mitochondrial proteins	4.849E-12	p38alpha (MAPK14), Maspin, PLSCR3, Calcineurin A (catalytic), MPTP complex, Apaf-1, Cathepsin H, Granzyme B, ROCK1, ERK1/2, MUL1, RAD9A, PP2C, VDAC 2, Bik, Bak, Cytochrome c, Caspase-8, OPA1, Endonuclease G, NIP3, BAD, GZMH, Bax, SOD1, RASSF1, PINK1, PP1-cat alpha, AMBRA1, Mitofusin 1, Caspase-2, Bcl-XL, MTCH2, VDAC 1, MIDUO, Calpain 1(mu), Mcl-1, Cathepsin D, Granzyme K, Cathepsin L, IFI27, Aif, GC1QBP, PKC-delta, 14-3-3 zeta/delta, MAP1, Pin1, PP2A catalytic, PARL, Caspase-9, Metaxin 1, BMF, JSAP1, RAD9, CDK2, ATF-2, SLC25A3, TIMM8A, tBid, Beclin 1, HtrA2, Cyclin A, JNK(MAPK8-10), JNK2(MAPK9), Caveolin-1, DNM1L (DRP1), Calcineurin B (regulatory), Smac/Diablo, p38 MAPK, Bid
13	T follicular helper cell dysfunction in SLE	4.885E-12	IL-7 receptor, MHC class II, ICOS-L, IL-7, Tyk2, IFN-gamma, BATF, CXCR5, CD28, OX40(TNFRSF4), KLF2, SLAM, IFN-alpha/beta receptor, STAT1, TRIM, I-kB, PI3K reg class IA (p85), FKHR, IFN-alpha, RIPK1, CD4, CD84, IL-6, CTLA-4, Fyn, NF-kB, AKT(PKB), ICOS, IL-2, CD40L(TNFSF5), BAFF(TNFSF13B), CD80, Osteopontin, PI3K cat class IA, DDX6, PI3K reg class IA (p85-alpha), MCPIP, TRAF2, BAFF-R, IL4RA, NIK(MAP3K14), Bcl-6, A20, JAK3, CARD11, RC3H2, 4E-BP1, Hedls, c-Maf, p70 S6 kinase1, MALT1, IKK-beta, CXCL13, SAP, PD-1, Bcl-10, TLR7, CD40(TNFRSF5), IL-21 receptor, STAT6, CD86, BLIMP1 (PRDI-BF1)
14	Signal transduction_Angiotensin II/ AGTR1 signaling via p38, ERK and PI3K	2.049E-11	RECK, ERK5 (MAPK7), GRB2, CCL2, H-Ras, eIF4E, ERK1/2, MEF2C, EGR1, MSK1/2 (RPS6KA5/4), c-Raf-1, HDAC4, p21, Angiotensin II, p47-phox, Syk, Shc, p70 S6 kinases, FKHR, cPLA2, MEK6(MAP2K6), MAP2K5 (MEK5), MMP-2, CREB1, IL-6, MEK1/2, G-protein beta/gamma, PPARGC1 (PGC1-alpha), Fyn, AKT(PKB), Catalase, PDK (PDPK1), c-Src, PDGF-D, MEKK4(MAP3K4), Pyk2(FAK2), JAK2, Osteopontin, PI3K cat class IA, PI3K reg class IA (p85-alpha), PKC-delta, ETS1, EGFR, AGTR1, MNK1, PLD2, p90Rsk, CaMK II delta, COX-2 (PTGS2), ELAVL1 (HuR), 4E-BP1, p130CAS, Calmodulin, SOD3 (EC-SOD), MMP-14, G-protein alpha-q, Cyclin D1, ATOX1, SP1, ADAM17, p38 MAPK, Elk-1, PAI1, PKA-cat (cAMP-dependent), PDGF-R-beta, ATP7A
15	Transcription_Negative regulation of HIF1A function	3.026E-11	FHL3, Casein kinase I delta, Sirtuin3, MCM5, RUNX3, VHL, FBXW7, SART1, KLF2, VCP, SKP1, UBXD7, Ubiquitin, ING4, GSK3 beta, MCM7, LAMP2, OS-9, EAF2, MDM2, HSP40, MCM2, HSP90, Calpain 1(mu), HSP70, SAT2, EGLN1, RACK1, DEC2, HSC70, PRDX2, CITED2, HIF1A, ARD1, PSMA7, Sirtuin2, HSP90 beta, Cul1/Rbx1 E3 ligase, AML1 (RUNX1), FHL1 (SLIM1), PTEN, Sirtuin7, SSAT, Elongin C, HIF-prolyl hydroxylase, PRDX4, CHIP, RUVBL2, p53, CITED4

Supplementary Table 3. Pathway analysis of genes co-expressed cadherin 2 (*CDH2*) from the MetaCore database (with p-value< 0.05 set as the cutoff value) (Supplementary Table 3 and Supplementary Figure 4).

#	Maps	p-Value	Network objects from active data
1	Cell adhesion_ECM remodeling	2.504E-18	MMP-13, TIMP3, SERPINE2, MSN (moesin), MMP-1, IL-8, MMP-14, MMP-2, Versican, MMP-12, IGF-1 receptor, Matrilysin (MMP-7), PLAUR (uPAR), alpha-5/beta-1 integrin, Actin cytoskeletal, MMP-10, Collagen IV, TIMP2, Fibronectin, Nidogen, Stromelysin-1, LAMA4, MMP-9, PAI1, PLAU (UPA), Syndecan-2, Collagen I, Kallikrein 3 (PSA), Collagen III
2	Cytoskeleton remodeling_Regulation of actin cytoskeleton organization by the kinase effectors of Rho GTPases	2.188E-15	Talin, RhoA, WRCH-1, MRCKalpha, Rac1-related, Cdc42 subfamily, RhoJ, Vinculin, MSN (moesin), ERM proteins, F-Actin cytoskeleton, Spectrin, MRLC, ARPC1B, RhoA-related, Caldesmon, Actin cytoskeletal, Alpha-actinin, Filamin A, RhoC, LIMK1, RhoB, TC10, MyHC, LIMK, MLCK, MRCK
3	IL-1 beta- and Endothelin-1-induced fibroblast/ myofibroblast migration and extracellular matrix production in asthmatic airways	1.775E-12	COL1A1, COL4A1, EDNRB, TIMP3, MMP-1, ERK1/2, MMP-2, Versican, IL-1RI, COL1A2, CTGF, PDGF-R-alpha, Thrombospondin 1, Fibronectin, Stromelysin-1, PAI1, EDNRA, Collagen I, PDGF-R-beta, Collagen III
4	TGF-beta-induced fibroblast/ myofibroblast migration and extracellular matrix production in asthmatic airways	4.672E-12	COL1A1, Biglycan, TGF-beta 2, ITGB1, COL4A1, MMP-13, TIMP3, Tenascin-C, MMP-1, ERK1/2, MMP-2, COL1A2, COL5A1, Collagen IV, TIMP2, ITGA5, Fibronectin, Stromelysin-1, MMP-9, PAI1, Collagen I, MEK4(MAP2K4), Collagen III, Thrombospondin 2
5	Role of TGF-beta 1 in fibrosis development after myocardial infarction	6.420E-12	COL1A1, Biglycan, EDNRB, Tenascin-C, MMP-1, MMP-2, ACTA2, COL1A2, CTGF, Angiotensin II, TIMP2, Thrombospondin 1, Fibronectin, Prolyl endopeptidase, MMP-9, PAI1, EDNRA, Collagen I, Collagen III
6	Glucocorticoid-induced elevation of intraocular pressure as glaucoma risk factor	1.080E-11	RhoA, ITGB1, WNT5A, ROR2, COL4A1, MMP-1, Filamin B (TABP), Antileukoproteinase 1, SERPINA3 (ACT), WNT2, MMP-2, Actin cytoskeletal, Alpha-actinin, Filamin A, Srp40, Collagen IV, Thrombospondin 1, Fibronectin, Stromelysin-1, PKC-epsilon, alpha-V/beta-5 integrin, PAI1, LAMA1, MLCK
7	Transcription_HIF-1 targets	3.805E-10	G3P2, TfR1, TGF-beta 2, Adipophilin, Carbonic anhydrase XII, Lysyl oxidase, ROR-alpha, Adrenomedullin, HIF1A, P4HA2, REDD1, MMP-2, Galectin-1, PLAUR (uPAR), P4HA1, 5’-NTD, GLUT3, ENO1, Angiopoietin 2, CTGF, Thrombospondin 1, MMP-9, PAI1, Carbonic anhydrase IX, Stanniocalcin 2, LRP1, PKM2, IBP3
8	Protein folding and maturation_Posttranslational processing of neuroendocrine peptides	2.237E-09	PAM, Trypsin, AVP-NPII, OT-NPI, GRP(1-27), AVP-Gly, GRP(18-27), GRP(1-27)Gly, AVP extracellular region, GRP precursor, OT, PCSK5, OT-Gly, GRP(1-17), Neurophysin-II, OT-Gly-Lys-Arg, NPI, ProGRP, AVP-Gly-Lys-Arg
9	Development_Positive regulation of WNT/Beta-catenin signaling at the receptor level	7.111E-09	Biglycan, RECK, ROR2, FZD2, WNT7B, LYPD6, Tenascin-C, Glypican-3, Tcf(Lef), SFRP2, WNT, ERK1/2, WLS, TMEM59, RAP-2A, Filamin A, MKK7 (MAP2K7), GPR124, Stromelysin-1, WNT5B, Frizzled
10	Cell adhesion_Tight junctions	1.170E-08	RhoA, Rich1, JAM3, Cingulin, Tubulin alpha, F-Actin, ARP3, INADL, MRLC, ACTR3, CGNL1, ZO-3, Actin cytoskeletal, PARD6, Claudin-1, Actin, Tubulin (in microtubules)
11	Beta-catenin-dependent transcription regulation in colorectal cancer	2.542E-08	MTS1 (S100A4), LAMC2, LAMC2 (80kDa), Calcyclin, Tenascin-C, IL-8, TCF7L2 (TCF4), Fascin, MMP-14, Lamin A/C, Beta-catenin, PLAUR (uPAR), LAMC2 (100kDa), Claudin-1, PLAU (UPA)

12	Cytoskeleton remodeling_Regulation of actin cytoskeleton nucleation and polymerization by Rho GTPases	2.568E-08	RhoA, RhoD, DRF, RhoF (Rif), Profilin, Rac1-related, Cdc42 subfamily, BAIAP2, FMNL3, FMNL2, F-Actin cytoskeleton, RhoA-related, Actin cytoskeletal, RhoC, RhoB, TC10, FNBP1
13	Cell adhesion_Integrin-mediated cell adhesion and migration	3.065E-08	Talin, RhoA, p130CAS, ITGB1, p190-RhoGEF, VCAM1, PKC, Vinculin, PINCH, Zyxin, F-Actin cytoskeleton, alpha-5/beta-1 integrin, Actin cytoskeletal, Alpha-parvin, Alpha-actinin, Collagen IV, Fibronectin, PARD6, Collagen I, Collagen III
14	Development_Negative regulation of WNT/Beta-catenin signaling in the cytoplasm	3.961E-08	WWP1, Prickle-1, WNT5A, STK4, SIAH1, PKC-alpha, Tcf(Lef), WNT, Amer1, beta-TrCP, Alpha-1 catenin, Beclin 1, NEDD4L, LATS2, Beta-catenin, Skp2/TrCP/FBXW, Nucleoredoxin, SENP2, NKD2, DACT3, Frizzled, DACT1, CDK6
15	FAK1 signaling in melanoma	4.004E-08	Talin, RhoA, p130CAS, ITGB1, ARCGAP22, PKC-alpha, ERK1/2, MMP-14, MMP-2, alpha-5/beta-1 integrin, Actin cytoskeletal, TIMP2, RhoC, ITGA5, Fibronectin, PLAU (UPA)

Supplementary Table 4. Pathway analysis of genes co-expressed cadherin 4 (*CDH4*) from the MetaCore database (with p-value< 0.05 set as the cutoff value) (Supplementary Table 4 and SupplementaryFigure 5).

#	Maps	p-Value	Network objects from active data
1	Protein folding and maturation_POMC processing	2.428E-13	PAM, proACTH, POMC, N-POMC, beta-Endorphin extracellular region, gamma-LPH, ACTH 1-17, Joining peptide (JP), DA-alphaMSH, gamma-MSH, beta-LPH, gamma2-MSH, beta-MSH, N-POC, gamma3-MSH, ACTH, alpha-MSH, CLIP
2	Beta-catenin-dependent transcription regulation in colorectal cancer	1.638E-11	COX-2 (PTGS2), ID2, MTS1 (S100A4), LAMC2, LAMC2 (80kDa), Calcyclin, Tenascin-C, IL-8, CDX2, L1CAM, Fascin, MMP-14, PLAUR (uPAR), LAMC2 (100kDa), SOX9, PLAU (UPA), MDR1, YAP1 (YAp65)
3	Cell adhesion_ECM remodeling	2.422E-11	MMP-13, SERPINE2, Caveolin-2, MSN (moesin), MMP-1, IL-8, EGFR, MMP-14, MMP-2, MMP-12, HB-EGF, Matrilysin (MMP-7), PLAUR (uPAR), Actin cytoskeletal, Collagen IV, TIMP2, Kallikrein 1, Stromelysin-1, LAMA4, PLAU (UPA), Syndecan-2, Kallikrein 3 (PSA)
4	Transcription_HIF-1 targets	2.755E-08	ID2, Adipophilin, Lysyl oxidase, Transferrin, ROR-alpha, Endothelin-1, Adrenomedullin, HIF1A, P4HA2, LOXL4, MMP-2, Galectin-1, PLAUR (uPAR), 5’-NTD, GLUT3, ENO1, CTGF, p21, IBP1, MGF, HGF receptor (Met), PGK1, SDF-1, MDR1, IBP3
5	Development_Regulation of epithelial-to-mesenchymal transition (EMT)	2.916E-08	N-cadherin, Endothelin-1, E2A, WNT, TGF-beta receptor type II, SLUG, EGFR, SIP1 (ZFHX1B), MMP-2, PDGF-A, IL-1RI, Caldesmon, ACTA2, Tropomyosin-1, PDGF-R-alpha, TWIST1, HGF receptor (Met), Vimentin, Frizzled, TGF-beta receptor type I
6	Role of stellate cells in progression of pancreatic cancer	5.083E-08	RECK, MMP-13, CCL2, IL-8, TGF-beta receptor type II, EGFR, ERK1/2, MMP-2, PDGF-A, HB-EGF, IL-6, Galectin-1, ACTA2, CTGF, PDGF-R-alpha, PDGF receptor, OSF-2, Stromelysin-1, TGF-beta receptor type I
7	Development_YAP/TAZ-mediated co-regulation of transcription	8.820E-08	ID2, TEF-3, Endothelin-1, FKHR, SOD2, HIF1A, CDX2, SLUG, SIP1 (ZFHX1B), Cyr61, ID3, HBP17, TAZ, CTGF, TWIST1, SOX9, YAP1 (YAp65), CDK6
8	Role of Tissue factor-induced Thrombin signaling in cancerogenesis	2.122E-07	MLCP (cat), MMP-13, PKC-alpha, IL-8, EGFR, G-protein alpha-i family, MRLC, ERK1/2, MMP-2, Angiopoietin 1, Actin cytoskeletal, Coagulation factor X, ERK2 (MAPK1), G-protein alpha-q/11, Tissue factor, PLC-beta3, ERK1 (MAPK3), MLCK, IP3 receptor
9	Expression targets of Tissue factor signaling in cancer	1.791E-06	ITGB1, VEGF-C, GFPT2, IL-8, Cyr61, PLAUR (uPAR), Coagulation factor X, CTGF, Tissue factor, PLAU (UPA)
10	Role of TGF-beta 1 in fibrosis development after myocardial infarction	2.492E-06	TMSB4X, EDNRB, Endothelin-1, Tenascin-C, MMP-1, TGF-beta receptor type II, MMP-2, ACTA2, Ac-SDKP, CTGF, TIMP2, Thymosin beta-4, TGF-beta receptor type I
11	MAPK-mediated proliferation of normal and asthmatic smooth muscle cells	2.689E-06	Amphiregulin, EDNRB, PLC-beta, Endothelin-1, PKC-alpha, EGFR, G-protein alpha-i family, ERK1/2, PDGF-A, HB-EGF, PDGF-C, PDGF-R-alpha, PDGF receptor, p90Rsk, IP3 receptor, TGF-beta receptor type I
12	G protein-coupled receptors signaling in lung cancer	2.940E-06	PGE2R4, Amphiregulin, EDNRB, Endothelin-1, IL-8, EGFR, G-protein alpha-i family, NTSR1, ERK1/2, MMP-2, PGE2R1, HB-EGF, Galpha(i)-specific peptide GPCRs, TGF-alpha, Galanin, Galpha(q)-specific peptide GPCRs, G-protein alpha-q/11, HB-EGF(mature), SDF-1

13	Cell adhesion_Desmosomes	3.919E-06	Keratin 17, Desmocollin 3, Keratin 14, Keratin 5, Keratin 1, Plakophilin 1, Desmoglein 3, Vimentin, DSC2
14	Bone metastases in Prostate Cancer	4.695E-06	Endothelin-1, CCL2, WNT, G-protein alpha-i family, DKK1, PTHR1, PTHrP, SDF-1, Frizzled, IBP3, Kallikrein 3 (PSA)
15	Signal transduction_PDGF signaling via MAPK cascades	5.014E-06	COX-2 (PTGS2), SPHK1, MMP-13, ERK1/2, MMP-2, PDGF-A, PDGF-C, IL-6, EGR1, PDGF-R-alpha, ERK2 (MAPK1), p21, PDGF receptor, Tissue factor, Stromelysin-1, p90Rsk, Phox1 (PRRX1)

Supplementary Table 5. Pathway analysis of genes co-expressed cadherin 7 (*CDH7*) from the MetaCore database (with p-value< 0.05 set as the cutoff value) (Supplementary Table 5 and Supplementary Figure 6).

#	Maps	p-Value	Network objects from active data
1	Cell cycle_Chromosome condensation in prometaphase	2.632E-18	Condensin, TOP1, Cyclin B, Histone H3, CAP-G, CAP-G/G2, CAP-H/H2, CAP-C, Cyclin A, CNAP1, CAP-D2/D3, Aurora-B, TOP2, BRRN1, Aurora-A, CAP-E, CDK1 (p34)
2	Cell cycle_The metaphase checkpoint	2.852E-16	HZwint-1, ZW10, Survivin, Nek2A, CENP-A, CDC20, MAD2a, CENP-H, SPBC25, BUB3, Aurora-B, HEC, CENP-E, BUB1, Aurora-A, PLK1, CDCA1, CENP-F, Zwilch, AF15q14
3	Cell cycle_Role of APC in cell cycle regulation	2.885E-16	Cyclin B, ORC1L, CKS1, Nek2A, CDC20, MAD2a, BUB3, Tome-1, Cyclin A, Aurora-B, CDC25A, BUB1, Geminin, Emi1, Aurora-A, PLK1, Securin, CDK1 (p34), CDK2
4	DNA damage_ATM/ATR regulation of G2/M checkpoint: cytoplasmic signaling	9.173E-15	p38alpha (MAPK14), Chk2, Chk1, Brca1, Histone H3, CDC25C, UBE2C, PP1-cat, Nucleolysin TIAR, 14-3-3, B56G, JAB1, PP2A regulatory, Aurora-B, JNK2(MAPK9), CDC25A, Cyclin B1, Aurora-A, PLK1, p38 MAPK, DCK, CDK1 (p34)
5	Cell cycle_Spindle assembly and chromosome separation	1.398E-14	Tubulin alpha, Cyclin B, Separase, ZW10, Nek2A, CDC20, MAD2a, Importin (karyopherin)-alpha, KNSL1, Aurora-B, HEC, TPX2, CSE1L, Aurora-A, Securin, Ran, CDK1 (p34), Tubulin (in microtubules)
6	DNA damage_Intra S-phase checkpoint	2.323E-13	PCNA, Chk2, MCM3, Chk1, MCM7, Brca1, SMC3, CDC7, Histone H3, MCM5, FANCD2, DTL (hCdt2), MCM4, FANCI (KIAA1794), PP1-cat, MCM10, PP1-cat alpha, Histone H2AX, Cyclin A, CDC25A, MCM2, Claspin, p38 MAPK, CDK2, CDC45L
7	DNA damage_ATM/ATR regulation of G2/M checkpoint: nuclear signaling	1.213E-11	HSF1, Chk2, Chk1, Brca1, Wee1, Cyclin B, Cyclin B2, CDC25C, PALB2, MDM2, Histone H2AX, Cyclin A, RBBP8 (CtIP), Cyclin B1, Claspin, PLK1, CDK1 (p34), CDK2
8	Cell cycle_Role of Nek in cell cycle regulation	4.024E-11	Tubulin beta, Tubulin gamma, PI3K cat class IA, Tubulin alpha, Histone H3, Nek2A, MAD2a, HEC, Cyclin B1, TPX2, PDK (PDPK1), Aurora-A, Ran, CDK1 (p34), Tubulin (in microtubules)
9	Cell cycle_Start of DNA replication in early S phase	5.728E-10	MCM3, CDC7, ORC1L, MCM5, RPA3, DP1, MCM4, MCM10, ORC6L, MCM4/6/7 complex, MCM2, Geminin, CDK2, CDC45L
10	Abnormalities in cell cycle in SCLC	1.651E-09	PCNA, E2F2, Histone H3, CKS1, CDK4, BMI-1, MDM2, Cyclin A, Aurora-B, Cyclin B1, CDK1 (p34), Cyclin E2, CDK2
11	Ubiquinone metabolism	2.963E-09	NDUFA8, NDUFB3, NDUFS8, NDUFS3, DAP13, NDUFS5, NDUFAB1, NDUFB1, NDUFB5, NDUFC1, NDUFV3, NDUFA7, NDUFA9, NDUFS4, COQ3, NDUFB2, coenzyme Q2 homolog, prenyltransferase (yeast), NDUFA4, NDUFB6, NDUFB8
12	Cell cycle_Cell cycle (generic schema)	3.800E-09	Cyclin B, E2F2, CDC25C, CDK4, E2F5, DP1, Cyclin A, CDC25A, p107, CDK1 (p34), CDK2
13	ATP/ITP metabolism	4.216E-09	AK2, CSL4, POLR1A, RRP4, RRM2B, PPA5, RRM2, RRP46, ADSS, ACYP2, Ribonucleotide reductase, POLR2I, POLR2B, RRP40, POLR2G, RPA16, RPA39, POLR2D, POLR2J, Adenosine kinase, PM/SCL-75, HPRT, POLR3K, Small RR subunit, RRM1, 5’-NT1B
14	Cell cycle_Initiation of mitosis	4.695E-09	Wee1, Cyclin B2, Histone H3, CDC25C, FOXM1, Kinase MYT1, Lamin B, KNSL1, Cyclin B1, PLK1, CDK1 (p34), CDK7
15	Cell cycle_Sister chromatid cohesion	1.312E-08	PCNA, TOP1, SMC3, Cyclin B, RFC3, Separase, Histone H3, DERPC, DCC1, Securin, CDK1 (p34)

Supplementary Table 6. Pathway analysis of genes co-expressed cadherin 11 (*CDH11*) from the MetaCore database (with p-value< 0.05 set as the cutoff value) (Supplementary Table 6 and Supplementary Figure 7).

#	Maps	p-Value	Network objects from active data
1	Cell adhesion_ECM remodeling	2.784E-19	MMP-13, TIMP3, SERPINE2, Caveolin-2, PLAT (TPA), MSN (moesin), MMP-1, IL-8, IGF-2, MMP-14, MMP-2, Versican, MMP-12, Matrilysin (MMP-7), PLAUR (uPAR), alpha-5/beta-1 integrin, MMP-10, TIMP2, IGF-1, Fibronectin, Kallikrein 1, Nidogen, Stromelysin-1, LAMA4, PAI1, PLAU (UPA), Syndecan-2, Collagen I, Kallikrein 3 (PSA), Collagen III
2	IL-1 beta- and Endothelin-1-induced fibroblast/ myofibroblast migration and extracellular matrix production in asthmatic airways	2.862E-18	COL1A1, IL-1 beta, AP-1, EDNRB, TIMP3, Decorin, CCL2, MMP-1, HAS2, c-Jun, MMP-2, Versican, IL-1RI, c-Jun/c-Fos, COL1A2, CTGF, PDGF-R-alpha, Thrombospondin 1, Fibronectin, Stromelysin-1, PAI1, EDNRA, Collagen I, PDGF-R-beta, Collagen III
3	Development_Regulation of epithelial-to-mesenchymal transition (EMT)	6.645E-18	IL-1 beta, HEY1, VE-cadherin, N-cadherin, TGF-beta 2, TGF-beta 3, WNT, TGF-beta receptor type II, SLUG, ZO-1, Lef-1, c-Jun, Sno-N, SIP1 (ZFHX1B), MMP-2, IL-1RI, Caldesmon, ACTA2, Tropomyosin-1, PDGF-R-alpha, TWIST1, Fibronectin, Vimentin, TCF8, Claudin-1, PAI1, PDGF-D, Frizzled, EDNRA, FGFR1, PDGF-R-beta
4	Role of TGF-beta 1 in fibrosis development after myocardial infarction	4.860E-14	COL1A1, Biglycan, TMSB4X, EDNRB, Tenascin-C, MMP-1, SMAD7, TGF-beta receptor type II, MMP-2, ACTA2, Ac-SDKP, COL1A2, CTGF, TIMP2, Thymosin beta-4, Thrombospondin 1, Fibronectin, PAI1, EDNRA, Collagen I, Collagen III
5	TGF-beta-induced fibroblast/ myofibroblast migration and extracellular matrix production in asthmatic airways	8.853E-14	COL1A1, Biglycan, TGF-beta 2, ITGB1, AP-1, MMP-13, TIMP3, Decorin, TGF-beta 3, Tenascin-C, MMP-1, HAS2, TGF-beta receptor type II, c-Jun, MMP-2, c-Jun/c-Fos, COL1A2, COL5A1, TIMP2, ITGA5, Fibronectin, Stromelysin-1, PAI1, Collagen I, Collagen III, Thrombospondin 2
6	Development_TGF-beta-dependent induction of EMT via SMADs	1.227E-12	ID2, HEY1, N-cadherin, TGF-beta 2, TGF-beta 3, ETS1, TGF-beta receptor type II, SLUG, Lef-1, SIP1 (ZFHX1B), MMP-2, TGF-beta, TWIST1, Fibronectin, Vimentin, TCF8, Claudin-1, PAI1, ILK
7	Role of adhesion of SCLC cells in tumor progression	1.634E-11	RhoA, N-cadherin, ITGB1, VCAM1, alpha-4/beta-1 integrin, Tenascin-C, alpha-V/beta-1 integrin, MMP-14, MMP-2, CDC42, alpha-5/beta-1 integrin, PTHrP, CD9, Caveolin-1, Fibronectin, Stromelysin-1, SDF-1, CXCR4
8	TGF-beta 1-mediated induction of EMT in normal and asthmatic airway epithelium	2.237E-11	COL1A1, IL-1 beta, N-cadherin, AP-1, Tenascin-C, SMAD7, ETS1, TGF-beta receptor type II, SLUG, ZO-1, c-Jun, MMP-2, ACTA2, c-Jun/c-Fos, CTGF, TWIST1, DAB2, Fibronectin, Vimentin, PAI1
9	Development_Role of proteases in hematopoietic stem cell mobilization	2.666E-11	Cathepsin G, VCAM1, alpha-4/beta-1 integrin, MMP-14, MMP-2, alpha-5/beta-1 integrin, DPP4, MGF, Fibronectin, c-Kit, Cathepsin K, SDF-1, CXCR4
10	Role of stellate cells in progression of pancreatic cancer	5.084E-11	COL1A1, RECK, MMP-13, c-Fos, CCL2, IL-8, TGF-beta receptor type II, c-Jun, MMP-2, Galectin-1, ID1, ACTA2, alpha-5/beta-1 integrin, COL1A2, CTGF, PDGF-R-alpha, PDGF receptor, OSF-2, Fibronectin, Stromelysin-1, Collagen I, PDGF-R-beta, Collagen III
11	Signal transduction_WNT/Beta-catenin signaling in tissue homeostasis	7.895E-11	Cyclin D2, FGF18, Cyclin A2, FKHR, BACE1, Tcf(Lef), IL-8, WNT, SLUG, Lef-1, Pitx2, TCF7L2 (TCF4), Connexin 43, MMP-2, Versican, Beta-catenin, KLF4, PAI1, Frizzled

12	TGF-beta signaling via SMADs in breast cancer	9.851E-11	TGF-beta 2, MMP-13, MTS1 (S100A4), RUNX2, NOX4, JunB, TGF-beta 3, IL-8, ETS1, TGF-beta receptor type II, SLUG, Fascin, MMP-14, ITGB5, PTHrP, CTGF, TWIST1, FOXQ1 (HFH1), PAI1, GLI-2
13	Glucocorticoid-induced elevation of intraocular pressure as glaucoma risk factor	1.112E-10	RhoA, ITGB1, ROCK, WNT5A, ROR2, GCR Beta, GCR, PLAT (TPA), MMP-1, Elastin, Filamin B (TABP), WNT2, MMP-2, CDC42, GCR Alpha, Thrombospondin 1, Fibronectin, Stromelysin-1, alpha-V/beta-5 integrin, PAI1, LAMA1, MLCK, FGFR1
14	Production and activation of TGF-beta in airway smooth muscle cells	1.946E-10	RhoA, TGF-beta 2, ROCK, AP-1, c-Fos, TGF-beta 3, TGF-beta receptor type II, PAR2, G-protein alpha-i family, c-Jun, EGR1, c-Jun/c-Fos, TGF-beta, Beta-tryptase 2, Tryptase, PAI1, PLAU (UPA)
15	Development_TGF-beta-dependent induction of EMT via RhoA, PI3K and ILK	5.404E-10	RhoA, TGF-beta 2, TGF-beta 3, PINCH, TGF-beta receptor type II, SLUG, ZO-1, Lef-1, ROCK1, Beta-catenin, Caldesmon, ACTA2, Tropomyosin-1, HIC5, Fibronectin, Vimentin, Claudin-1, Actin, ILK

Supplementary Table 7. Pathway analysis of genes co-expressed cadherin 12 (*CDH12*) from the MetaCore database (with p-value< 0.05 set as the cutoff value) (Supplementary Table 7).

#	Maps	p-Value	Network objects from active data
1	Cytoskeleton remodeling_Regulation of actin cytoskeleton organization by the kinase effectors of Rho GTPases	1.414E-09	RhoA, WRCH-1, DMPK, PAK, Rac1-related, Cdc42 subfamily, RhoJ, ERM proteins, Spectrin, MRLC, SLC9A1, RhoA-related, Alpha adducin, RhoC, RhoB, TC10, MyHC, MRCK
2	Development_Negative regulation of WNT/Beta-catenin signaling in the cytoplasm	5.128E-07	ELAVL1 (HuR), KLHL12, WWP1, STK4, Porf-2, WNT, beta-TrCP, Itch, Alpha-1 catenin, NEDD4L, GSK3 alpha/beta, Skp2/TrCP/FBXW, TAZ, Cyclin D1, CXXC5, RNF185, Axin, YAP1/TAZ
3	LRRK2 in neurons in Parkinson's disease	1.038E-06	GSK3 beta, AP-2 alpha subunits, Beta-adaptin 2, PKC-zeta, MARKK, Tau (MAPT), RAB-5B, AP2A2, CHIP, PKA-cat (cAMP-dependent), VIL2 (ezrin)
4	Development_Positive regulation of WNT/Beta-catenin signaling in the cytoplasm	3.932E-06	PPP2R2A, TBL1X, Bcl-9, Trabid, USP25, WNT, USP7, Alpha-1 catenin, Jouberin, FAK1, GSK3 alpha/beta, IGF-1 receptor, SMAD3, Makorin-1, Axin, PKA-cat (cAMP-dependent)
5	Cytoskeleton remodeling_Regulation of actin cytoskeleton nucleation and polymerization by Rho GTPases	6.154E-06	RhoA, RhoD, RhoF (Rif), CYFIP2, Rac1-related, Cdc42 subfamily, BAIAP2, RhoA-related, RhoC, RhoB, TC10, FNBP1
6	Development_Positive regulation of STK3/4 (Hippo) pathway and negative regulation of YAP/TAZ function	7.507E-06	RhoA, STK4, AMPK beta subunit, Adenylate cyclase, beta-TrCP, CCDC85C, Itch, Alpha-1 catenin, EBP50, Skp2/TrCP/FBXW, TAZ, MARKK, Axin, PKA-cat (cAMP-dependent), Alpha-catenin
7	Development_Negative regulation of WNT/Beta-catenin signaling in the nucleus	7.966E-06	NF-AT5, TBL1X, GSK3 beta, Oct-3/4, WNT, Alpha-1 catenin, Jade-1, P15RS, CHIBBY, BCL9/B9L, GLI-3R, PC1-CTT, CHD8, TLE, HIC5, Axin, Histone H1
8	PI3K signaling in gastric cancer	1.560E-05	ELAVL1 (HuR), PI3K reg class IA (p85), GSK3 beta, PI3K reg class IA, PTEN, HSP27, FAK1, ErbB3, Cyclin D1, PRNP, G-protein alpha-q/11, MDR1
9	Signal transduction_mTORC2 downstream signaling	2.056E-05	RhoA, Tuberin, GSK3 beta, SREBP1 (nuclear), PKC, STK4, NEDD4L, GSK3 alpha/beta, PKC-zeta, IGF-1 receptor, Cyclin D1, Adenylate cyclase type IX, PKA-cat (cAMP-dependent), PREX1
10	Signal transduction_Cyclic AMP signaling	3.383E-05	PKC, cAMP-GEFI, PHK alpha, PHK gamma, GSK3 alpha/beta, Adenylate cyclase type VI, PKC-zeta, CREB1, Adenylate cyclase type IX, PKA-cat (cAMP-dependent)
11	Regulation of lipid metabolism_Regulation of lipid metabolism via LXR, NF-Y and SREBP	3.383E-05	SREBP1 precursor, Importin (karyopherin)-beta, ACLY, SREBP1 (nuclear), AMPK beta subunit, RARalpha, RXRA, CREB1, SP1, SREBP1 (Golgi membrane)
12	Signal transduction_IGF-1 receptor signaling pathway	7.566E-05	Androgen receptor, PI3K reg class IA (p85), GSK3 beta, SREBP1 (nuclear), Bcl-2, Cyclin D, FAK1, PKC-zeta, IGF-1 receptor, MNK2(GPRK7), MKK7 (MAP2K7), ACSA
13	WNT signaling in gastric cancer	1.292E-04	GSK3 beta, CD44, WNT, beta-TrCP, WNT3, Skp2/TrCP/FBXW, Cyclin D1, Axin, FBXW11
14	DNA damage_ATM/ATR regulation of G1/S checkpoint	1.306E-04	ELAVL1 (HuR), Chk2, SMG1, FBXW7, PP2A structural, beta-TrCP, PP2A regulatory, Cyclin D1, PER3, FBXW11
15	Main pathways of Schwann cells transformation in neurofibromatosis type 1	1.344E-04	Tuberin, PI3K reg class IA (p85), Amphiregulin, GSK3 beta, ErbB2, PTEN, Bcl-2, FAK1, ErbB3, Neurofibromin, IGF-1 receptor, BRD4, Cyclin D1, ErbB4

Supplementary Table 8. Pathway analysis of genes co-expressed cadherin 13 (*CDH13*) from the MetaCore database (with p-value< 0.05 set as the cutoff value) (Supplementary Table 8 and Supplementary Figure 8).

#	Maps	p-Value	Network objects from active data
1	Development_Regulation of epithelial-to-mesenchymal transition (EMT)	3.068E-16	IL-1 beta, HEY1, VE-cadherin, N-cadherin, TGF-beta 2, WNT, TGF-beta receptor type II, SLUG, ZO-1, SIP1 (ZFHX1B), MMP-2, PDGF-A, Caldesmon, ACTA2, Tropomyosin-1, PDGF-R-alpha, TWIST1, Fibronectin, Jagged1, Vimentin, TCF8, DLL4, Claudin-1, PAI1, PDGF-D, Frizzled, EDNRA, FGFR1, PDGF-R-beta
2	Role of stellate cells in progression of pancreatic cancer	3.846E-16	COL1A1, MEK1(MAP2K1), NGF, RECK, MMP-13, c-Fos, IL-8, TGF-beta receptor type II, alpha-V/beta-3 integrin, MEK2(MAP2K2), MMP-2, PDGF-A, HB-EGF, Galectin-1, ID1, ACTA2, alpha-5/beta-1 integrin, SOS, COL1A2, CTGF, PDGF-R-alpha, PDGF receptor, OSF-2, Fibronectin, NFKBIA, Collagen I, PDGF-R-beta, Collagen III
3	Cell adhesion_ECM remodeling	2.966E-15	MMP-13, TIMP3, SERPINE2, Caveolin-2, PLAT (TPA), IL-8, MMP-2, Versican, MMP-12, HB-EGF, PLAUR (uPAR), alpha-5/beta-1 integrin, Actin cytoskeletal, MMP-10, Collagen IV, TIMP2, Fibronectin, Kallikrein 1, Nidogen, LAMA4, PAI1, PLAU (UPA), Syndecan-2, Collagen I, Kallikrein 3 (PSA), Collagen III
4	IL-1 beta- and Endothelin-1-induced fibroblast/ myofibroblast migration and extracellular matrix production in asthmatic airways	9.808E-14	COL1A1, IL-1 beta, AP-1, COL4A1, EDNRB, TIMP3, Decorin, HAS2, MMP-2, Versican, COL1A2, NF-kB, CTGF, PDGF-R-alpha, Thrombospondin 1, Fibronectin, PAI1, EDNRA, Collagen I, PDGF-R-beta, Collagen III
5	TGF-beta-induced fibroblast/ myofibroblast migration and extracellular matrix production in asthmatic airways	2.572E-11	COL1A1, Biglycan, TGF-beta 2, ITGB1, AP-1, COL4A1, MMP-13, TIMP3, Decorin, Tenascin-C, HAS2, TGF-beta receptor type II, MMP-2, COL1A2, COL5A1, Collagen IV, TIMP2, ITGA5, Fibronectin, PAI1, Collagen I, Collagen III, Thrombospondin 2
6	Role of TGF-beta 1 in fibrosis development after myocardial infarction	5.509E-11	COL1A1, Biglycan, EDNRB, Tenascin-C, TGF-beta receptor type II, MMP-2, AGTR1, ACTA2, COL1A2, CTGF, Angiotensin II, TIMP2, Thrombospondin 1, Fibronectin, PAI1, EDNRA, Collagen I, Collagen III
7	MAPK-mediated proliferation of normal and asthmatic smooth muscle cells	3.070E-10	LPAR2, Rb protein, EDNRB, c-Fos, PLAT (TPA), G-protein alpha-I family, MKP-1, PDGF-A, HB-EGF, PDGF-C, MEK1/2, G-protein beta/gamma, SOS, PDGF-R-alpha, PDGF receptor, Elk-1, PAI1, p90Rsk, EDNRA, FGFR1, PDGF-R-beta
8	Stimulation of TGF-beta signaling in lung cancer	7.310E-10	IL-1 beta, N-cadherin, TGF-beta 2, I-kB, ITGB1, Vinculin, TGF-beta receptor type II, SLUG, MMP-2, ACTA2, EGR1, VEGF-A, TGF-beta, Tropomyosin-1, MMP-28, Fibronectin, Vimentin, PAI1, Tropomyosin-2
9	Cell adhesion_PLAU signaling	1.144E-09	STAT3, MEK1(MAP2K1), LAMC2, NF-kB2 (p52), alpha-V/beta-1 integrin, alpha-V/beta-3 integrin, G-protein alpha-I family, F-Actin cytoskeleton, MRLC, PLAUR (uPAR), alpha-5/beta-1 integrin, VEGFR-2, SOS, NF-kB, Caveolin-1, MYLK1, sUPAR, alpha-V/beta-5 integrin, PLAU (UPA), MLCK, STAT1, PDGF-R-beta
10	Glucocorticoid-induced elevation of intraocular pressure as glaucoma risk factor	2.691E-09	ITGB1, WNT5A, COL4A1, GCR Beta, GCR, PLAT (TPA), ITGB3, alpha-V/beta-3 integrin, WNT2, MMP-2, Actin cytoskeletal, GCR Alpha, LAMB3, Collagen IV, Thrombospondin 1, Fibronectin, alpha-V/beta-5 integrin, PAI1, LAMA1, MLCK, FGFR1
11	Stromal-epithelial interaction in Prostate Cancer	3.849E-09	Keratin 17, TGF-beta 2, Tenascin-C, TGF-beta receptor type II, MMP-2, Versican, PDGF-A, ACTA2, PDGF-R-alpha, HIC5, TIMP2, Fibronectin, Vimentin, PDGF-D, FGFR1, Collagen I, PDGF-R-beta

12	Development_TGF-beta-dependent induction of EMT via MAPK	3.859E-09	MEK1(MAP2K1), TGF-beta 2, ITGB1, MLK3(MAP3K11), AP-1, c-Fos, NOX4, ITGB3, alpha-V/beta-1 integrin, TGF-beta receptor type II, MEK2(MAP2K2), MMP-2, TGF-beta, SOS, Fibronectin, Vimentin, Claudin-1, PAI1
13	TGF-beta 1-mediated induction of EMT in normal and asthmatic airway epithelium	8.956E-09	COL1A1, IL-1 beta, N-cadherin, AP-1, Tenascin-C, ITGB3, TGF-beta receptor type II, SLUG, ZO-1, MMP-2, ACTA2, CTGF, TWIST1, Fibronectin, Jagged1, Vimentin, PAI1
14	Development_TGF-beta-dependent induction of EMT via SMADs	1.255E-08	HEY1, N-cadherin, TGF-beta 2, TGF-beta receptor type II, SLUG, SIP1 (ZFHX1B), MMP-2, TGF-beta, TWIST1, Fibronectin, Jagged1, Vimentin, TCF8, Claudin-1, PAI1
15	Signal transduction_Angiotensin II/AGTR1 signaling via Notch, Beta-catenin and NF-kB pathways	2.318E-08	COL1A1, HEY1, I-kB, PRKD1, NF-kB2 (p100), NF-kB2 (p52), IL-8, Connexin 43, MMP-2, AGTR1, Axin2, Beta-catenin, ACTA2, HEY2, Angiotensinogen, VEGF-A, NF-kB, CTGF, NIK(MAP3K14), Angiotensin II, Fibronectin, YAP1 (Yap65)

Supplementary Table 9. Pathway analysis of genes co-expressed cadherin 15 (*CDH15*) from the MetaCore database (with p-value< 0.05 set as the cutoff value) (Supplementary Table 9 and Supplementary Figure 9).

#	Maps	p-Value	Network objects from active data
1	Transcription_HIF-1 targets	6.658E-17	PDK1, NIX, P4HA2, PFKL, REDD1, SLC9A1, VEGF-A, Cyclin G2, 5'-NTD, GLUT3, ENO1, p21, MSH6, Carbonic anhydrase IX, PGK1, Stanniocalcin 2, NIP3, MDR1, AK3, IBP3, ID2, Adipophilin, LOXL4, MMP-2, PLAUR (uPAR), WT1, Alpha-1B adrenergic receptor, DEC1 (Stra13), ALDOC, c-Myc, Epo, SDF-1, CXCR4, LRP1, PKM2, G3P2, ARNT, Heme oxygenase 1, TGF-beta 2, Ceruloplasmin, Lysyl oxidase, ROR-alpha, Adrenomedullin, CITED2, HIF1A, HXK1, HIF-1, GPI, P4HA1, FGF2, Angiopoietin 2, IBP1, MGF, Thrombospondin 1, TfR1, GLUT1, Carbonic anhydrase XII, Transferrin, SOX2, CX3CR1, TGM2, Galectin-1, NOXA, CTGF, MCT4, LDHA, ABCG2, p53, HGF receptor (Met), Leptin, MMP-9, PAI1
2	Oxidative stress_ROS-induced cellular signaling	7.281E-17	Casein kinase II, alpha chains, Tuberin, SREBP1 (nuclear), SCD, ERK1/2, EGR1, VEGF-A, PKA-reg (cAMP-dependent), TXNIP (VDUP1), IKK (cat), p21, Bak, Cytochrome c, FASN, E2I, Carbonic anhydrase IX, Syk, IL-1 beta, Bax, GSK3 beta, FTL, IKK-alpha, FTH1, IRP2, GRP75, MDM2, IL-6, GADD45 alpha, NF-kB, AKT(PKB), Catalase, Cyclin B1, c-Src, PUMA, NOTCH1 (NICD), Thioredoxin, Heme oxygenase 1, PLK3 (CNK), Chk2, Adrenomedullin, HIF1A, IL-8, SRX1, AMPK alpha subunit, Pin1, NIK(MAP3K14), Glutaredoxin 1, p300, NFKBIA, HSPA1A, GSTP1, TfR1, ELAVL1 (HuR), HSF1, PKC-beta, PRKD1, Sirtuin1, GPX1, PKC, LKB1, PTEN, MEKK1(MAP3K1), HES1, IKK-beta, DLC1 (Dynein LC8a), NOXA, Cyclin D1, HIF-prolyl hydroxylase, HDAC1, SAE2, SP1, p53, NRF2, ADAM17, p38 MAPK, APEX, PAI1, mTOR, NALP3
3	Development_Negative regulation of WNT/Beta-catenin signaling in the cytoplasm	6.919E-14	Casein kinase I delta, NOTCH1 receptor, CXXC4, NKD1, PP1-cat, RIPK4, APC protein, Alpha-1 catenin, Casein kinase I epsilon, Beta-catenin, CYLD, CXXC5, Nucleoredoxin, Dsh, Casein kinase I alpha, WWP1, STK4, SIAH1, Itch, GSK3 alpha/beta, Ankyrin-G, G-protein beta/gamma, WDR26, Skp2/TrCP/FBXW, TAZ, Axin, KCTD1, RACK1, KLHL12, E-cadherin, HECTD1, Prickle-1, HIPK2, STK3, Tcf(Lef), HUWE1, Amer1, beta-TrCP, PP2A catalytic, PR72, SENP2, A20, Cul1/Rbx1 E3 ligase, YAP1/TAZ, MAP1LC3A, CDK6, ELAVL1 (HuR), PEG3, WNT5A, PKC-alpha, Porf-2, WNT, Beclin 1, NEDD4L, Cyclin D1, p53, NKD2, DACT3, Frizzled, Siah1/SIP/Ebi E3 ligase, DACT1
4	Neurophysiological process_Dynein-dynactin motor complex in axonal transport in neurons	8.348E-13	NudE, ERK1/2, DCTN1(p150Glued), Importin (karyopherin)-alpha, HDAC6, APP, DYNC1I1, Vimentin, Ubiquitin, Hap-1, TMEM108, Centractins, DYNLL, ORP1, Dynein 1, cytoplasmic, heavy chain, Tctex-1, Alpha-centractin, MAPRE3(EB3), DYNLT, HAP40, PRNP, AKT(PKB), Snapin, PAFAH alpha (LIS1), MAPRPE1(EB1), DYI2, Tubulin (in microtubules), Importin (karyopherin)-beta, Dynein 1, cytoplasmic, light chains, TrkC, Sortilin, BPAG1, TrkB, JSAP1, NGF, RILP (Rab interacting lysosomal protein), Kinesin heavy chain, Bassoon, Kinesin light chain, Carboxypeptidase H, Dynein 1, cytoplasmic, intermediate chains, BDNF, NUDEL, SPTBN2
5	G protein-coupled receptors signaling in lung cancer	1.444E-12	SSTR2, c-Fos, CD44, ERK1/2, PGE2R1, HB-EGF, VEGF-A, PKA-reg (cAMP-dependent), BDKRB2, Galanin, G-protein alpha-q/11, CCL5, I-kB, GRP-R, CaMK II, Amphiregulin, CNR1, IKK-alpha, GALR2, NTSR1, MMP-2, Bcl-XL, G-protein beta/gamma, Galpha(i)-specific peptide GPCRs, VIP, TGF-alpha, Galpha(i)-specific cannabis GPSRs, AKT(PKB), PDK (PDPK1), c-Src, SDF-1, CXCR4, RhoA, Pyk2(FAK2), PGE2R4, G-protein alpha-12 family, IL-8, G-protein alpha-12, EGFR, G-protein alpha-i family, Galpha(q)-specific peptide GPCRs, PGE2R3, HB-EGF(mature), SSTR3, STAT3, EDNRB, Calmodulin, GRP(1-27), NT, VIP receptor 1, Cyclin D1, VIP receptor 2, SSTR5, ADAM17, PKA-cat (cAMP-dependent), EDNRA
6	Transcription_Negative regulation of HIF1A function	2.066E-12	FHL3, p14ARF, Casein kinase I delta, Sirtuin3, MCM5, RUNX3, COMMD1 (MURR1), FBXW7, SART1, KLF2, VCP, Sirtuin6, SKP1, UBXD7, Ubiquitin, ING4, GSK3 beta, EGLN2, MCM7, Elongin B, LAMP2, EAF2, MDM2, MCM2, HSP90, Calpain 1(mu), HSP70, SAT2, EGLN1, RACK1, PLK3 (CNK), HSC70, PRDX2, CITED2, HIF1A, MTG16 (CBFA2T3), ARD1, PSMA7, Sirtuin2, HSP90 beta, Cul1/Rbx1 E3 ligase, AML1 (RUNX1), Sirtuin1, HIF3A, PTEN, SSAT, Elongin C, HIF-prolyl hydroxylase, PRDX4, CHIP, p53, CITED4
7	Chemotaxis_Lysophosphatidic acid signaling via GPCRs	4.803E-12	LPAR3, c-Fos, H-Ras, LARG, ROCK1, ERK1/2, PRK1, HB-EGF, Beta-catenin, EGR1, HDAC7, G-protein alpha-q/11, p21, PKC-epsilon, TRIP6, IP3 receptor, LPAR2, PI3K reg class IA (p85), Bax, GSK3 beta, PLC-beta, FKHR, PLEKHG2, DIA1, HAS2, Caspase-3, Cyr61, CREB1, Bcl-XL, MEK1/2, G-protein beta/gamma, CDC42, Actin cytoskeletal, N-CoR, TAZ, MSK1, AKT(PKB), PDK (PDPK1), c-Src, RhoA, Tiam1, E-cadherin, cPKC (conventional), G-protein alpha-12 family, PLC-eta 1, PKC-delta, Tcf(Lef), IL-8, EGFR, G-protein alpha-i family, F-Actin cytoskeleton, MKL2, mTORC1, Caspase-9, LIMK, PLD2, CD36, G-protein gamma 12, MEK4(MAP2K4), 4E-BP1, p130CAS, ROCK, PRKD1, PAK, PDZ-RhoGEF, PKC, Vinculin, ATF-2, PLC-delta 1, Bcl-2, FasR(CD95), SIVA1, Rho GTPase, MKL1, CTGF, p53, PLC-beta3, ADAM17, p38 MAPK, Elk-1, mTOR, PREX1

8	Development_Positive regulation of WNT/Beta-catenin signaling in the cytoplasm	7.694E-12	Casein kinase II, alpha chains, TBL1X, Bcl-9, BIG1, IRS-2, NKD1, IRS-1, UBE2B, PP1-cat, RIPK4, APC protein, Alpha-1 catenin, 14-3-3, Beta-catenin, TGIF, SMAD4, Makorin-1, Dsh, USP47, ZBED3, CDK1 (p34), PPP2R2A, SIAH1, USP25, PR130, Insulin receptor, GSK3 alpha/beta, IGF-1 receptor, ERK2 (MAPK1), AKT(PKB), Axin, HECTD1, ITGB1, SIAH2, HIPK2, Tcf(Lef), 14-3-3 zeta/delta, TGT, HSP105, PKA-reg type II (cAMP-dependent), PP2A catalytic, Trabid, BIG2, USP9X, WNT, Jouberin, PP2C alpha, SMAD3, Parathyroid hormone, NKD2, MITF, PKA-cat (cAMP-dependent), Frizzled, SET7, DACT1
9	Immune response_IFN-alpha/beta signaling via MAPKs	1.507E-11	IP10, ISG15, TCF7L2 (TCF4), RIG-G, ERK1/2, GCH1, Beta-catenin, Matrilysin (MMP-7), PRMT1, ZNF145, SMAD4, PIAS1, Apo-2L(TNFSF10), p21, IFNAR1, VAV-1, STAT1, PL scramblase 1, ULK1, IKK-epsilon, Ubiquitin, TAP1 (PSF1), MAPKAPK2, PKC-theta, IFN-alpha, ISG54, Axin2, p130, MSK1, RSAD2, AKT(PKB), p27KIP1, FZD7, HIP-2, Lck, CD45, PKR, PKC-delta, MEK3(MAP2K3), IRF9, MEK4(MAP2K4), Ku80, MEKK1(MAP3K1), SP5, Filamin B (TABP), FOXO3A, FasR(CD95), IRF7, Cyclin D1, HDAC1, SMAD3, p38 MAPK, mTOR
10	Immune response_Antigen presentation by MHC class I: cross-presentation	2.580E-11	CLEC12A, Fc epsilon RI gamma, MSR1, Endoplasmin, gp91-phox, FCGR3A, Rac2, CLEC4C, VCP, TAP, Rab-3C, VAV-1, p47-phox, Syk, Rab-6, IP-30, Adipophilin, HYOU1, IRAP, MyD88, C1q, cPLA2, Calreticulin, MHC class I, HSP60, TLR9, HSP90, Syntaxin 4, HSP70, Rab8B, LRP1, EHD1, Cathepsin L, Dectin-1, Rab-4A, SEC61 complex, FCGRT, LLIR, Cytochrome b-558, CLEC9A, HSP105, Rab-32, Fc gamma RII alpha, Rab-35, RanBPM, Rab-33A, Cathepsin B, HSPA1A, SEC22B, Fc gamma RI, DAP12, CD74, Rab-11A, CD8, UFO, Rab-10, OLR1, IKK-beta, TLR7, CD40(TNFRSF5), RAB-5B, TRIF (TICAM1), CHIP, SNAP-23, Rab-34, TLR2
11	HGF signaling in melanoma	8.917E-11	GAB1, CD44, ERK1/2, Beta-catenin, EGR1, Fibronectin, PI3K reg class IA (p85), GSK3 beta, N-Ras, Bcl-XL, TWIST1, AKT(PKB), alpha-MSH, MC1R, HGF, MEK1(MAP2K1), N-cadherin, E-cadherin, PI3K cat class IA, Desmoglein 1, HIF1A, C/EBPbeta, RXRA, PKC-beta2, PKC-alpha, ATF-2, SLUG, Cyclin D1, Plakoglobin, HGF receptor (Met), p38 MAPK, Elk-1, MITF
12	CHDI_Correlations from Replication data_Causal network (positive correlations)	9.062E-11	MHC class II, IL-1 alpha, Calcineurin A (catalytic), ICAM1, CD44, CD28, MSK1/2 (RPS6KA5/4), HDAC7, IP3 receptor, I-kB, PI3K reg class IA (p85), PKC-theta, MyD88, IKK-alpha, PLC-gamma 1, NR2, ITK, Caspase-3, IL-1RI, CREB1, G-protein beta/gamma, CD83, NF-kB, AKT(PKB), HSP70, SDF-1, CXCR4, RhoA, Pyk2(FAK2), CD80, PI3K cat class IA, Lck, CD45, Slp76, HIP1, MEK3(MAP2K3), PSMC2, MEF2, NIK(MAP3K14), LAT, NR2A, RhoGDI alpha, MEK4(MAP2K4), ROCK, Calmodulin, PKC-alpha, CD3, IKK-beta, IP3R1, PSD-95, IRAK1/2, CD40(TNFRSF5), Calcineurin B (regulatory), p38 MAPK, TLR2
13	Neurogenesis_NGF/ TrkA MAPK-mediated signaling	9.356E-11	SPHK1, NEFL, c-Fos, Fra-1, H-Ras, ERK1/2, MEF2C, HB-EGF, EGR1, PP2A regulatory, SGK1, PKA-reg (cAMP-dependent), FRS2, p21, VGF, PKC-epsilon, Ephrin-A receptor 2, IP3 receptor, TY3H, MAPKAPK2, PLC-gamma 1, CalDAG-GEFII, N-Ras, SNX26 (TCGAP), MAP2K5 (MEK5), KIDINS220, CREB1, MEK1/2, PLAUR (uPAR), Flotillin-1, M-Ras, MSK1, RGS2, p107, Stromelysin-1, c-Src, GAB2, Sequestosome 1(p62), SHPS-1, MATK, Efs/Sin, RIN, NF-kB1 (p50), K-RAS, PKC-delta, MEK3(MAP2K3), SOS, PP2A catalytic, C3G, SH2B, p90Rsk, p130CAS, NGF, Calmodulin, MAGI-2, SHP-2, SUR-8, Cyclin D1, SORBS1, SP1, PVR, JMJD3, SHB, RIT, p38 MAPK, Elk-1, PKA-cat (cAMP-dependent), FosB
14	Role of tumor-infiltrating B cells in anti-tumor immunity	1.033E-10	MIG, IL-18R1, MHC class II, IP10, CTAG2, DHFR, Granzyme B, IFN-gamma, MAGE-1 antigen, Apo-2L(TNFSF10), CD19, Dsk2 (ubiquilin-2), Perforin, STAT1, MAGE-3, IFN-alpha, ACAT2, CD4, CD27(TNFRSF7), MHC class I, MAGE-4 antigen, G-protein beta/gamma, NXF2, Kappa chain (Ig light chain), MAGEB2, FasL(TNFSF6), NF-kB, CT47A, SDF-1, CXCR4, IL-2, CD40L(TNFSF5), G3P2, Syndecan-1, MAGEC1, GAS11, CD38, I-TAC, T-bet, G-protein alpha-i family, AID, IL4RA, Bcl-6, IRF4, RGS1, Btk, CXCR3, STAT3, CD8, CXorf61, SOX2, ATF-2, FasR(CD95), CXCL13, KTN1, CD40(TNFRSF5), STAT6, MAGEC2, p53, IL-21, BLIMP1 (PRDI-BF1)
15	IL-6 signaling in breast cancer cells	1.105E-10	gp130, IP10, c-Fos, GAB1, ESR1 (nuclear), H-Ras, ERK1/2, HSD17B1, Vimentin, Carbonic anhydrase IX, MDR1, STAT1, MUC1, Survivin, PI3K reg class IA, CYP19, IL-6, Bcl-XL, MEK1/2, TWIST1, AKT(PKB), c-Myc, N-cadherin, E-cadherin, PI3K cat class IA, NF-kB1 (p50), C/EBPbeta, JAB1, SOS, IL6RA, STAT3, SHP-2, Bcl-2, Fascin, S100A7, AKT2, Cyclin D1, SNAIL1, Jagged1, IL-6 receptor, C/EBPdelta
